# Supplementary material for: Efflux pump-mediated resistance to antifungal compounds can be prevented by conjugation with triphenylphosphonium cation
Source: Nat Commun. 2018 Nov 30;9:5102. doi: 10.1038/s41467-018-07633-9 (PMC6269435; doi:10.1038/s41467-018-07633-9)
Supplement: Supplementary file 1 — Supplementary Information [file 41467_2018_7633_MOESM1_ESM.pdf]

Efflux pump-mediated resistance to antifungal compounds can be prevented by conjugation with triphenylphosphonium cation

Chang et al.

(Tp-1) (Tp-2) (Tp-3) (Tp-4) (Tp-5) (Tp-6)

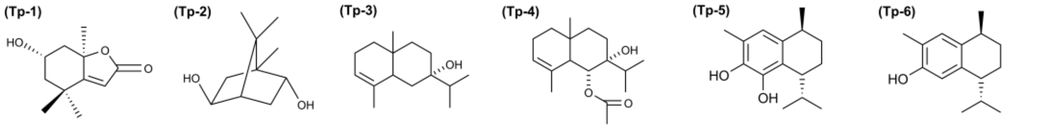

(Tp-7) (Tp-8) (Tp-9) (Tp-10) (Tp-11) (Tp-12)

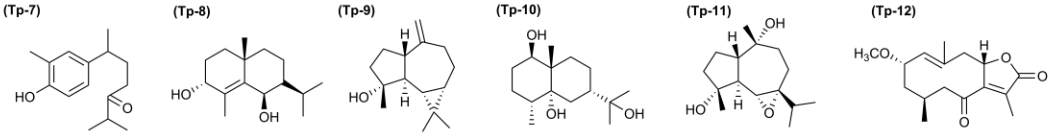

(Tp-13) (Tp-14) (Tp-15) (Tp-16) (Tp-17) (Tp-18)

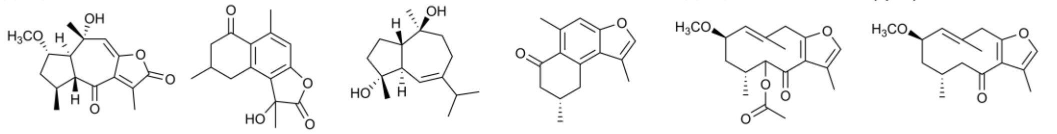

(Tp-19) (Tp-20) (Tp-21) (Tp-22) (Tp-23) (Tp-24)

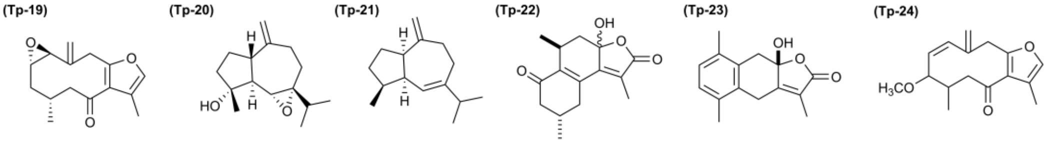

(Tp-25) (Tp-26) (Tp-27) (Tp-28) (Tp-29) (Tp-30)

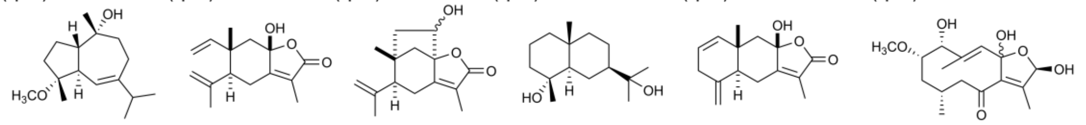

(Tp-31) (Tp-32) (Tp-33) (Tp-34) (Tp-35) (Tp-36)

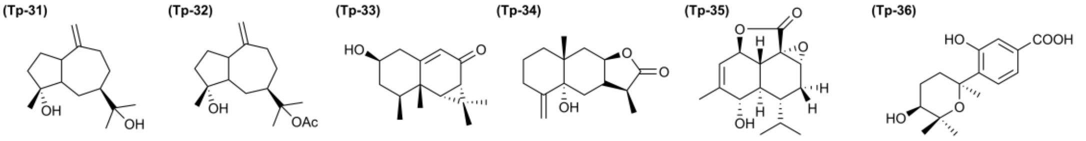

(Tp-37) (Tp-38) (Tp-39) (Tp-40) (Tp-41) (Tp-42)

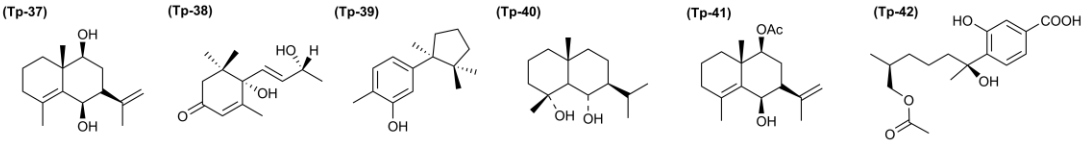

(Tp-43) (Tp-44) (Tp-45) (Tp-46) (Tp-47) (Tp-48)

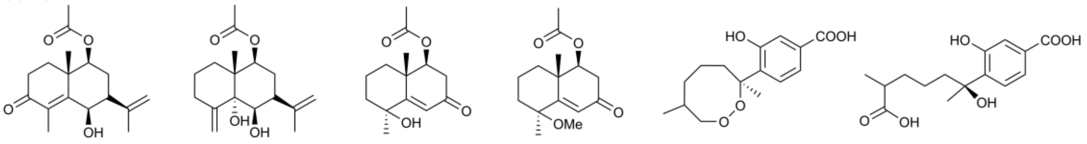

(Tp-49) (Tp-50) (Tp-51) (Tp-52) (Tp-53) (Tp-54)

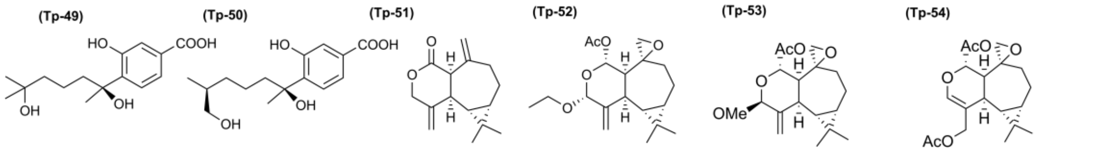

(Tp-55)

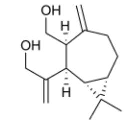

(Tp-56)

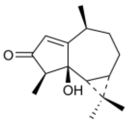

(Tp-57)

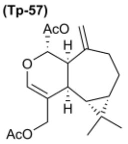

(Tp-58)

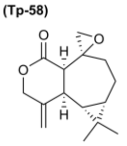

(Tp-59)

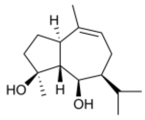

(Tp-60)

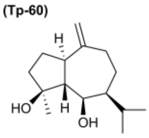

(Tp-61)

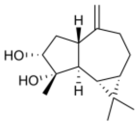

(Tp-62)

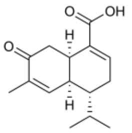

(Tp-63)

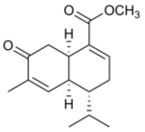

(Tp-64)

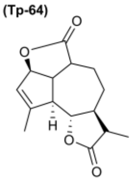

(Tp-65)

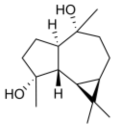

(Tp-66)

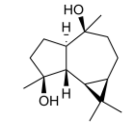

(Tp-67)

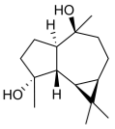

(Tp-68)

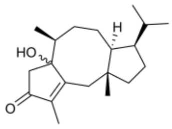

(Tp-69)

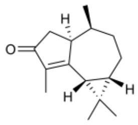

(Tp-70)

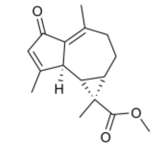

(Tp-71)

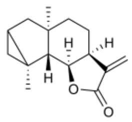

(Tp-72)

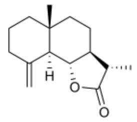

(Tp-73)

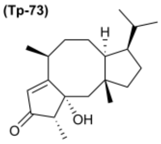

(Tp-74)

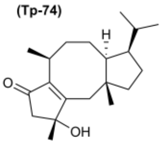

(Tp-75)

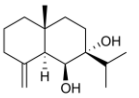

(Tp-76)

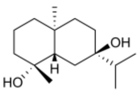

(Tp-77)

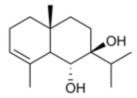

(Tp-78)

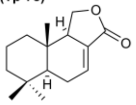

(Tp-79)

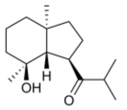

(Tp-80)

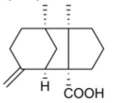

(Tp-81)

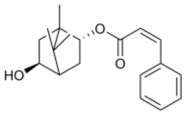

(Tp-82)

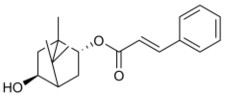

(Tp-83)

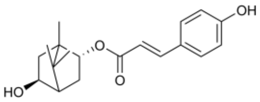

(Tp-84)

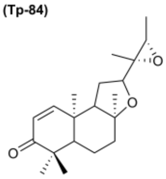

(Tp-85) (Tp-86)

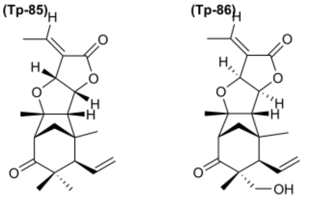

(Tp-87)

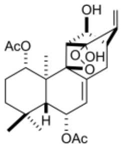

(Tp-88)

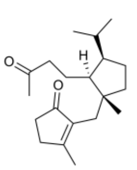

(Tp-89)

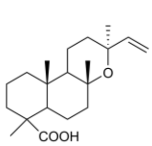

(Tp-90)

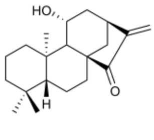

(Tp-91)

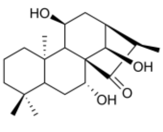

(Tp-92)

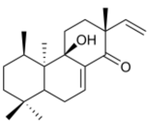

(Tp-93)

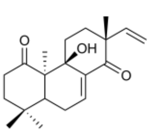

(Tp-94)

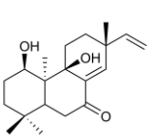

(Tp-95)

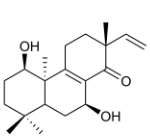

(Tp-96) (Tp-97) (Tp-98) (Tp-99) (Tp-100) (Tp-101)

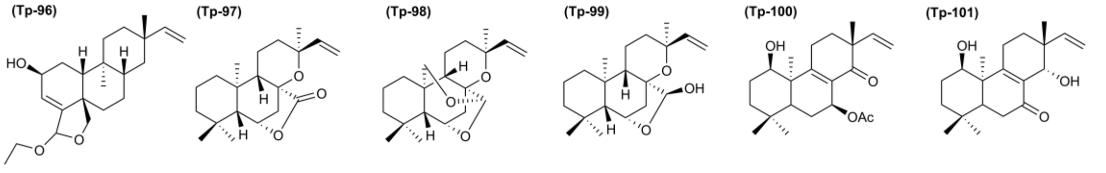

(Tp-102) (Tp-103) (Tp-104) (Tp-105) (Tp-106) (Tp-107)

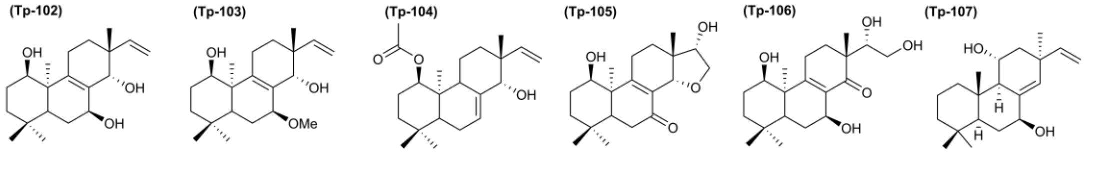

(Tp-108) (Tp-109) (Tp-110) (Tp-111) (Tp-112) (Tp-113)

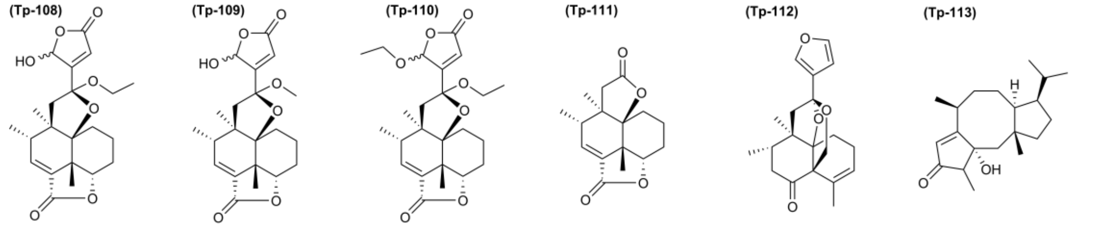

(Tp-114) (Tp-115) (Tp-116) (Tp-117) (Tp-118) (Tp-119)

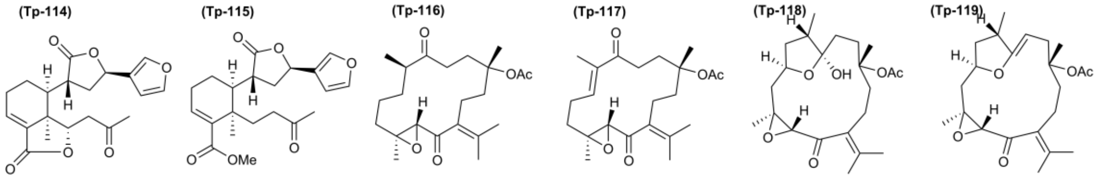

(Tp-120) (Tp-121) (Tp-122) (Tp-123) (Tp-124) (Tp-125)

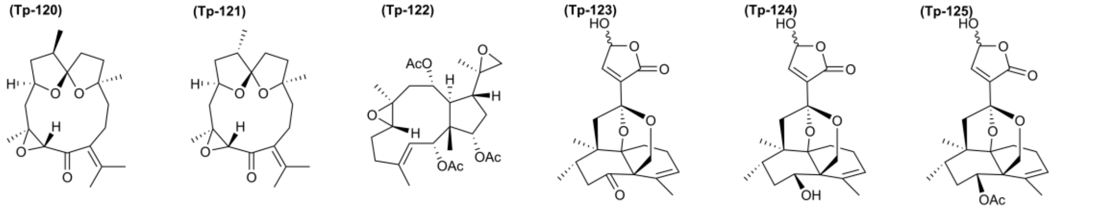

(Tp-126) (Tp-127) (Tp-128) (Tp-129) (Tp-130) (Tp-131)

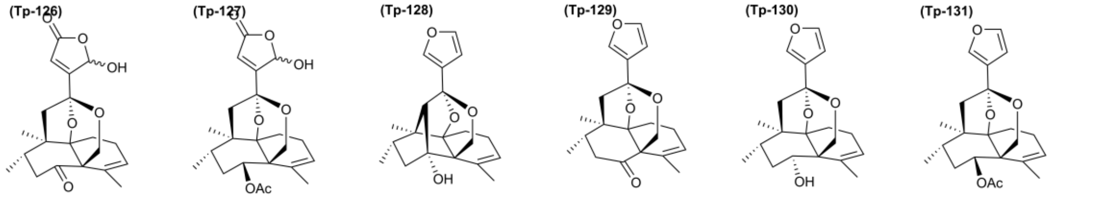

(Tp-132) (Tp-133) (Tp-134) (Tp-135) (Tp-136) (Tp-137)

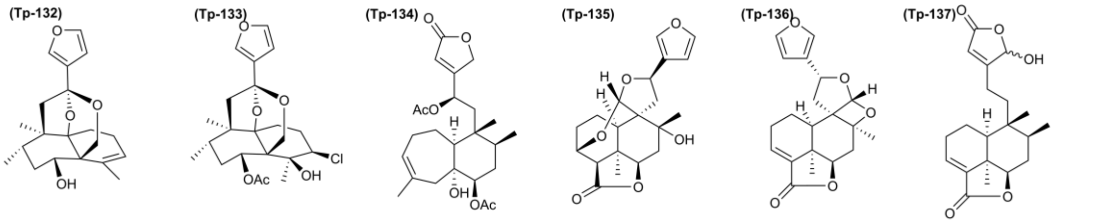

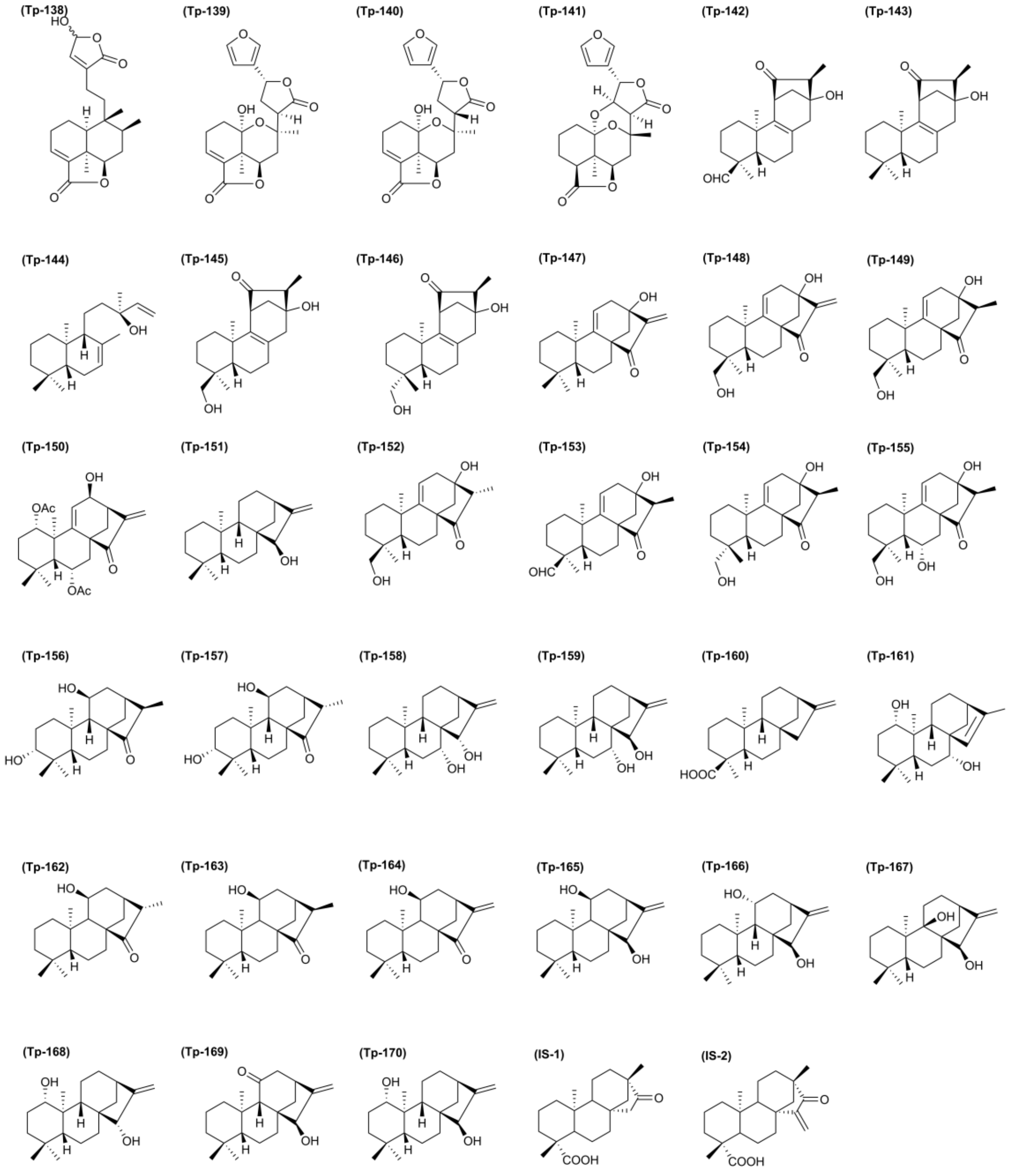

Supplementary Figure 1. The natural product library of 170 terpenoids and two semi-synthesized diterpenoids screened in this study.

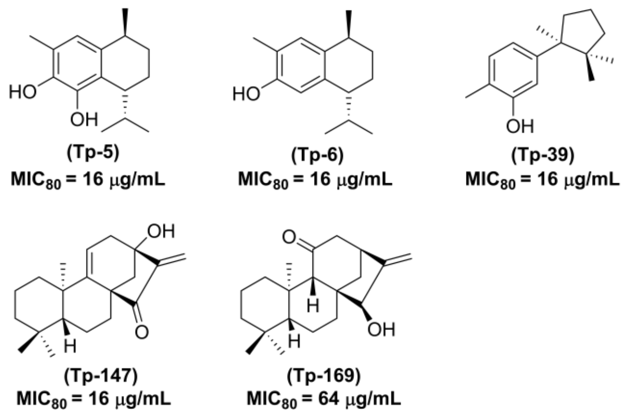

Supplementary Figure 2. Chemical structures of natural compounds that are active against both wild-type and efflux pump-deficient strain.

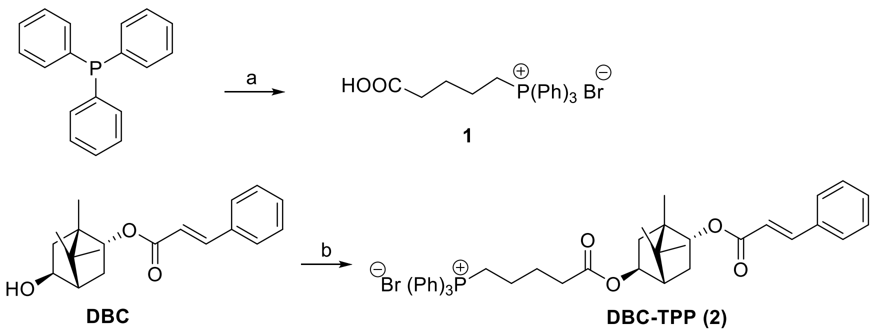

Supplementary Figure 3. General procedure for synthesis of DBC-TPP conjugate. Reagents and conditions: (a) 5-Bromovaleric acid, toluene; (b) EDCI, DMAP, DMF.

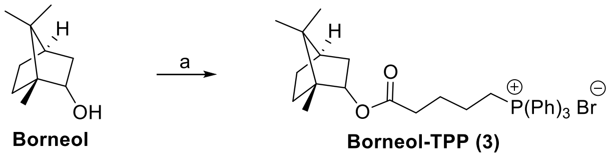

Supplementary Figure 4. General procedure for synthesis of Borneol-TPP conjugate. Reagents and conditions: (a) EDCI, DMAP, DMF.

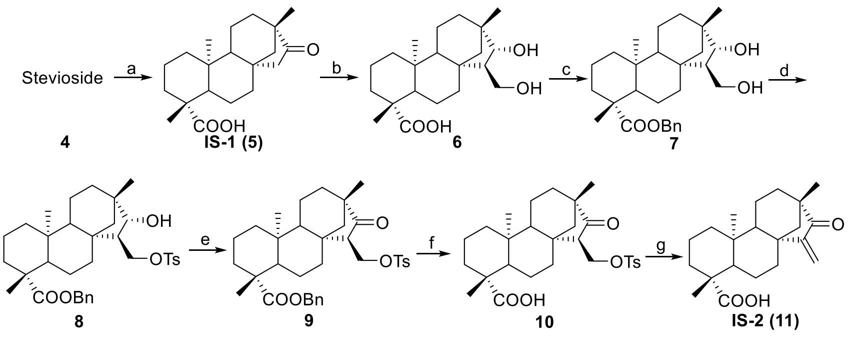

Supplementary Figure 5. General procedure for construction of α,β-unsaturated ketone on Isosteviol D-ring. Reagents and conditions: (a) 10 % H_2SO_4; (b) NaOH, 37 % HCHO; (c) K_2CO_3, BnBr, KI, DMF; (d) TsCl, DMAP; (e) PCC, DCM; (f) H_2, Pd-C; (g) DMAP, Py.

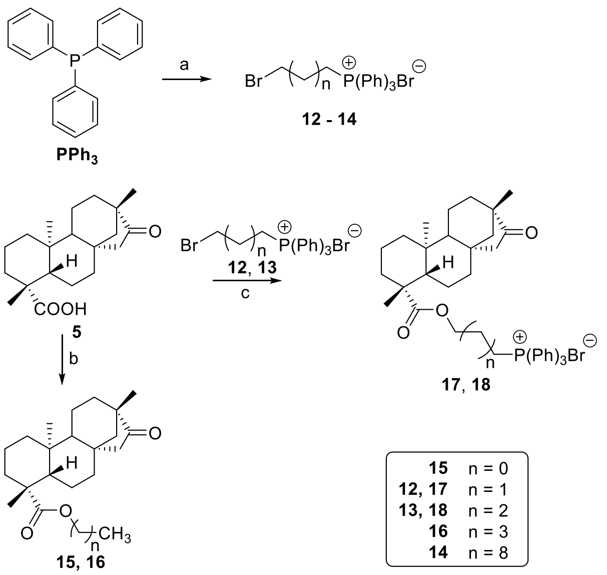

Supplementary Figure 6. General procedure for synthesis of TPP^+-conjugates with Isosteviol. Reagents and conditions: (a) α,ω-dibromoalkane, toluene; (b) K_2CO_3, MeI, DMF; (c) K_2CO_3, DMF.

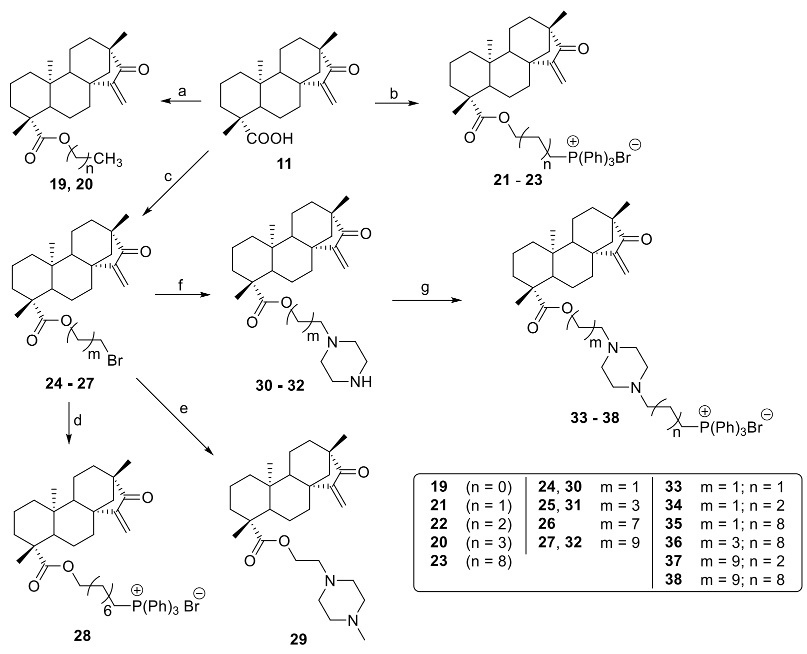

Supplementary Figure 7. General procedure for synthesis of TPP$^+$-conjugates with Isosteviol derivatives. Reagents and conditions: (a) K_2CO_3, DMF; (b) K_2CO_3, DMF; (c) α,ω-dibromoalkane, K_2CO_3, DMF; (d) PPh_3, toluene; (e) 1-Methylpiperazine, K_2CO_3, DMF; (f) Piperazine, K_2CO_3, DMF; (g) K_2CO_3, DMF.

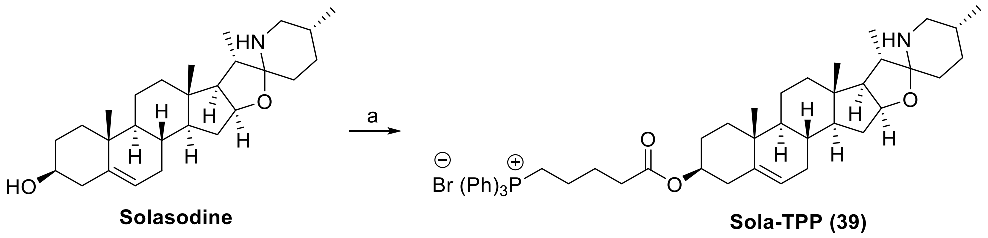

Supplementary Figure 8. General procedure for synthesis of Sola-TPP conjugate. Reagents and conditions: (a) HBTU, DIEA, DMF.

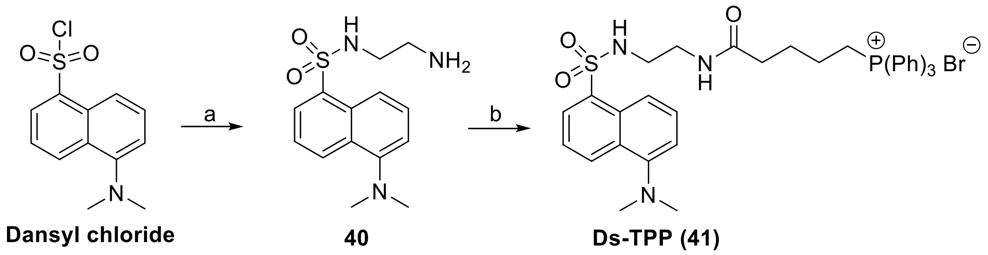

Supplementary Figure 9. General procedure for synthesis of Ds-TPP conjugate. Reagents and conditions: (a) EDA, DCM; (b) HBTU, DIPEA, DMF.

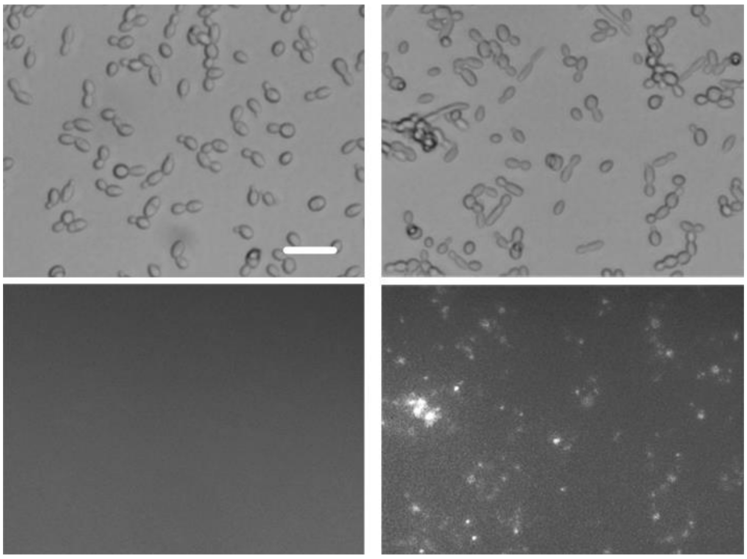

Supplementary Figure 10. The fluorescence microscopic observation revealed that Ds-TPP accumulated in *C. albicans* cells cultured in SD medium, whereas its parent compound dansyl chloride did not. Scale bar, 100 μm.

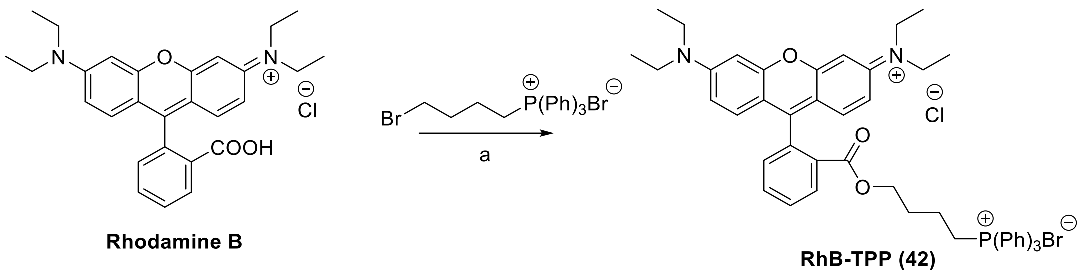

Supplementary Figure 11. General procedure for synthesis of RhB-TPP conjugate. Reagents and conditions: (a) K_2CO_3, DMF.

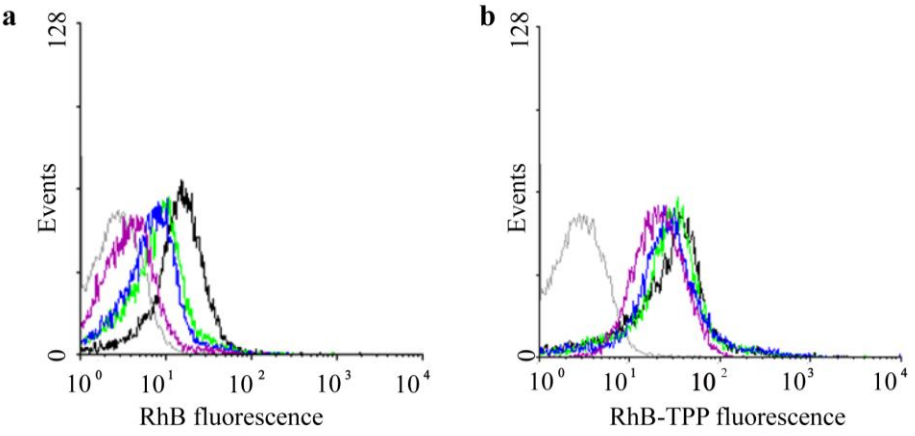

Supplementary Figure 12. The intracellular RhB or RhB-TPP contents in different *C. albicans* strains. **a-b** Four *C. albicans* strains including *cdr1* and *cdr2* double mutant DSY654, wild type strain SC5314, YEM13 with hyperexpression of *MDR1*, YEM15 with hyperexpression of *CDR1*and *CDR2* were diluted to 10^6 cells/mL using RPMI1640 medium and incubated with 20 μg/mL RhB (**a**) or RhB-TPP (**b**) at 30 °C for 2 h. The intracellular contents were detected by flow cytometry based on the fluorescence intensity. Grey line, *C. albicans* cells without staining; black line, *C. albicans* DSY654 treated with RhB or RhB-TPP; green line, *C. albicans* SC5314 treated with RhB or RhB-TPP; blue line, *C. albicans* YEM13 treated with RhB or RhB-TPP; purple line, *C. albicans* YEM15 treated with RhB or RhB-TPP.

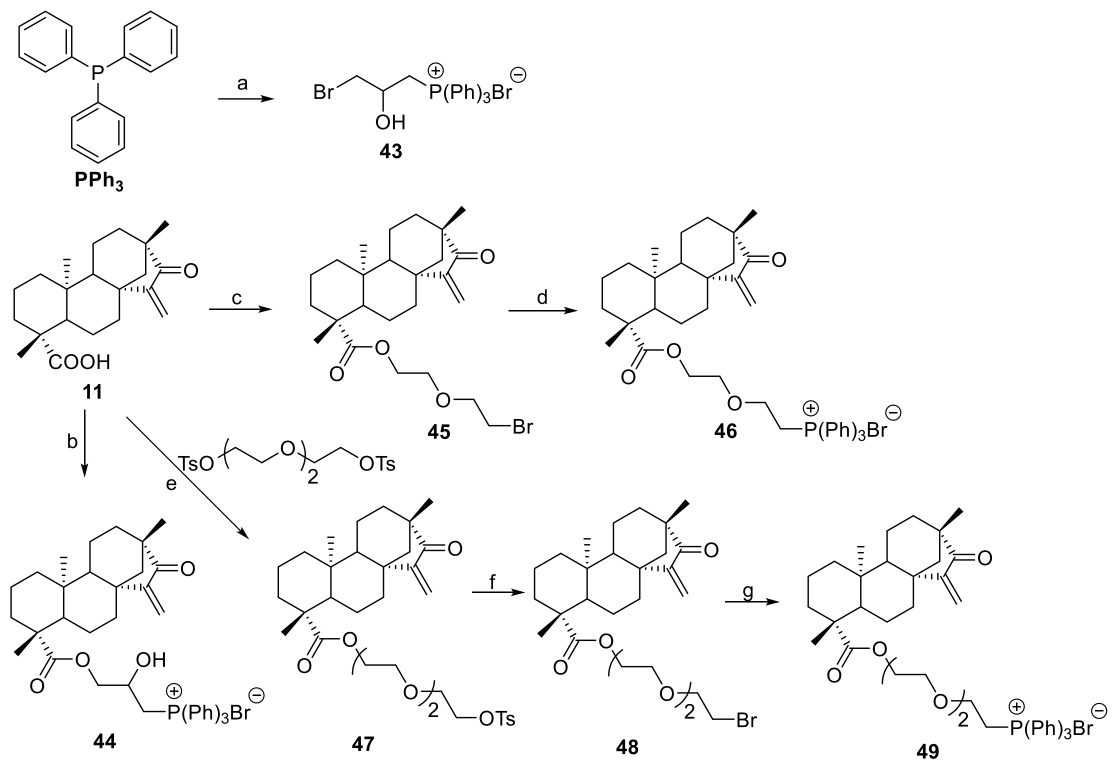

Supplementary Figure 13. General procedure for synthesis of TPP^{+}-conjugates with PEG as a linker. Reagents and conditions: (a) 1,3-Dibromo-2-propanol, toluene; (b) K_2CO_3, DMF; (c) Bis(2-bromoethyl) ether, K_2CO_3, DMF; (d) PPh_3, toluene; (e) Triethylene Glycol Bis(p-toluenesulfonate), K_2CO_3, DMF; (f) LiBr, Acetone; (g) PPh_3, toluene.

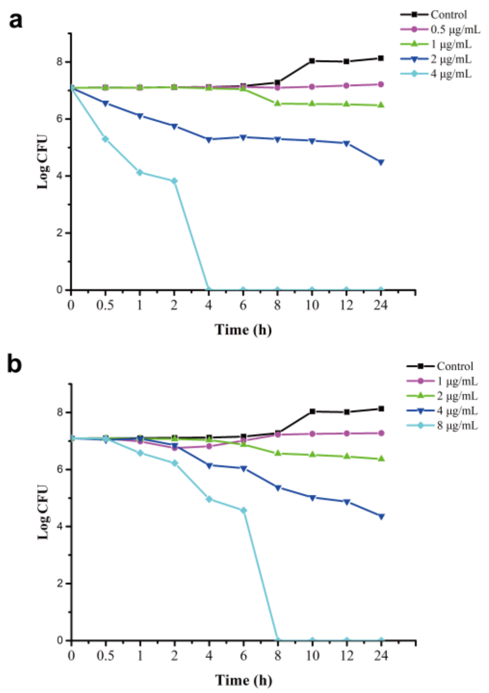

Supplementary Figure 14. Time-killing kinetics of IS-2-Pi-TPP (**a**) and AMB (**b**) against *C. albicans* SC5314. Data are representative of three independent experiments.

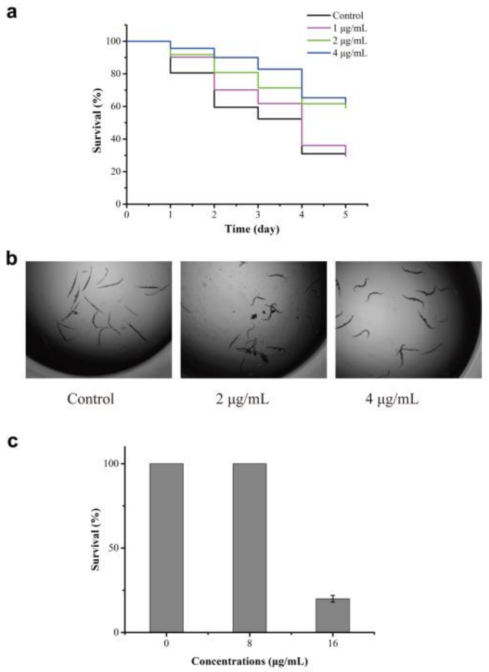

Supplementary Figure 15. Antifungal activity assay for IS-2-Pi-TPP in *C. elegans* infected by *C. albicans*. **a** Survival curves of nematodes infected with *C. albicans* SC5314. Each day, the worms were monitored, and the survival rate was calculated. (n=71 in control group, n=70 in IS-2-Pi-TPP (1 μg/mL)-treated group, n=73 in IS-2-Pi-TPP (2 μg/mL)-treated group, n=69 in IS-2-Pi-TPP (4 μg/mL)-treated group). The log-rank (Mantel-Cox) test was performed. ****P* <0.001 for drug-treated (2 and 4 μg/mL) groups versus control group. **b** After 5 days of treatment, nematodes were imaged using an Olympus microscope. **c** The toxicity of IS-2-Pi-TPP to *C. elegans.* Healthy nematodes were incubated with serial concentrations of IS-2-Pi-TPP for 5 days, and the survival state was monitored by microscopic observation.

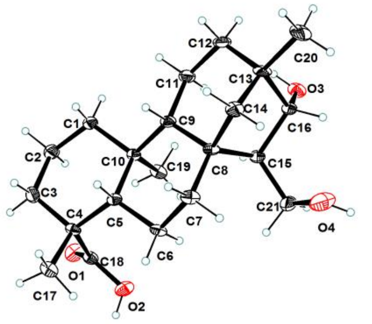

Supplementary Figure 16. X-ray crystallographic structure of compound **6**.

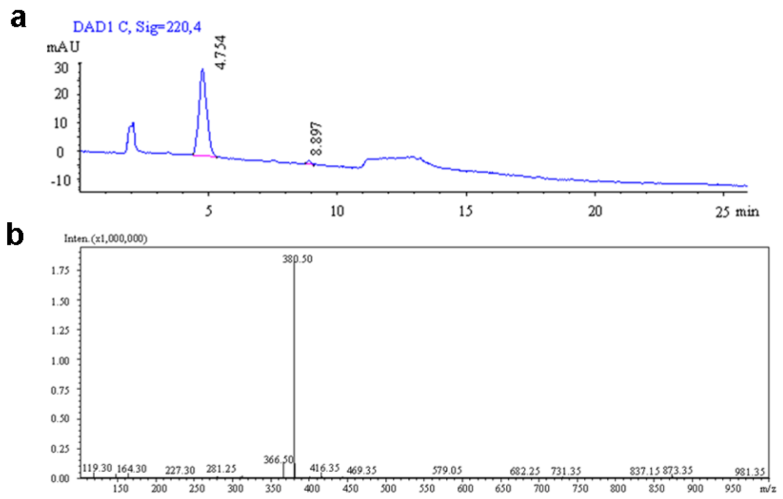

Supplementary Figure 17. a HPLC chromatogram of RhB-TPP. **b** ESI-MS spectrum of RhB-TPP.

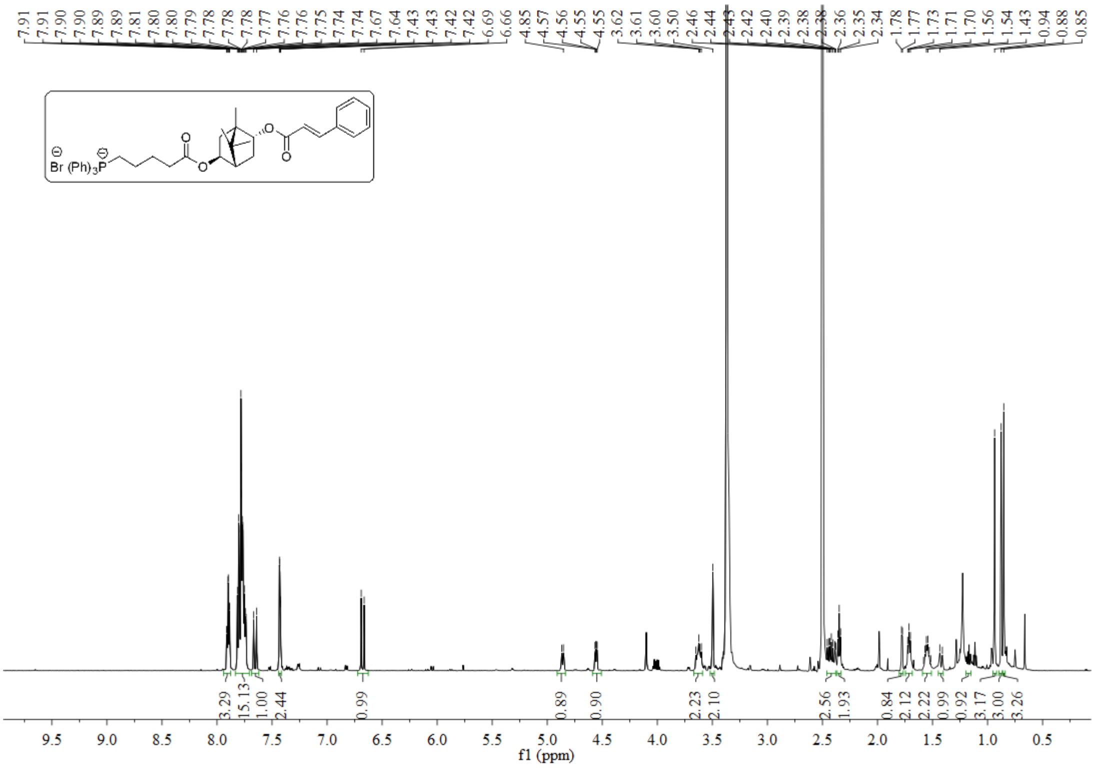

Supplementary Figure 18. ^{1}H NMR spectrum of **2**

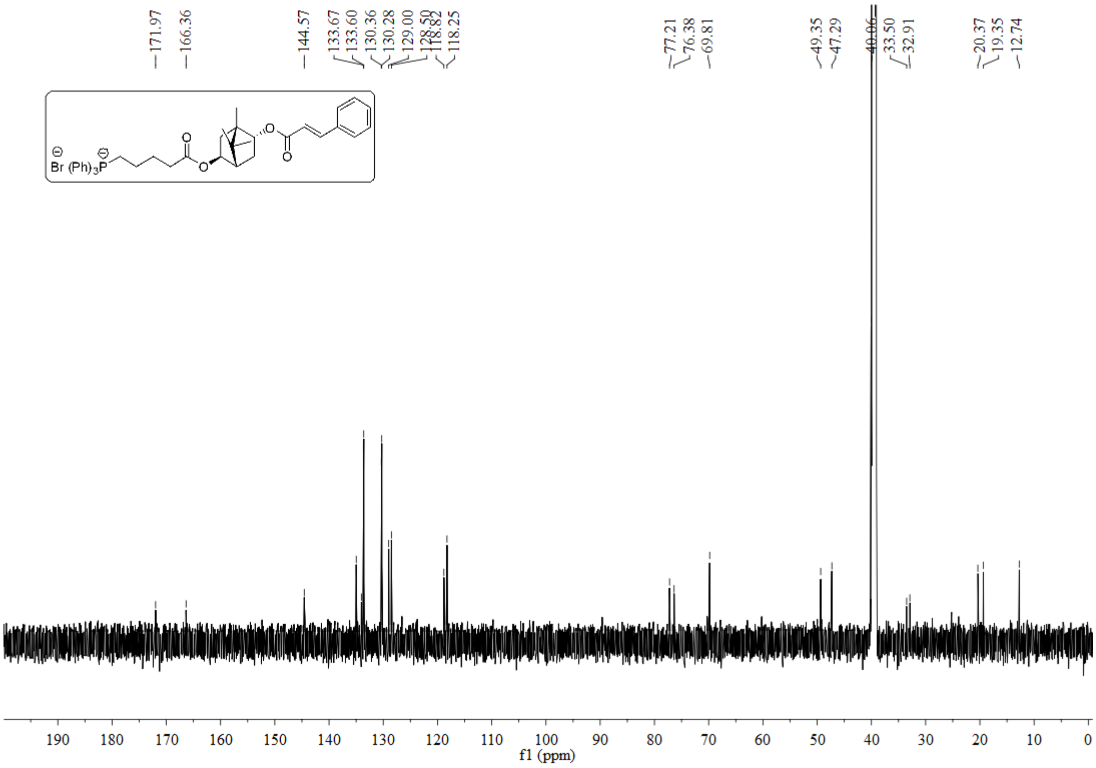

Supplementary Figure 19. ^{13}C NMR spectrum of **2**

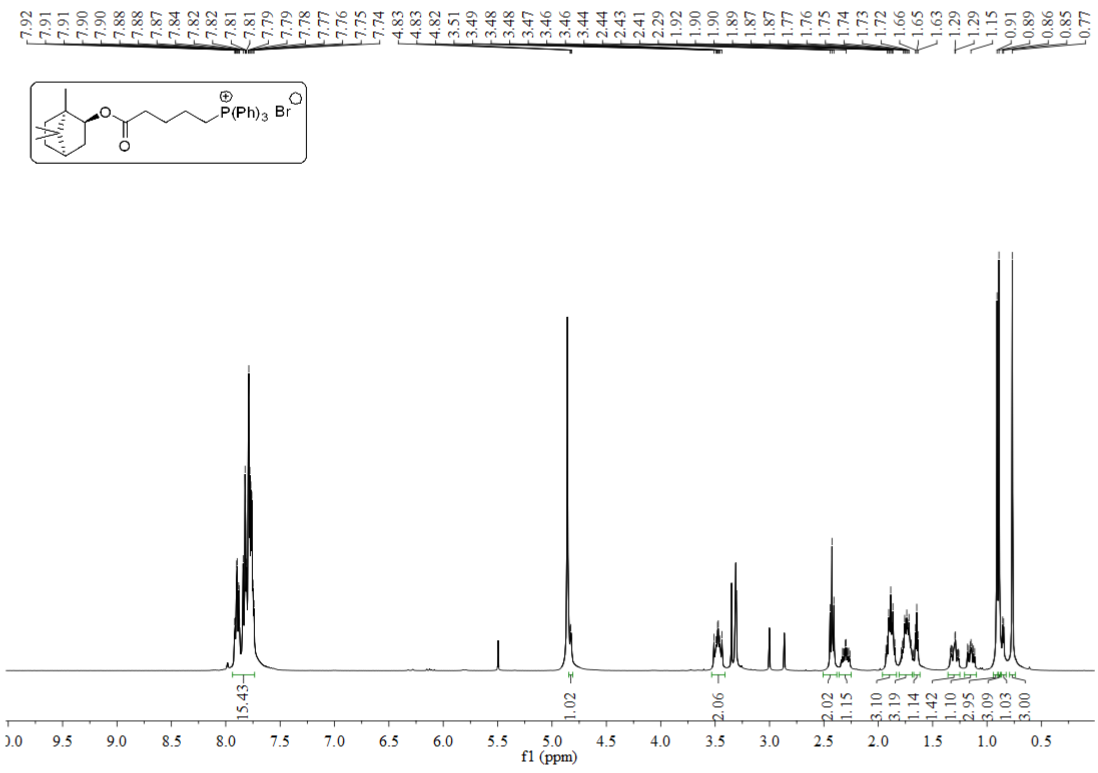

Supplementary Figure 20. ^{1}H NMR spectrum of **3**

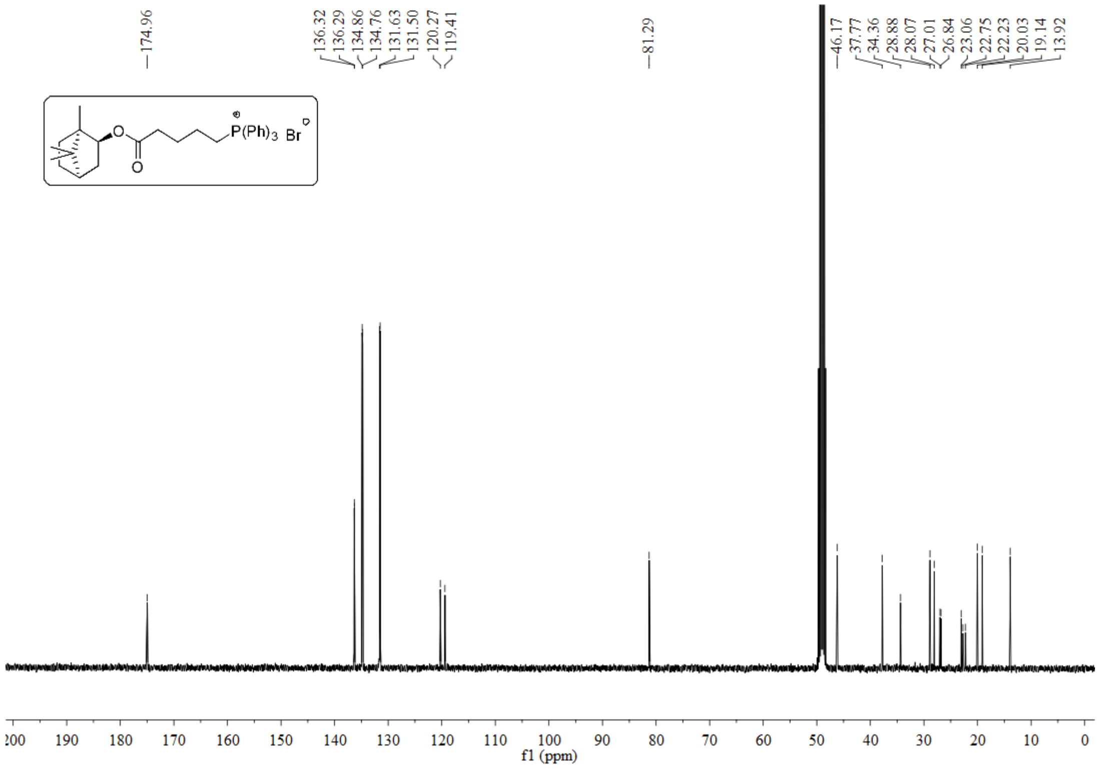

Supplementary Figure 21. ^{13}C NMR spectrum of **3**

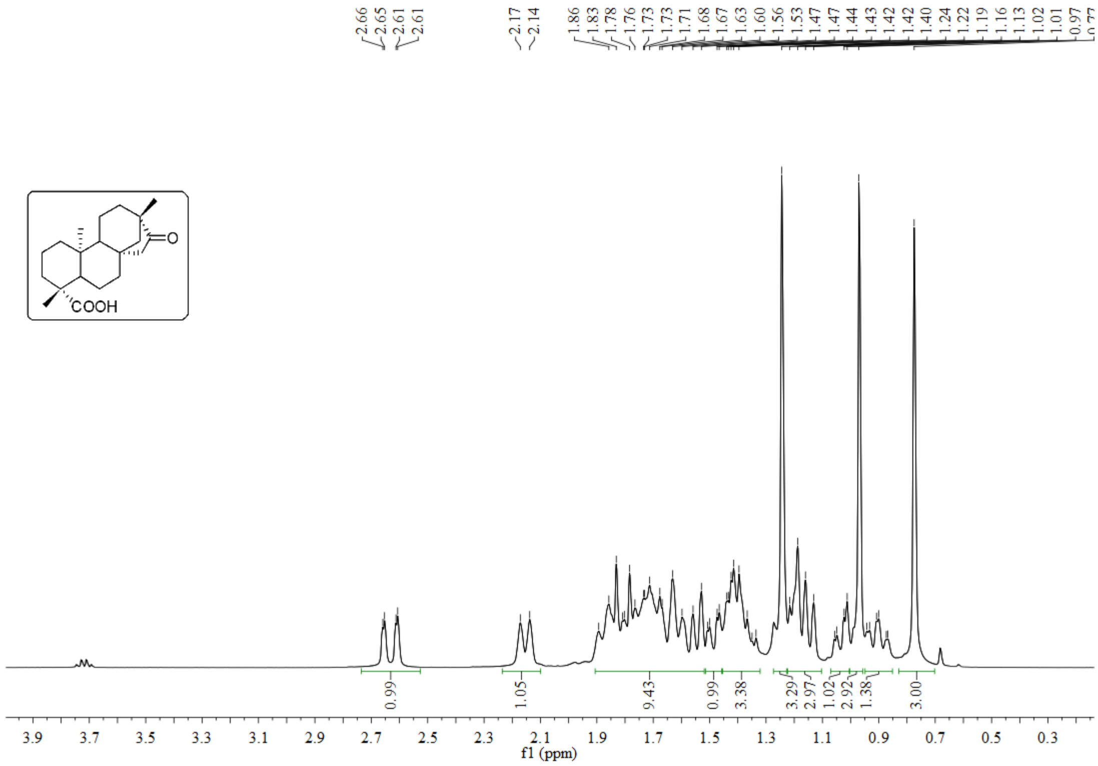

Supplementary Figure 22. ^{1}H NMR spectrum of **5**

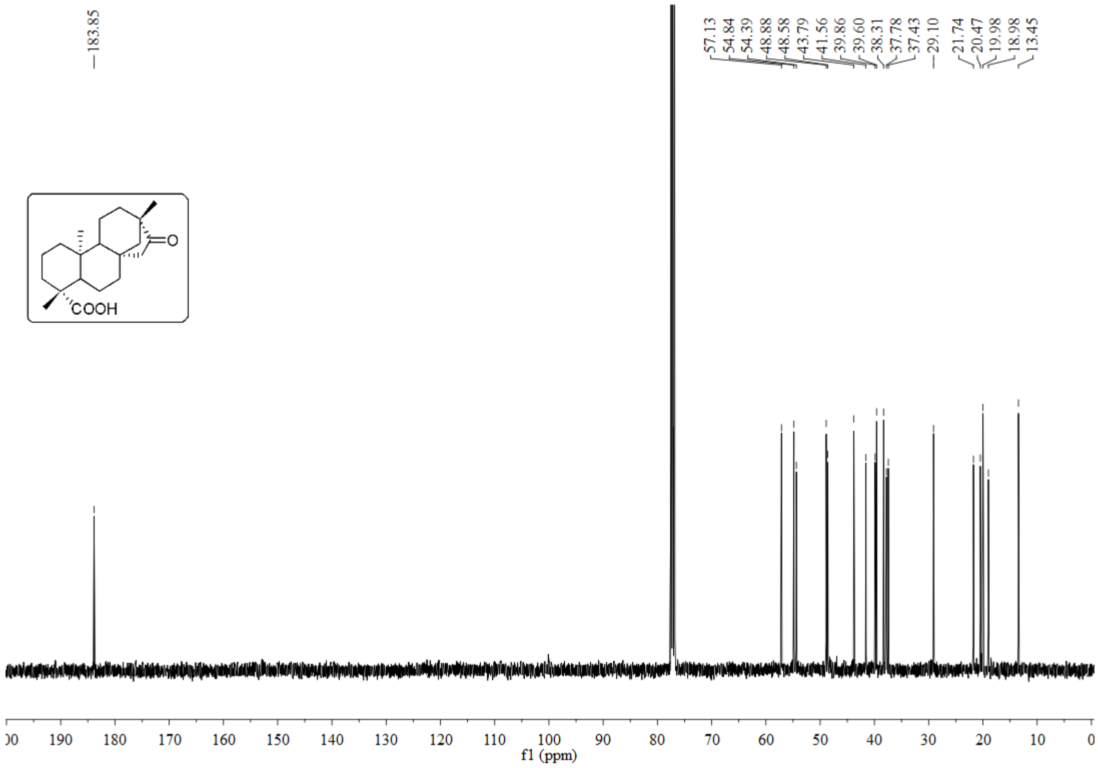

Supplementary Figure 23. ^{13}C NMR spectrum of **5**

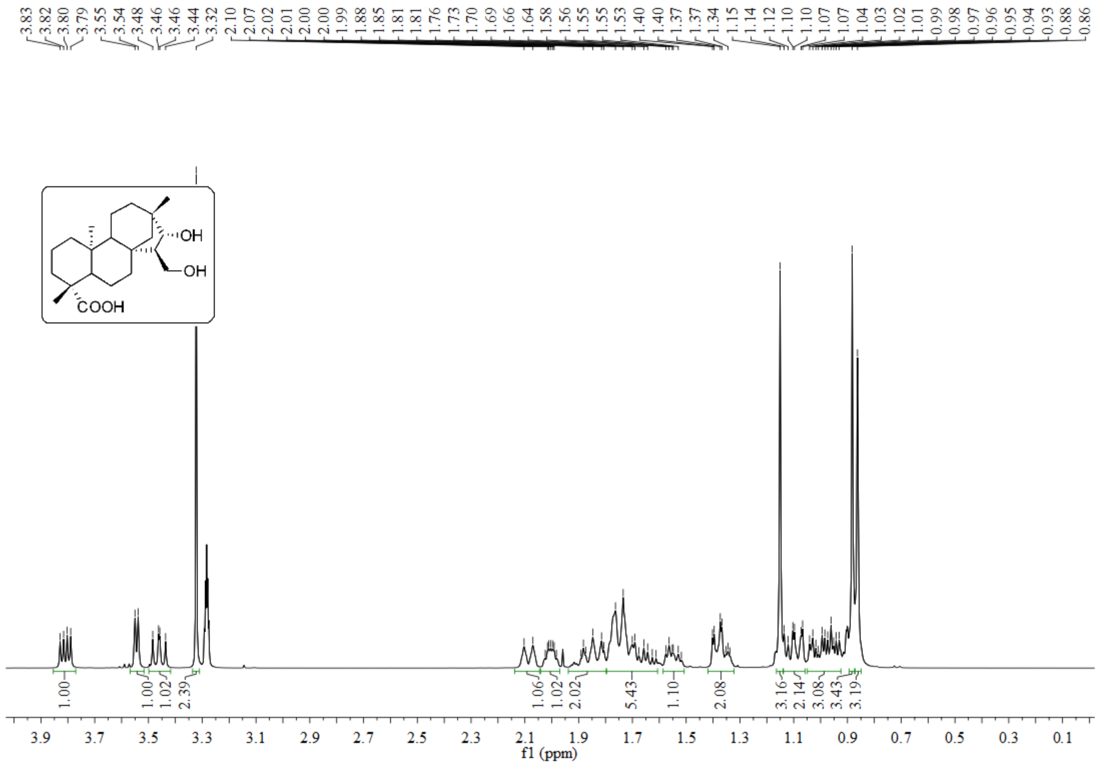

Supplementary Figure 24. ^{1}H NMR spectrum of **6**

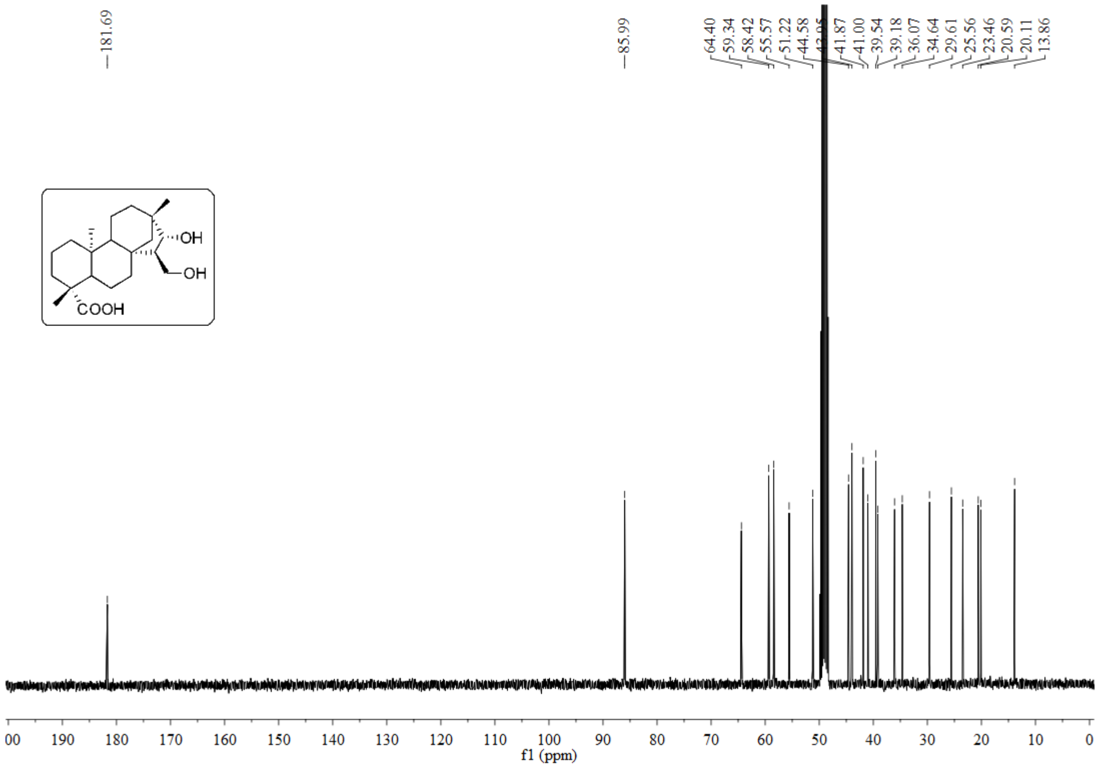

Supplementary Figure 25. ^{13}C NMR spectrum of **6**

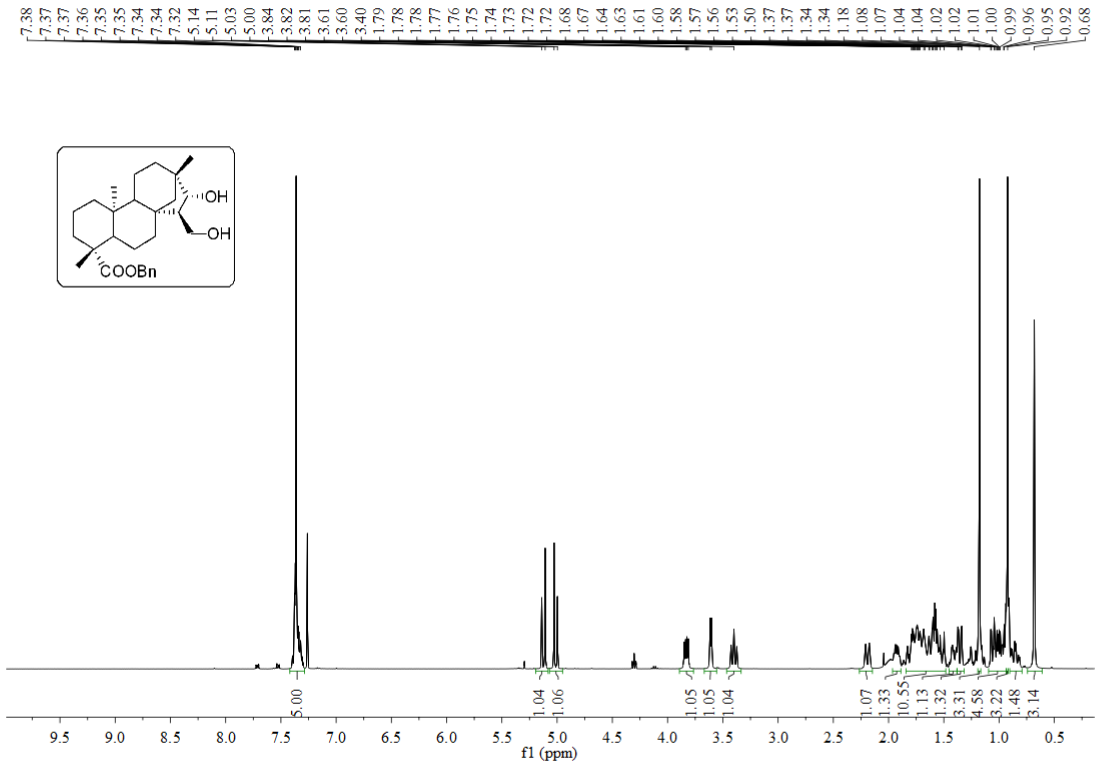

Supplementary Figure 26. ^{1}H NMR spectrum of **7**

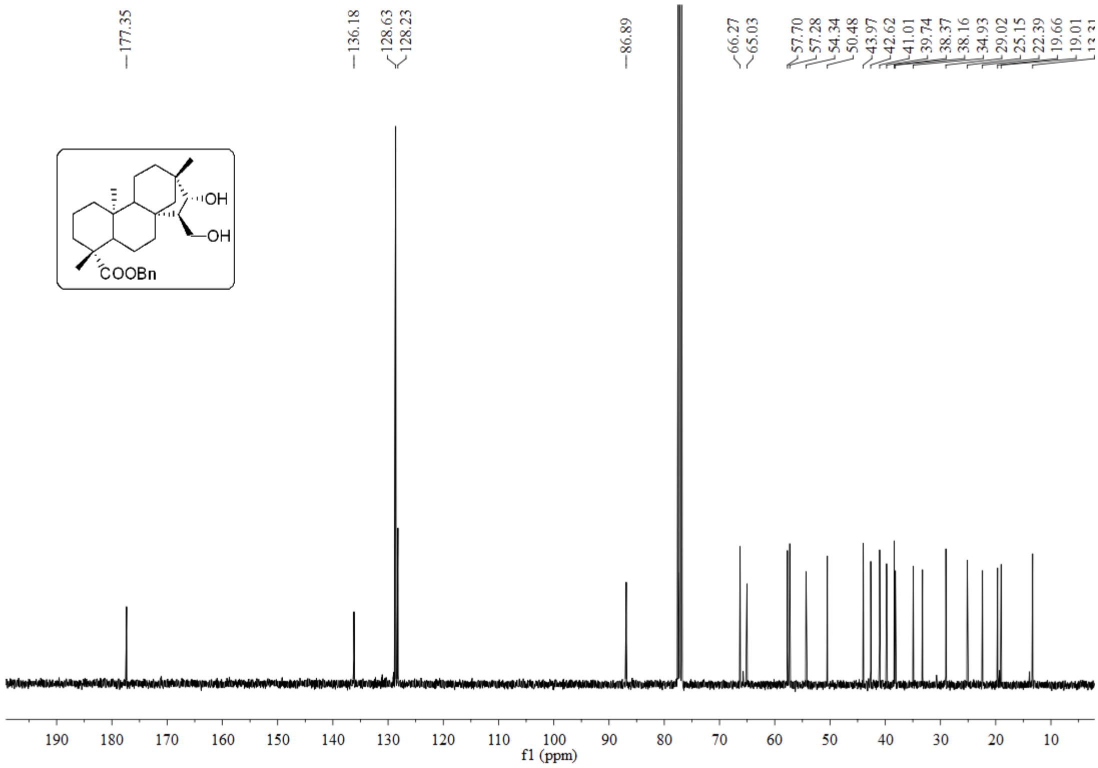

Supplementary Figure 27. ^{13}C NMR spectrum of **7**

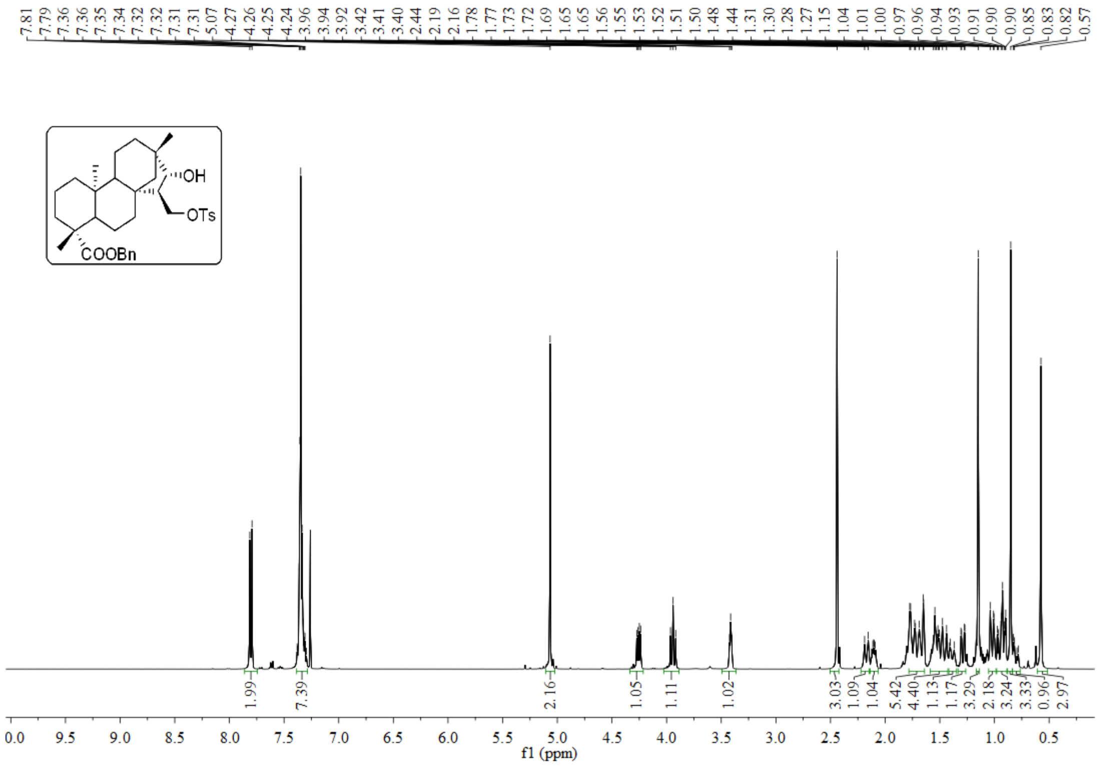

Supplementary Figure 28. ^{1}H NMR spectrum of **8**

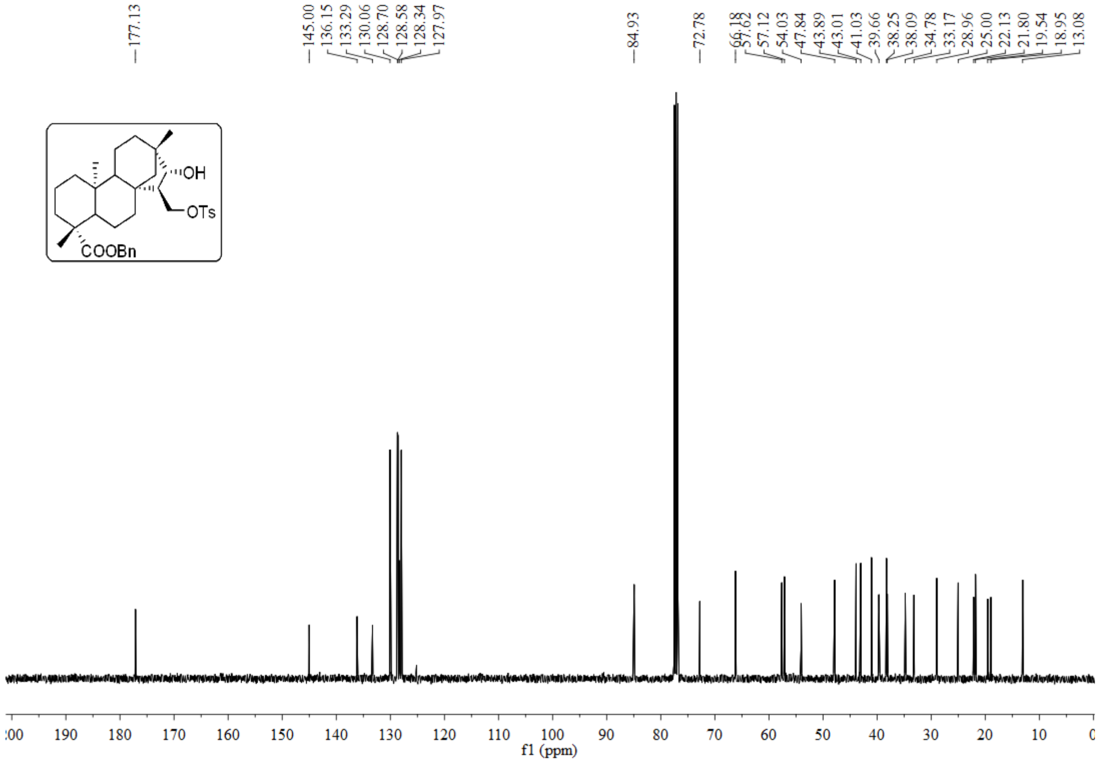

Supplementary Figure 29. ^{13}C NMR spectrum of **8**

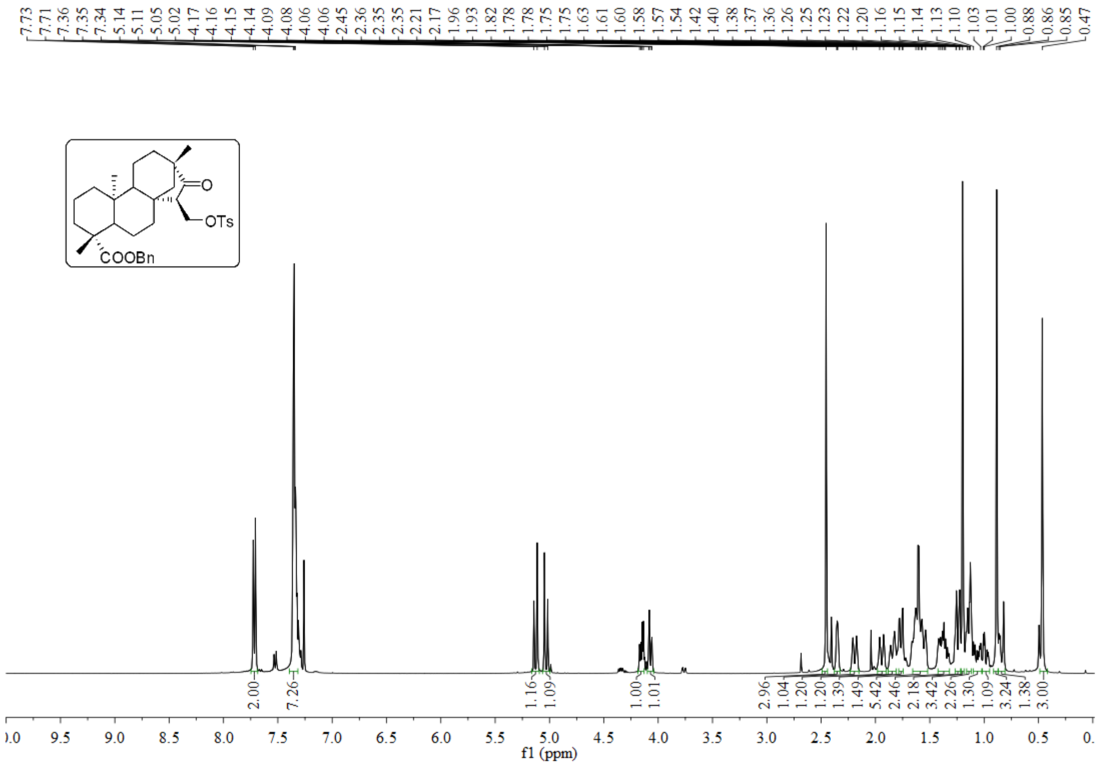

Supplementary Figure 30. ^{1}H NMR spectrum of **9**

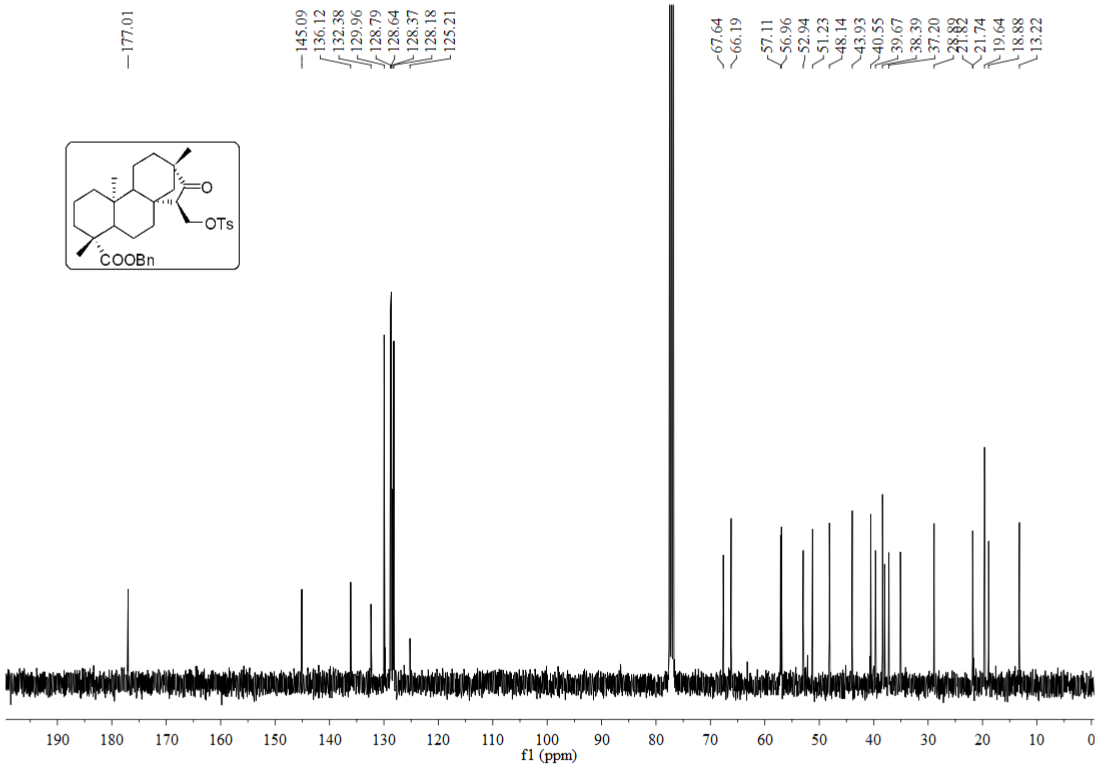

Supplementary Figure 31. ^{13}C NMR spectrum of **9**

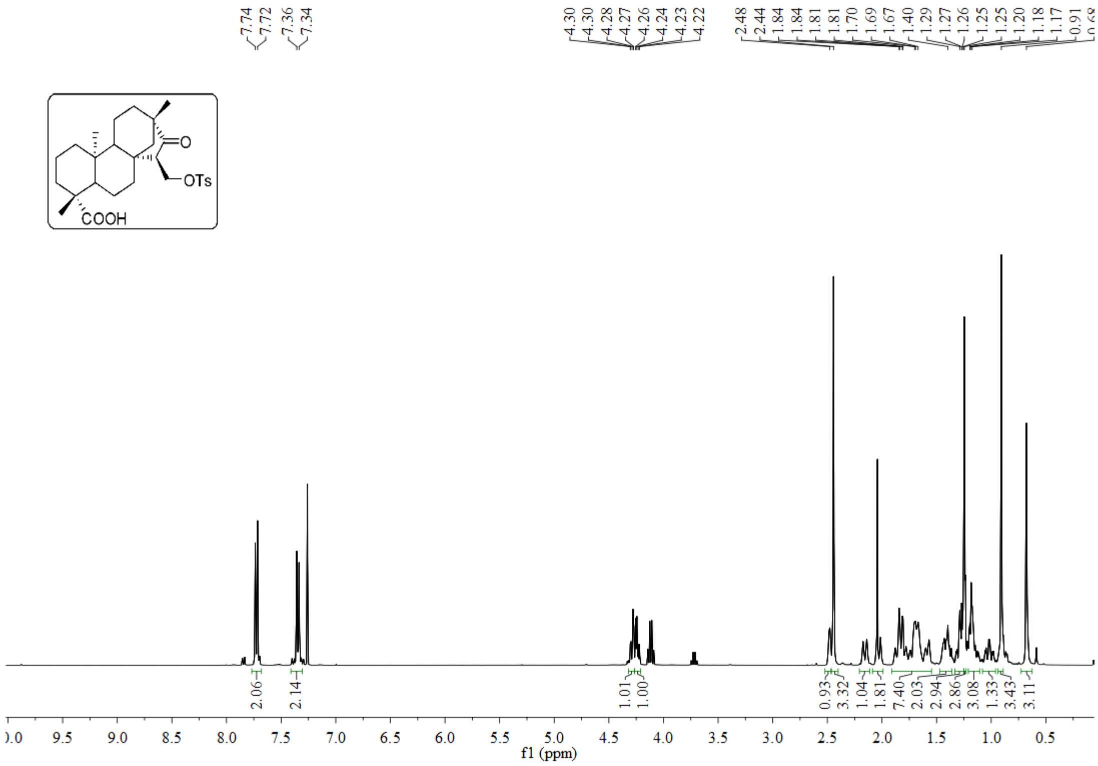

Supplementary Figure 32. ^{1}H NMR spectrum of **10**

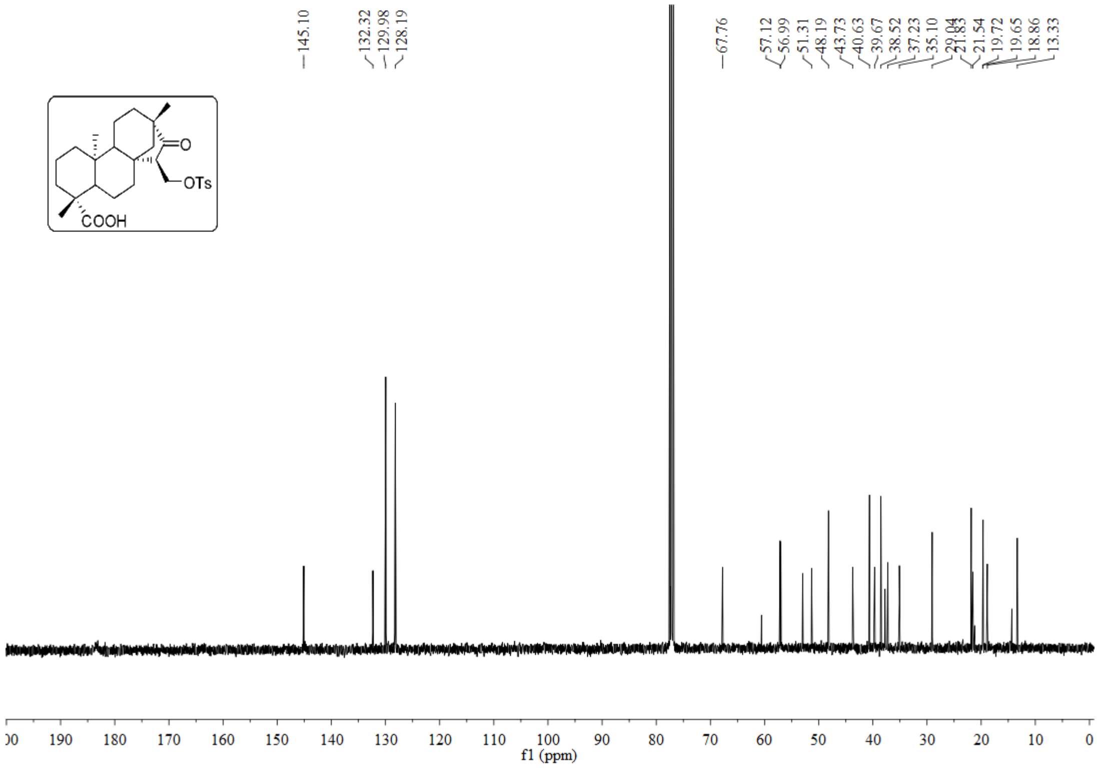

Supplementary Figure 33. ^{13}C NMR spectrum of **10**

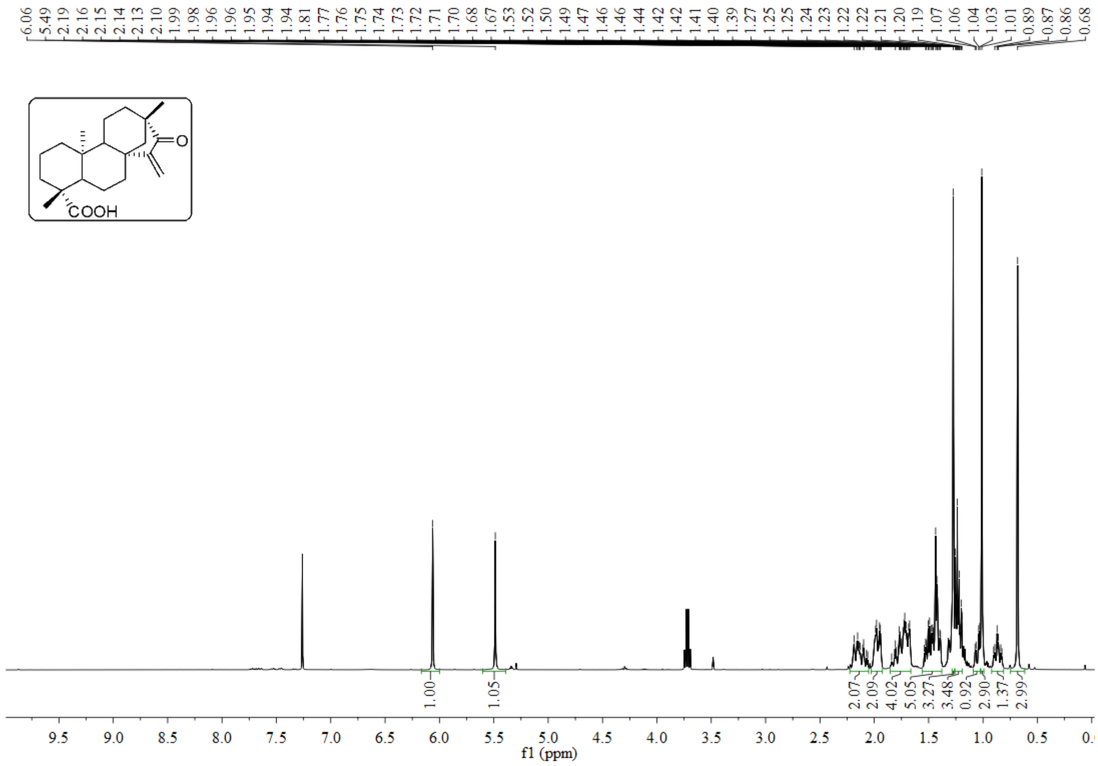

Supplementary Figure 34. ^{1}H NMR spectrum of **11**

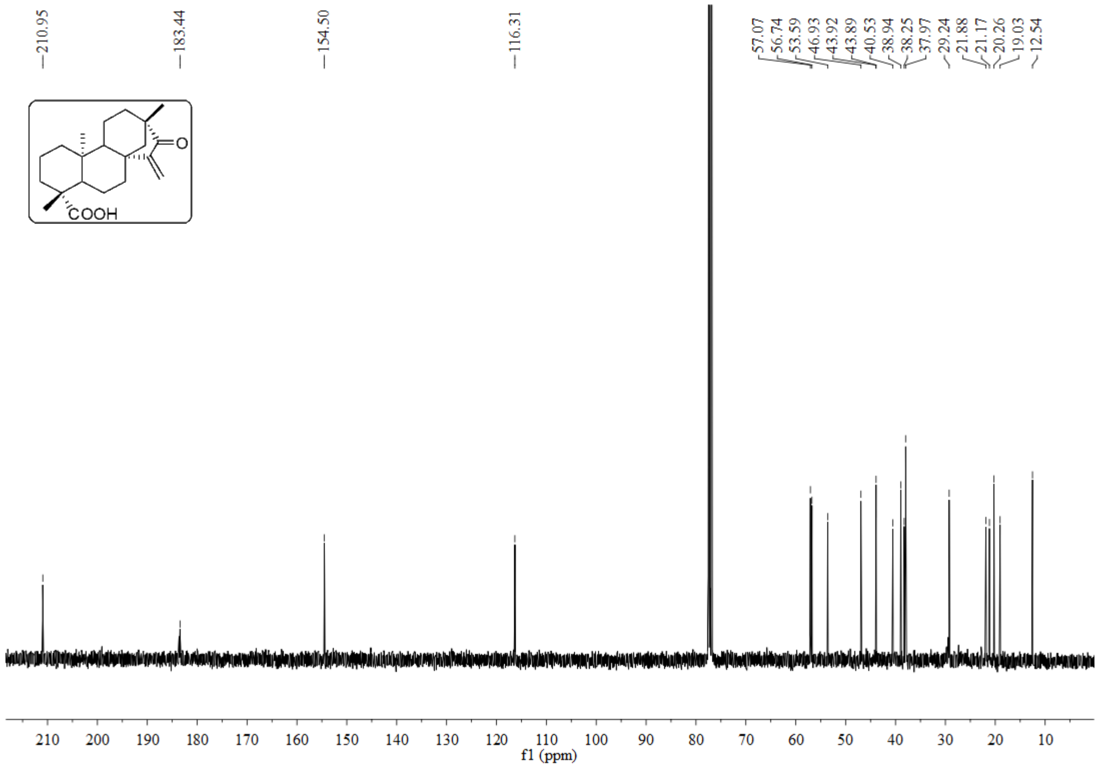

Supplementary Figure 35. ^{13}C NMR spectrum of **11**

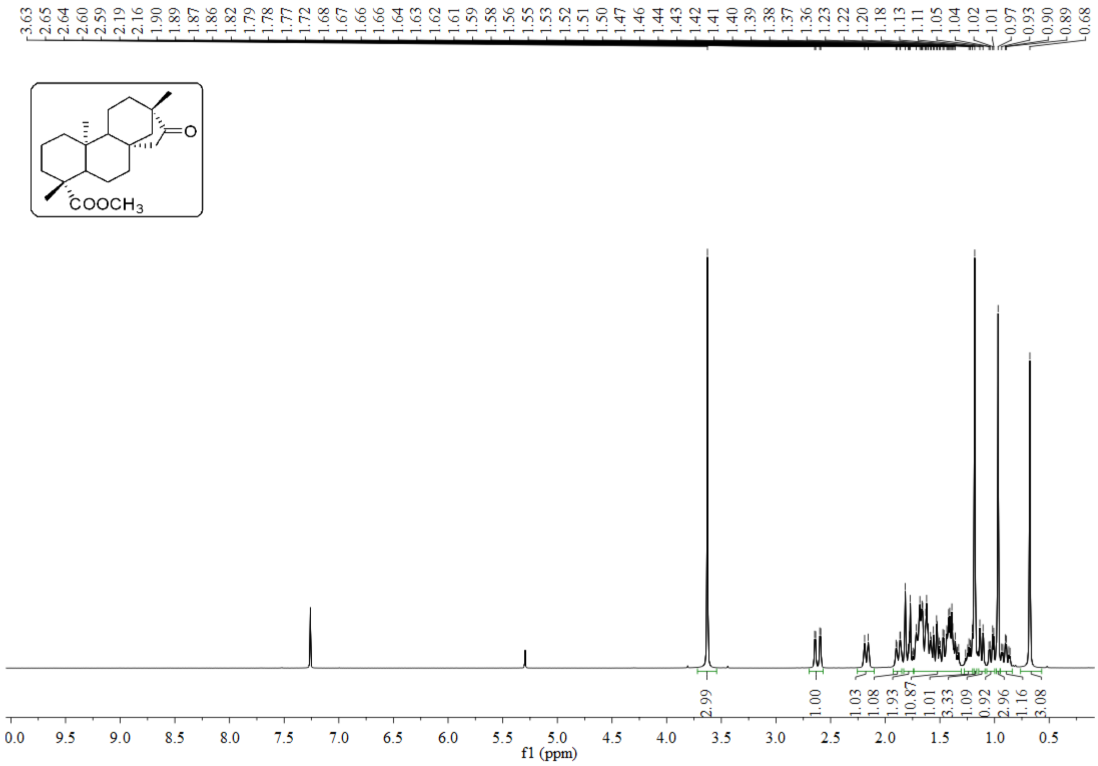

Supplementary Figure 36. ^{1}H NMR spectrum of **15**.

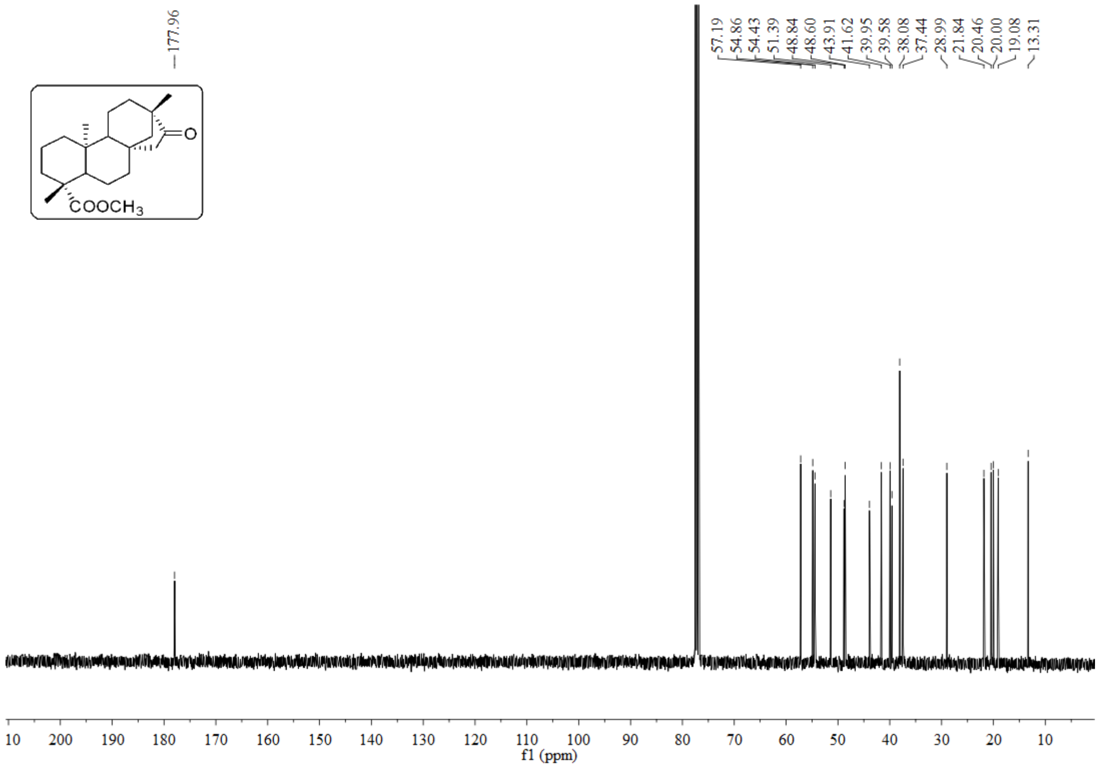

Supplementary Figure 37. ^{13}C NMR spectrum of **15**.

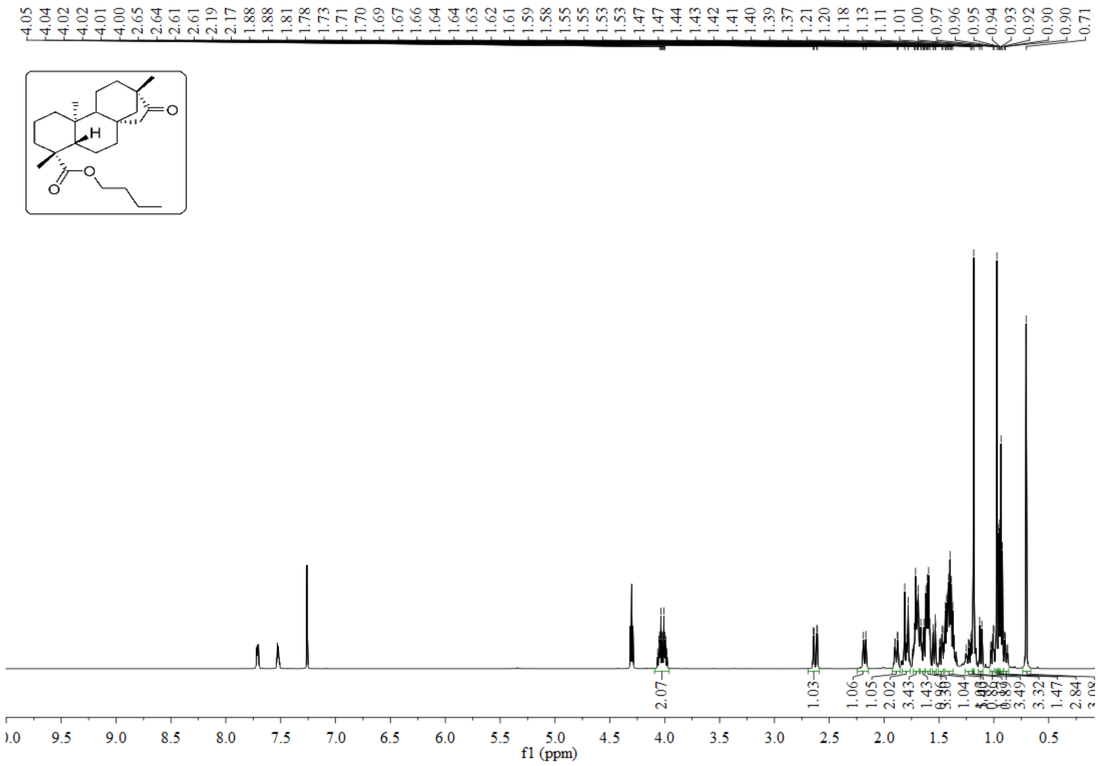

Supplementary Figure 38. ^{1}H NMR spectrum of **16**.

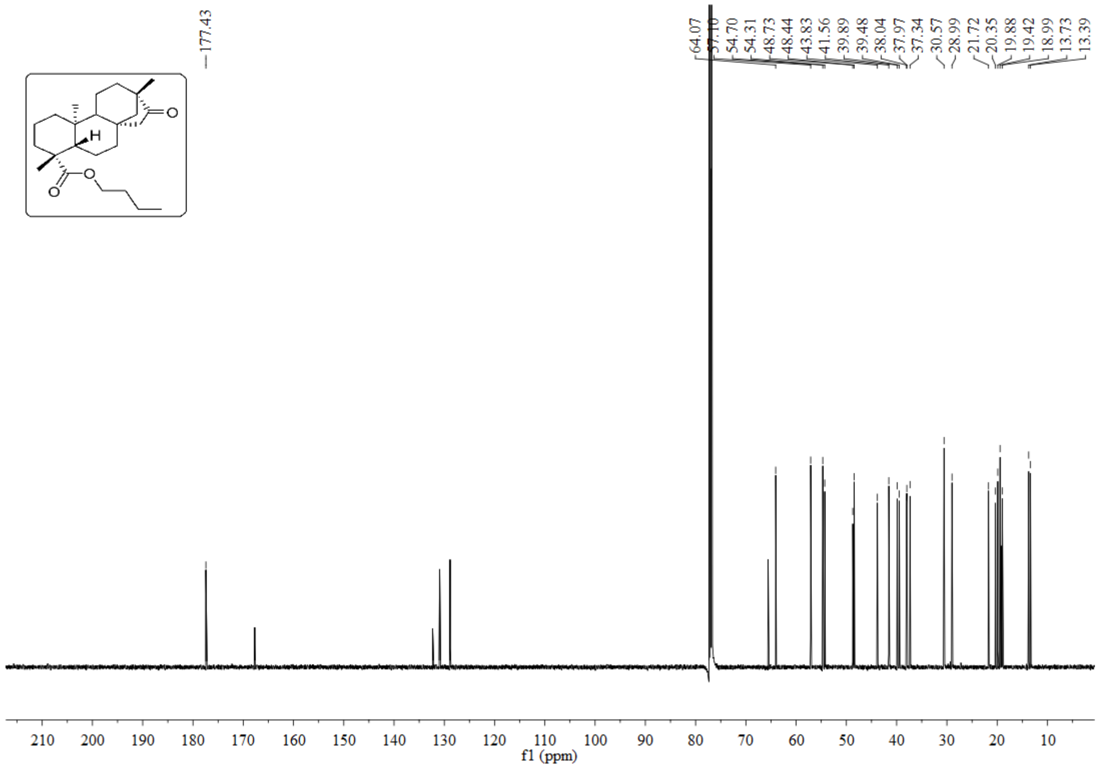

Supplementary Figure 39. ^{13}C NMR spectrum of **16**.

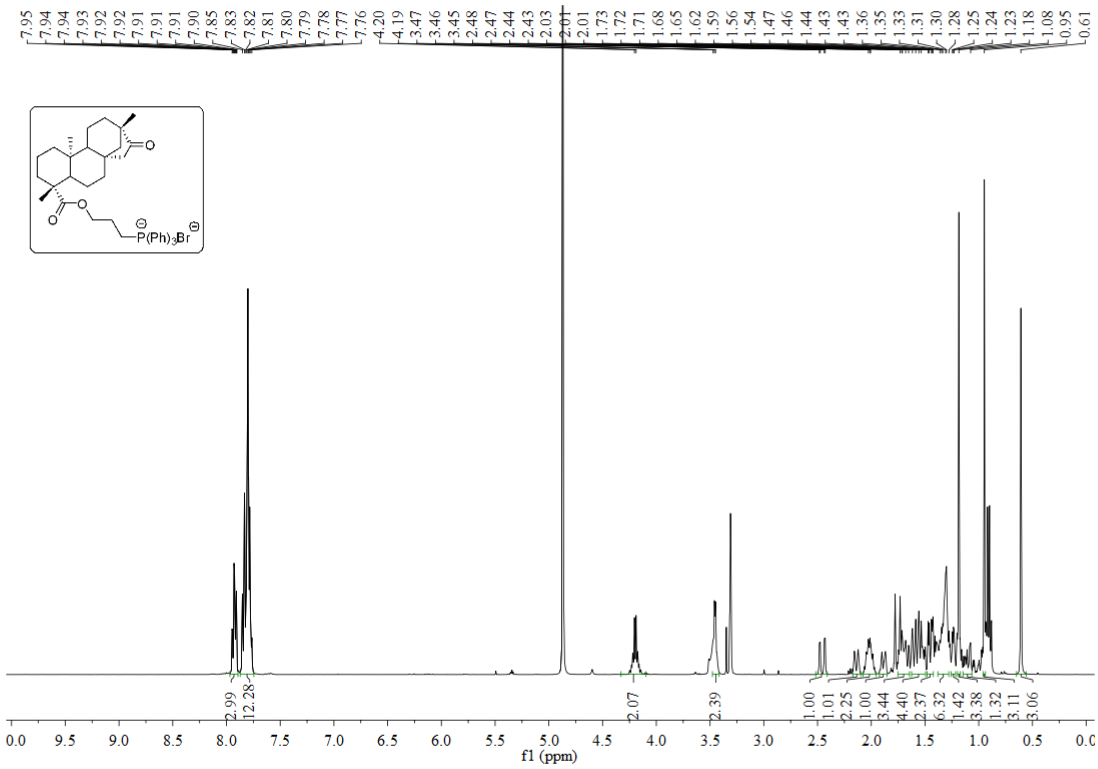

Supplementary Figure 40. ^{1}H NMR spectrum of **17**.

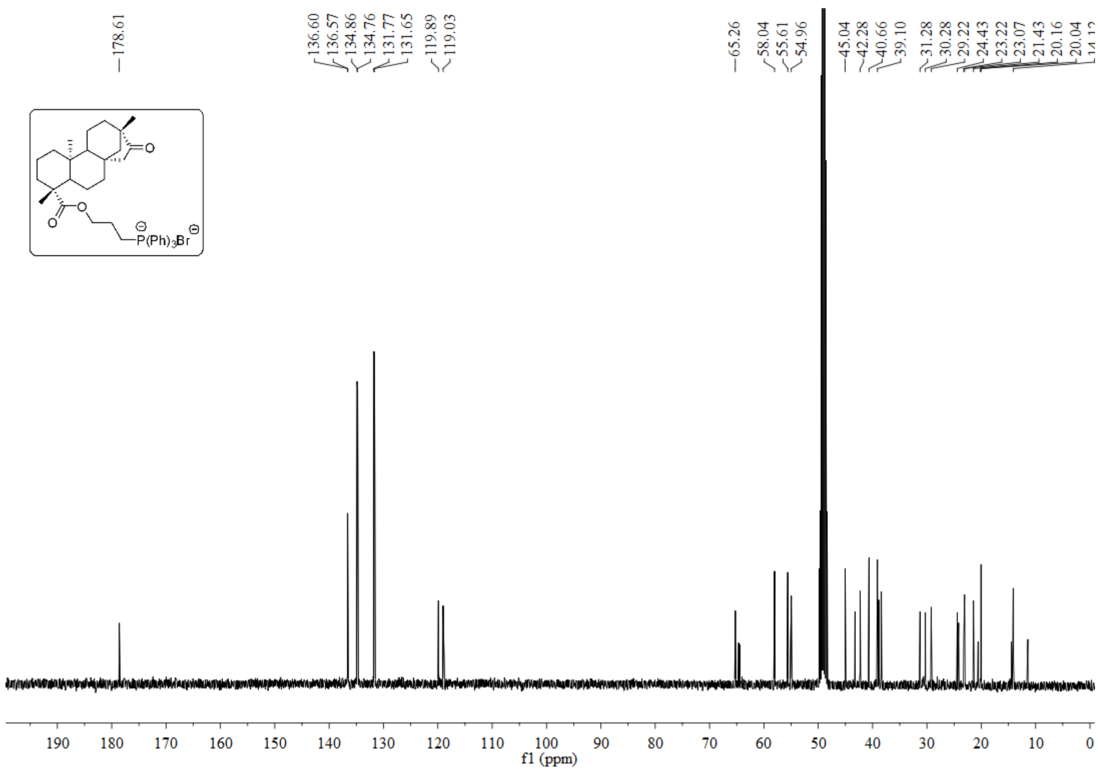

Supplementary Figure 41. ^{13}C NMR spectrum of **17**.

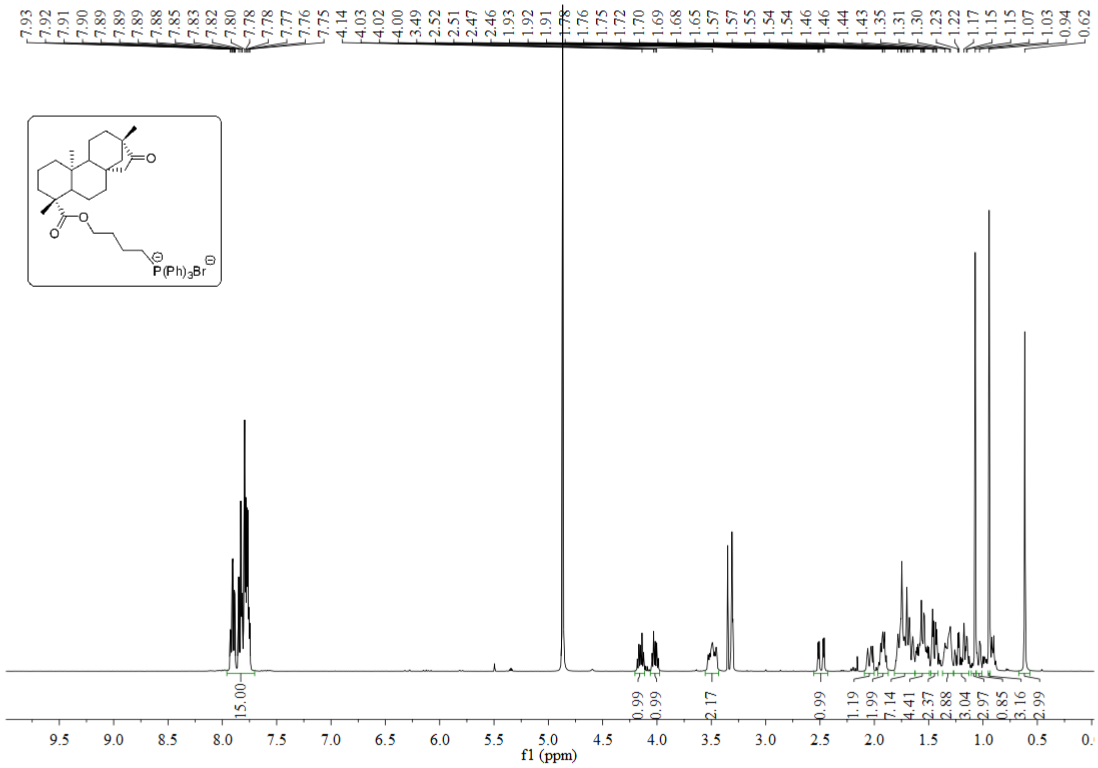

Supplementary Figure 42. ^{1}H NMR spectrum of **18**.

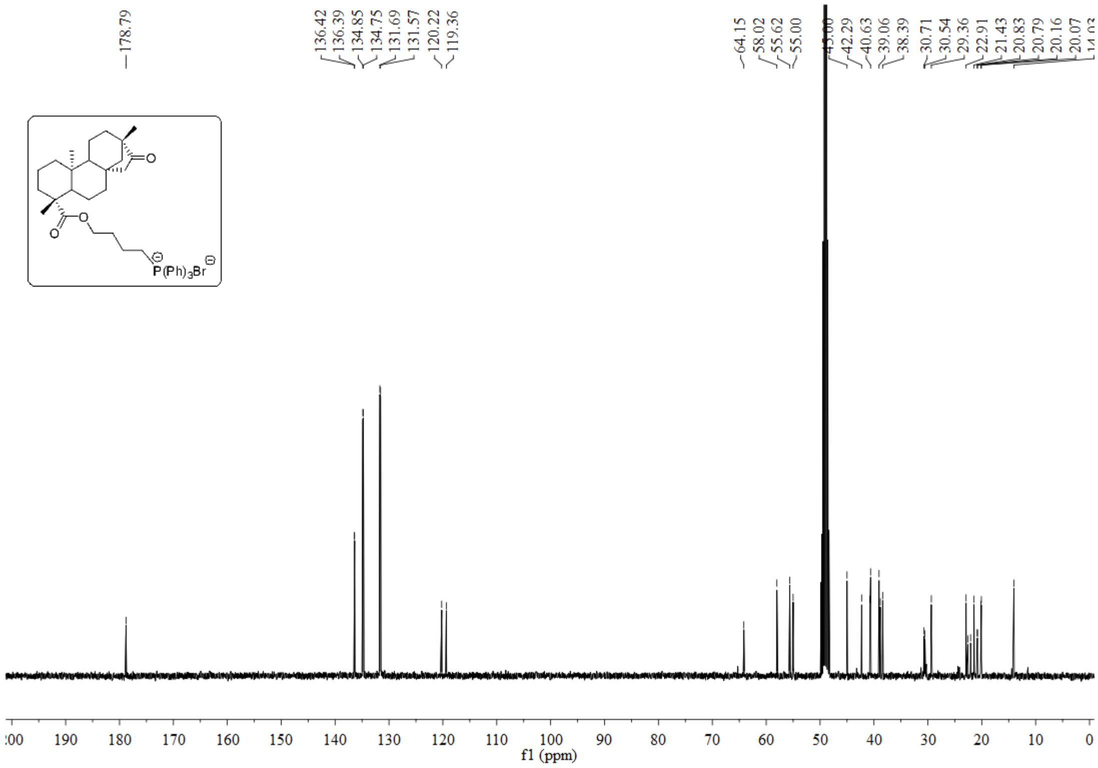

Supplementary Figure 43. ^{13}C NMR spectrum of **18**.

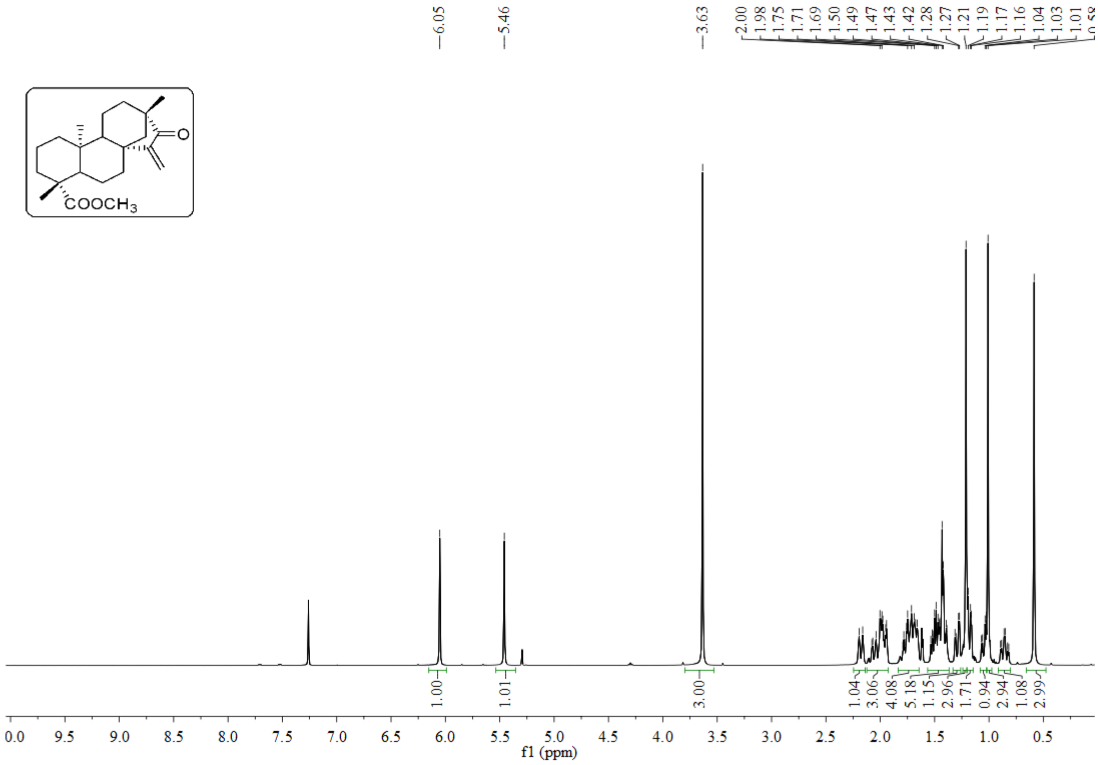

Supplementary Figure 44. ^{1}H NMR spectrum of **19**.

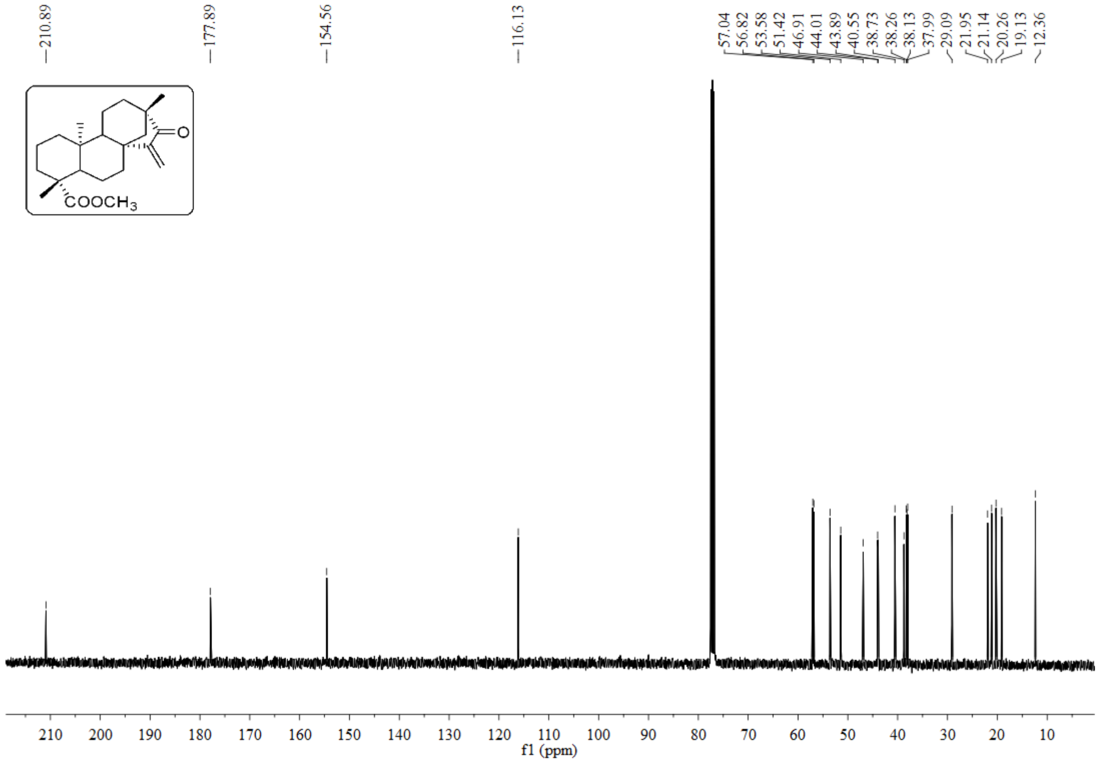

Supplementary Figure 45. ^{13}C NMR spectrum of **19**.

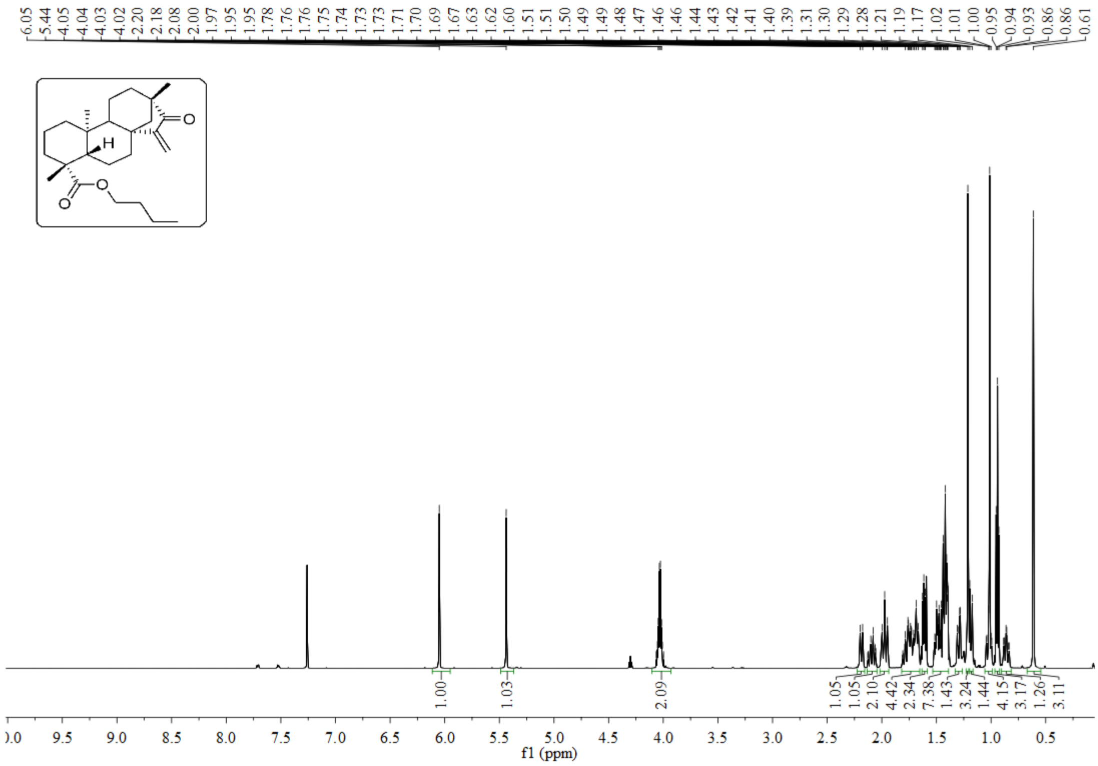

Supplementary Figure 46. ^{1}H NMR spectrum of **20**.

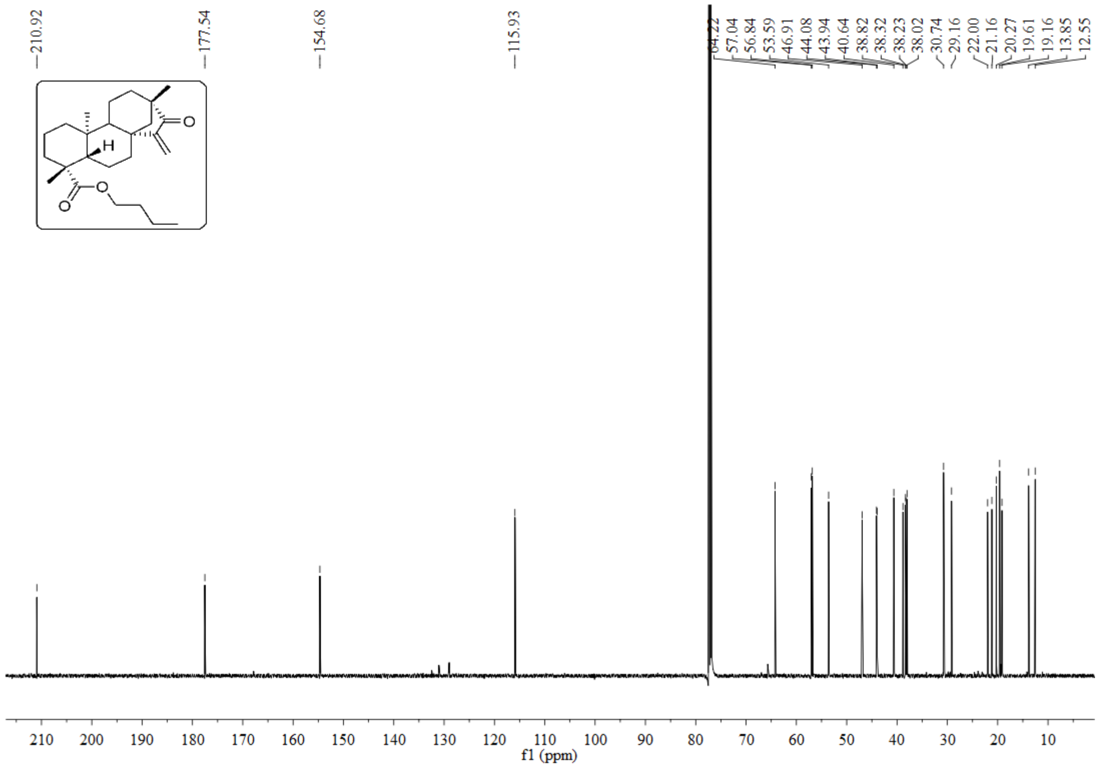

Supplementary Figure 47. ^{13}C NMR spectrum of **20**.

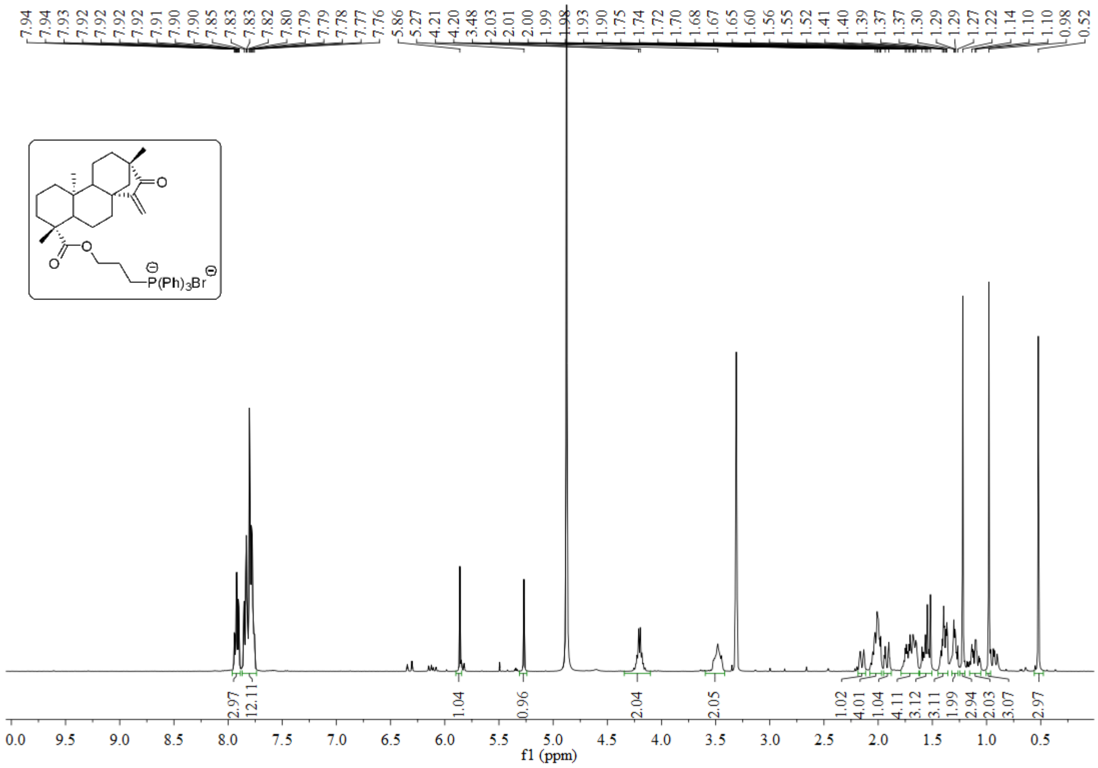

Supplementary Figure 48. ^{1}H NMR spectrum of **21**.

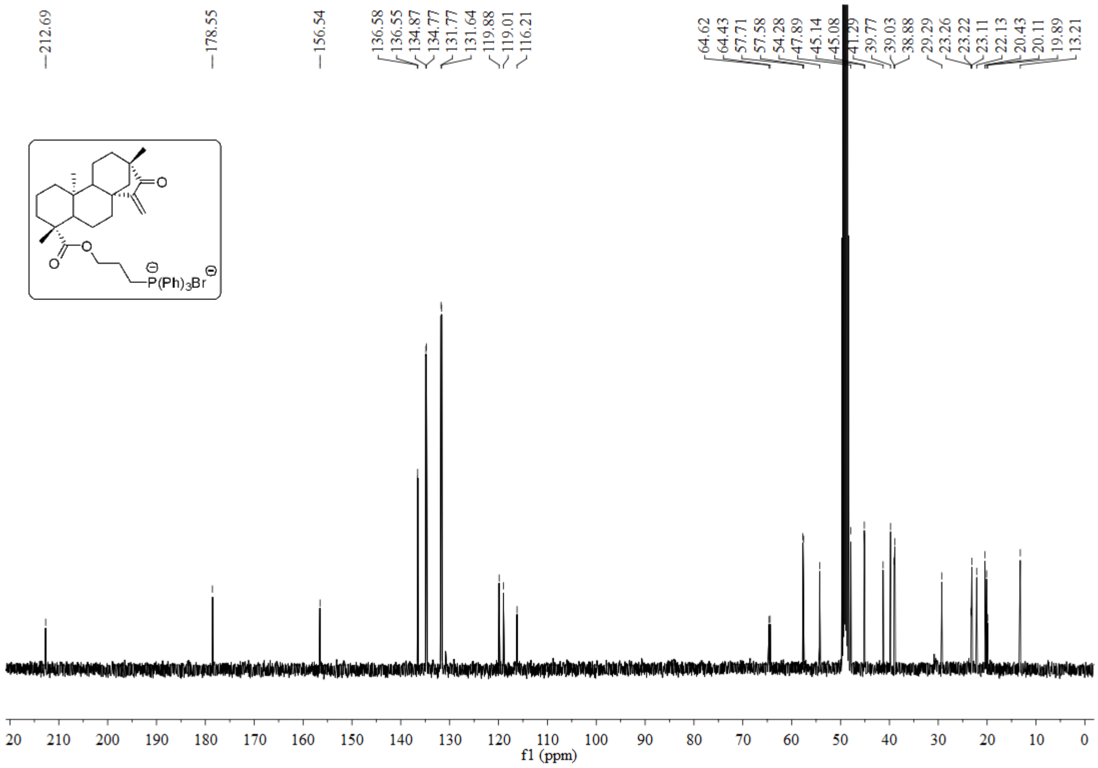

Supplementary Figure 49. ^{13}C NMR spectrum of **21**.

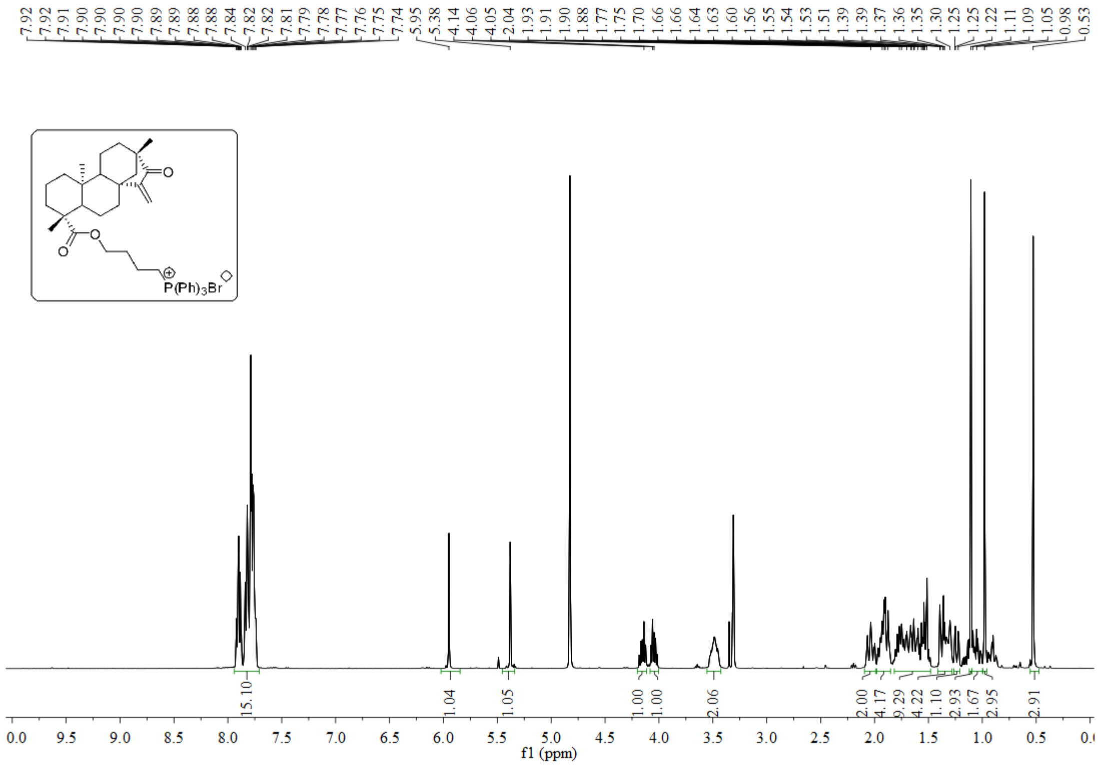

Supplementary Figure 50. ^{1}H NMR spectrum of **22**.

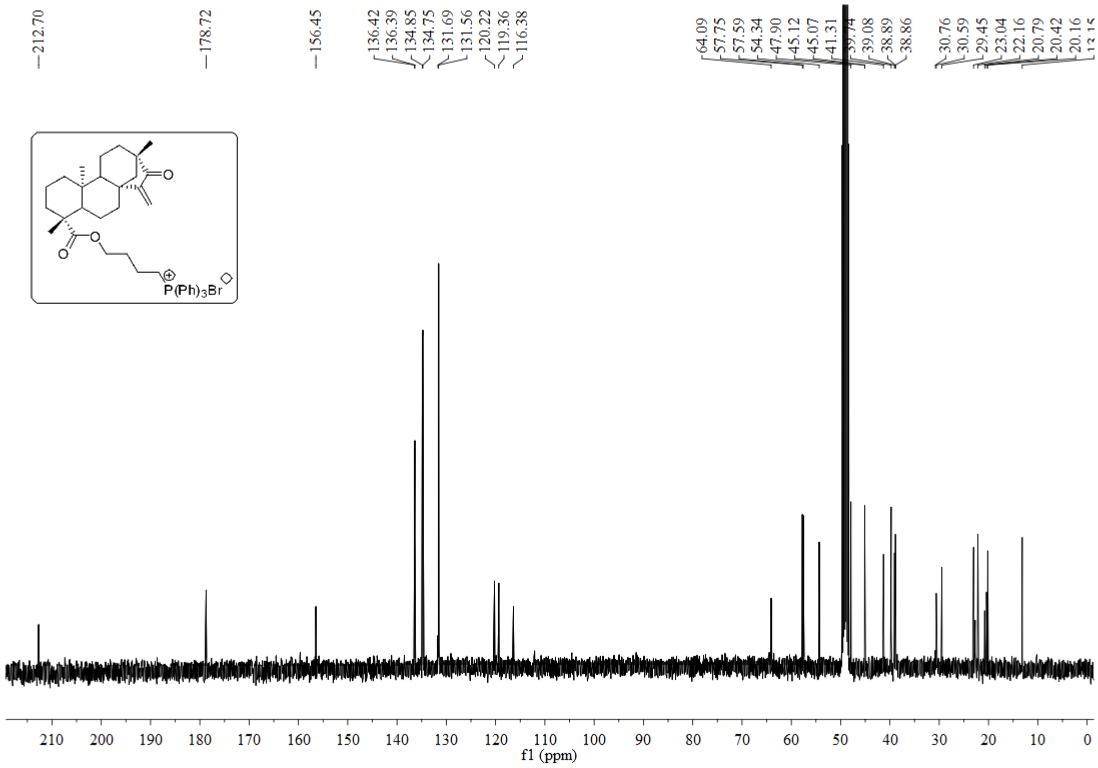

Supplementary Figure 51. ^{13}C NMR spectrum of **22**.

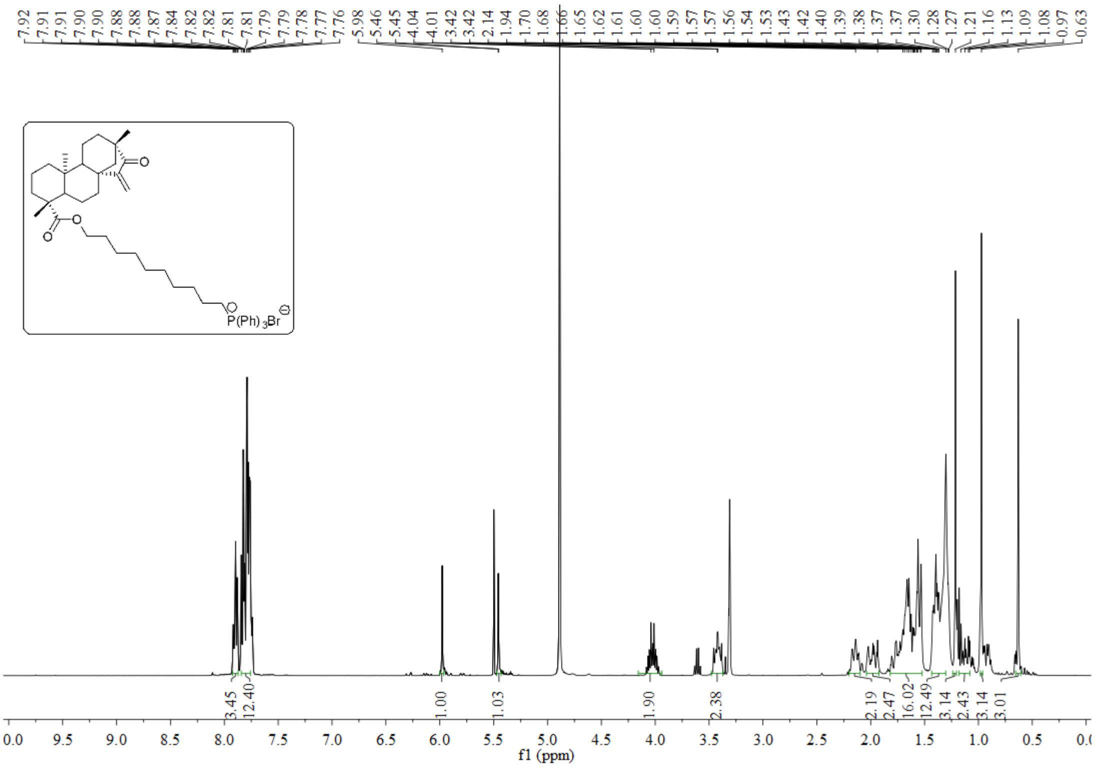

Supplementary Figure 52. ^{1}H NMR spectrum of **23**.

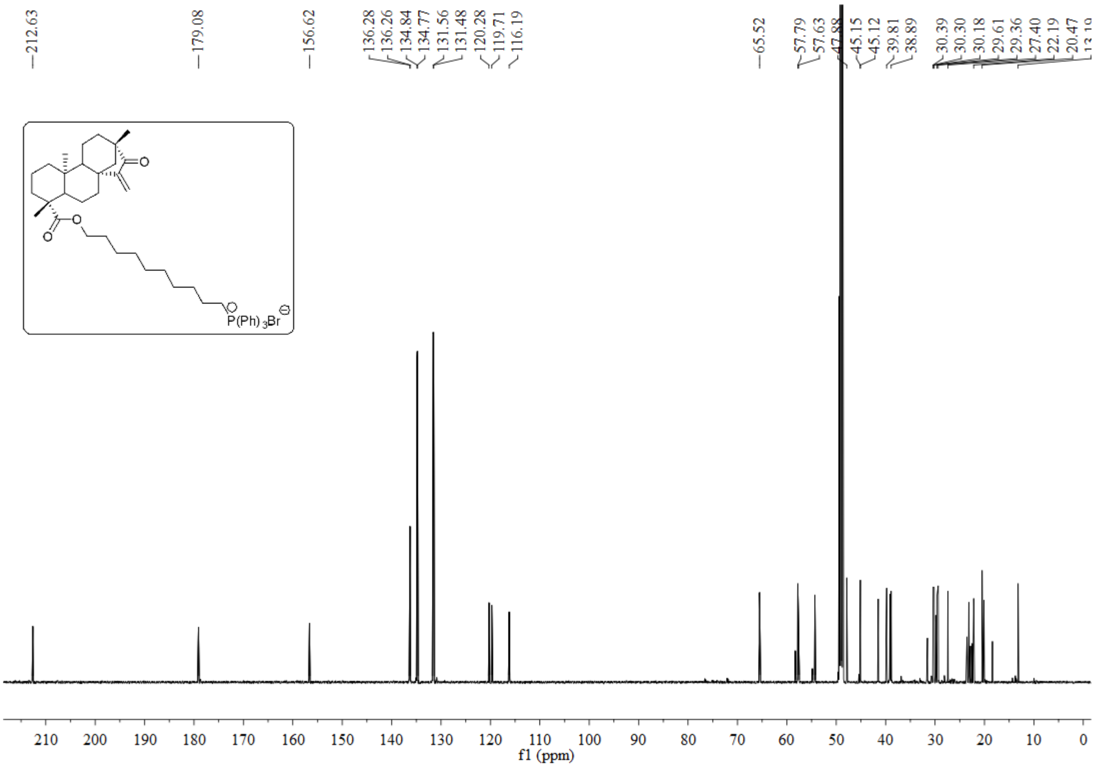

Supplementary Figure 53. ^{13}C NMR spectrum of **23**.

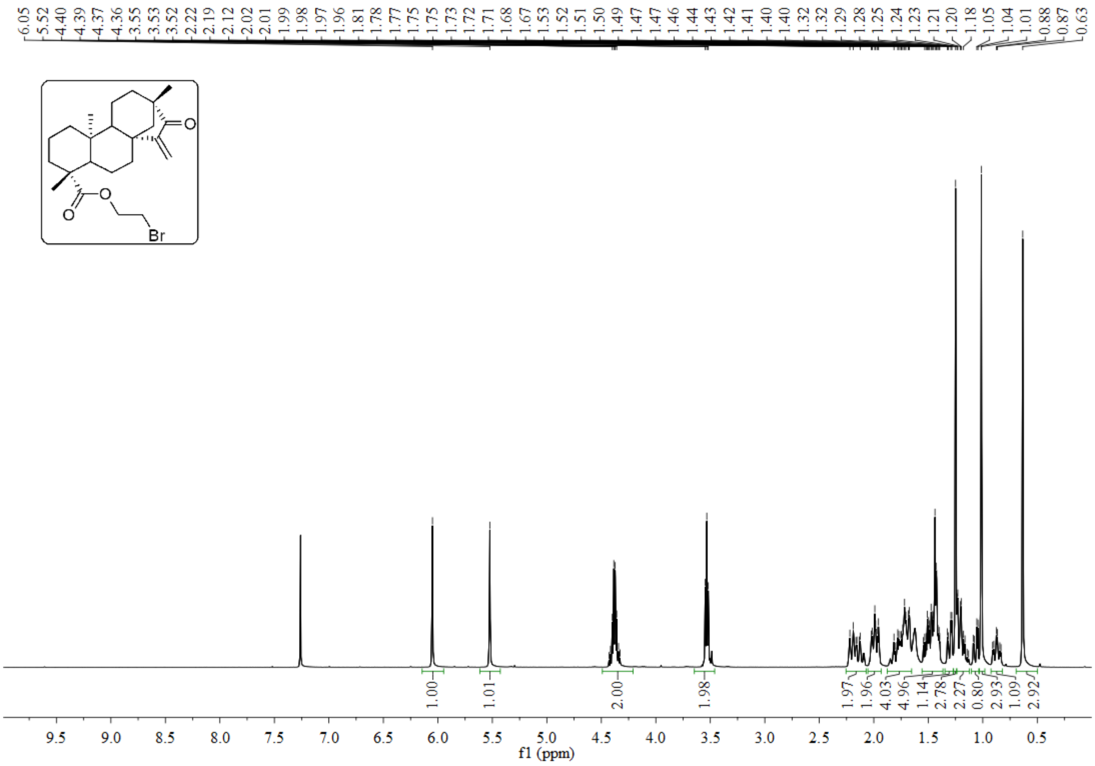

Supplementary Figure 54. ^{1}H NMR spectrum of **24**.

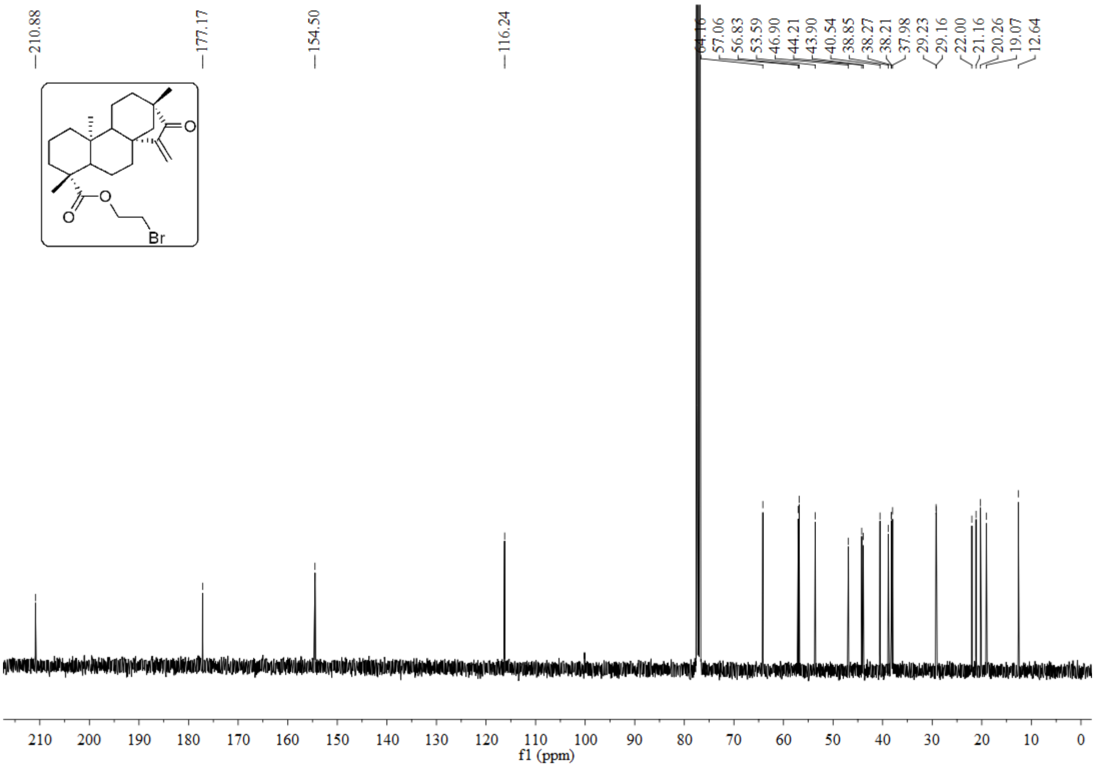

Supplementary Figure 55. ^{13}C NMR spectrum of **24**.

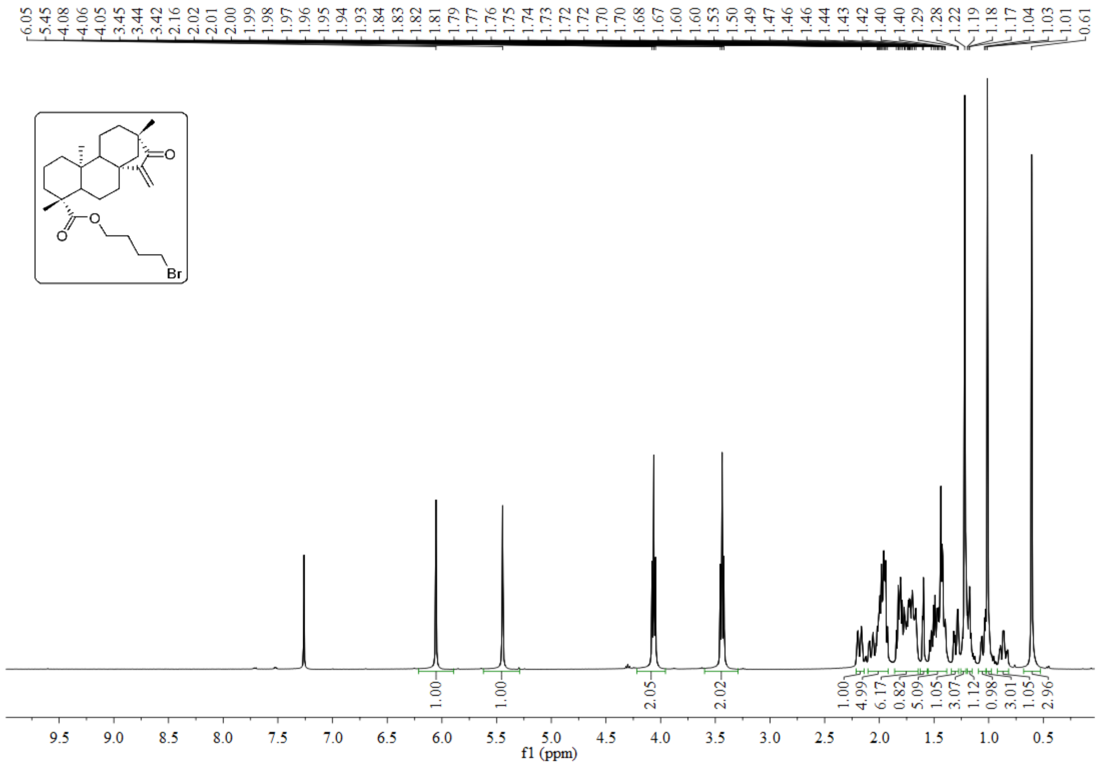

Supplementary Figure 56. ^{1}H NMR spectrum of **25**.

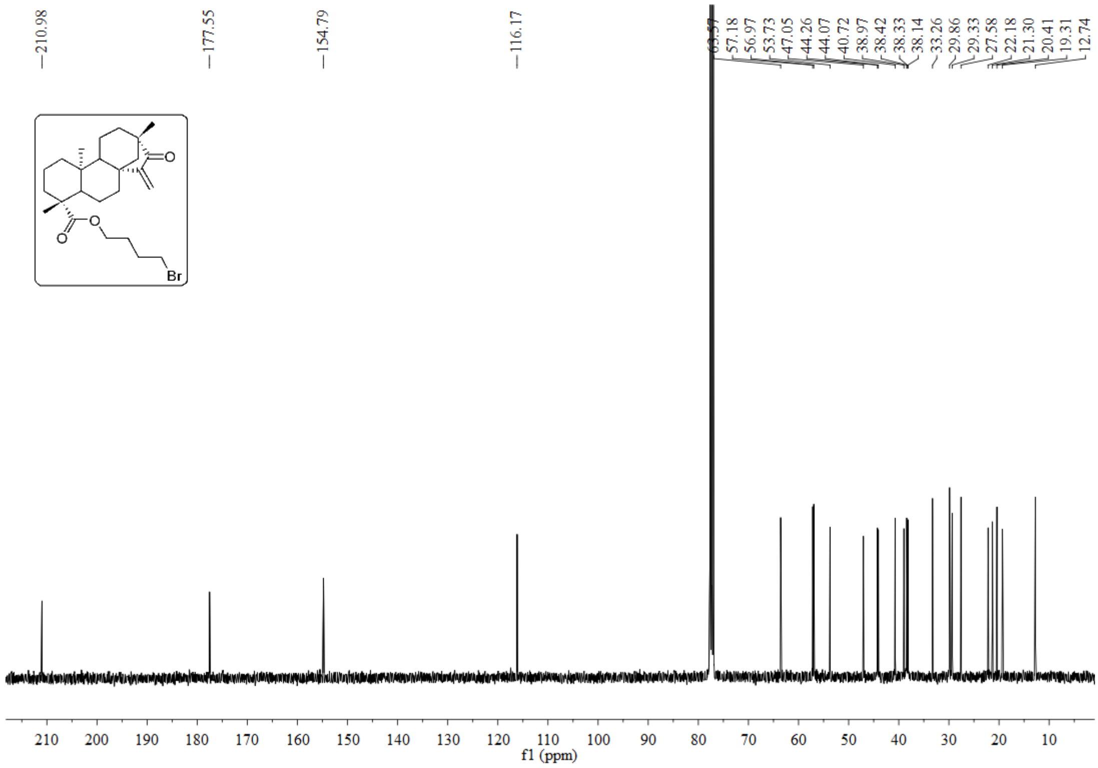

Supplementary Figure 57. ^{13}C NMR spectrum of **25**.

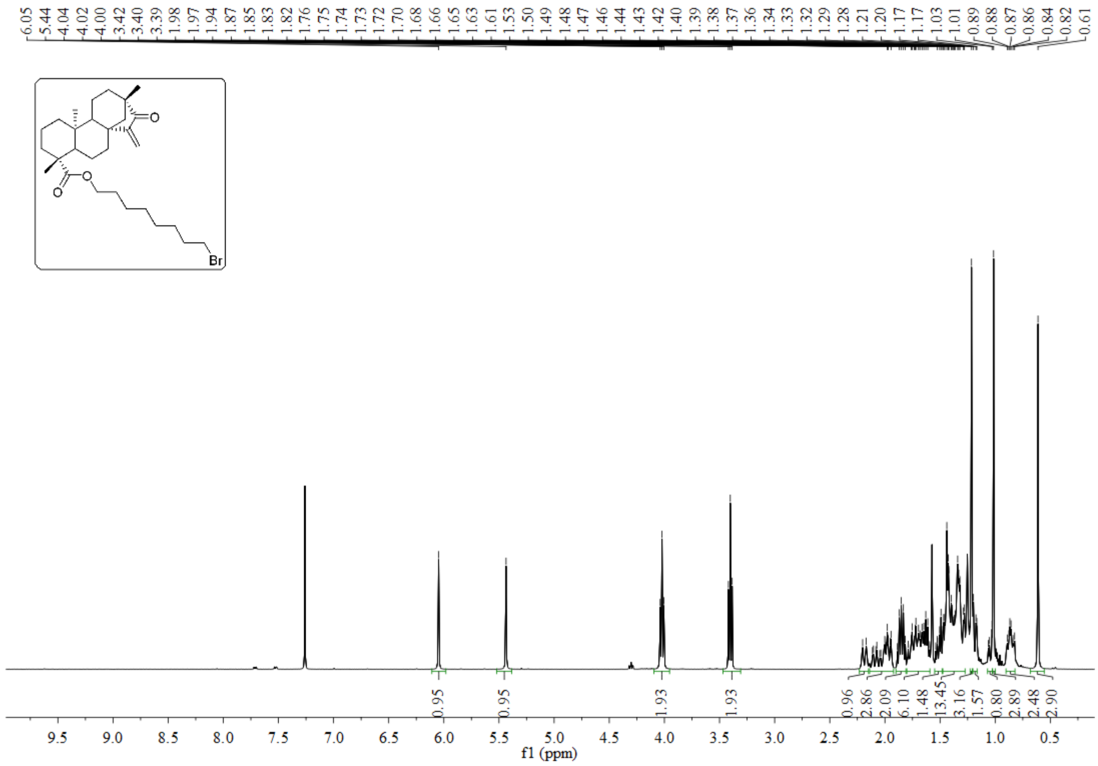

Supplementary Figure 58. ^{1}H NMR spectrum of **26**.

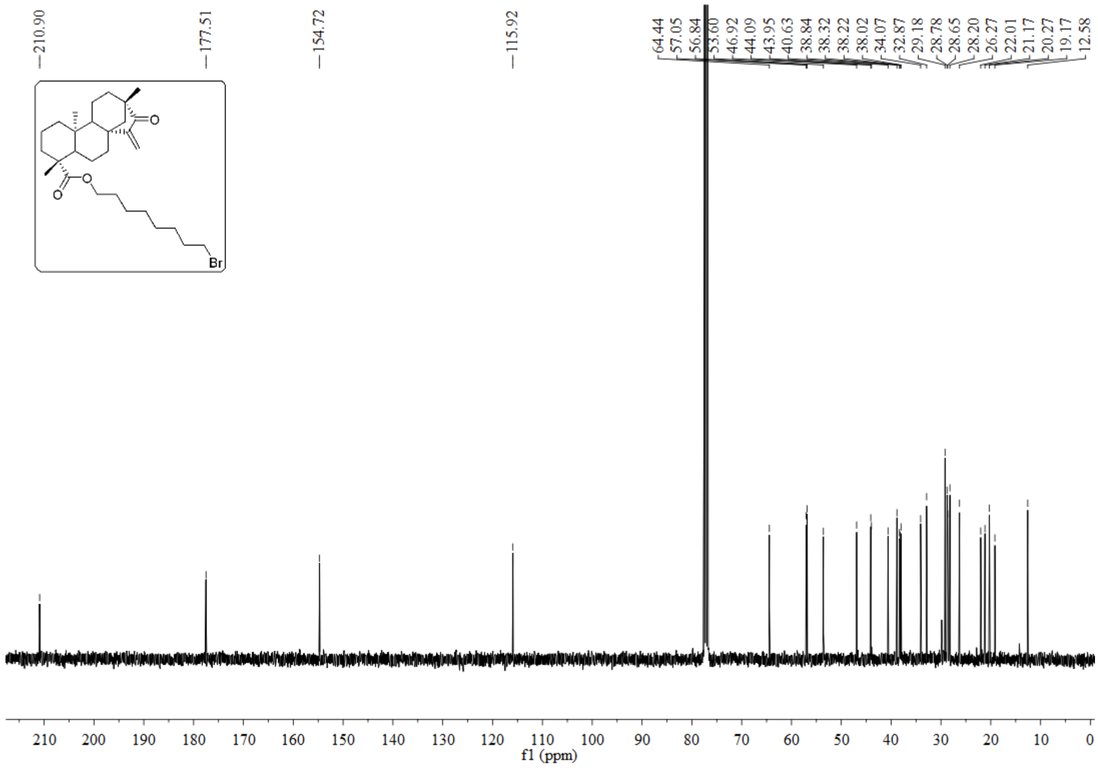

Supplementary Figure 59. ^{13}C NMR spectrum of **26**.

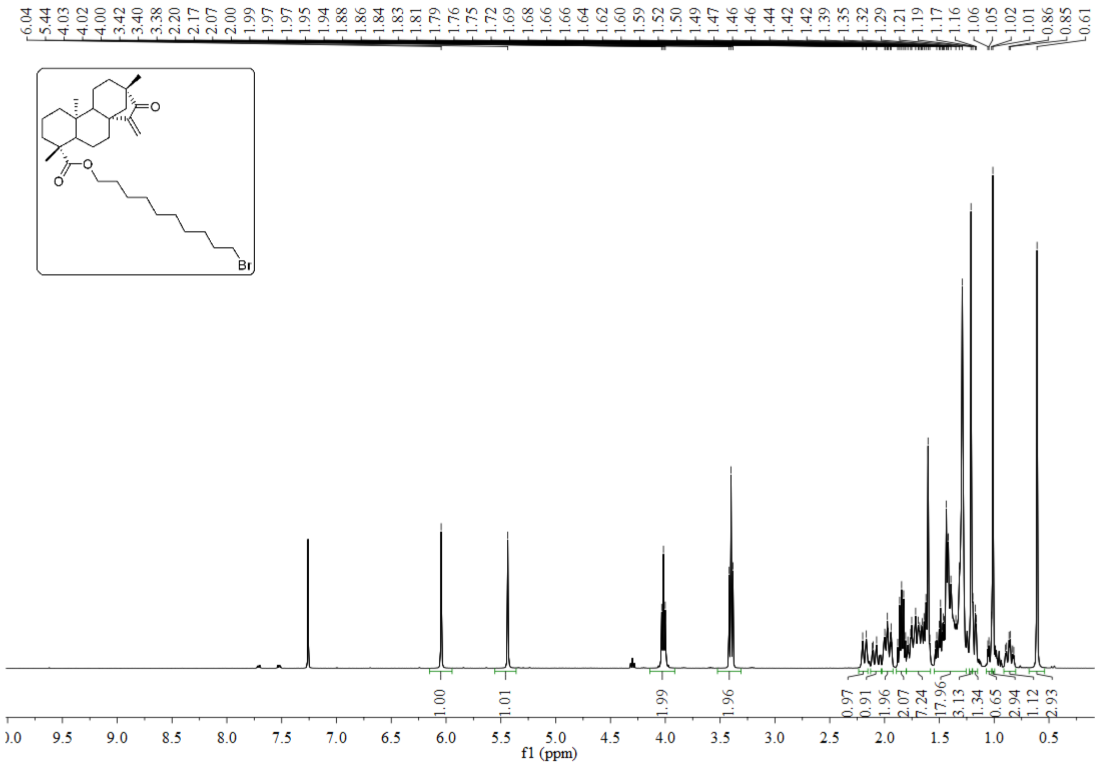

Supplementary Figure 60. ^{1}H NMR spectrum of **27**.

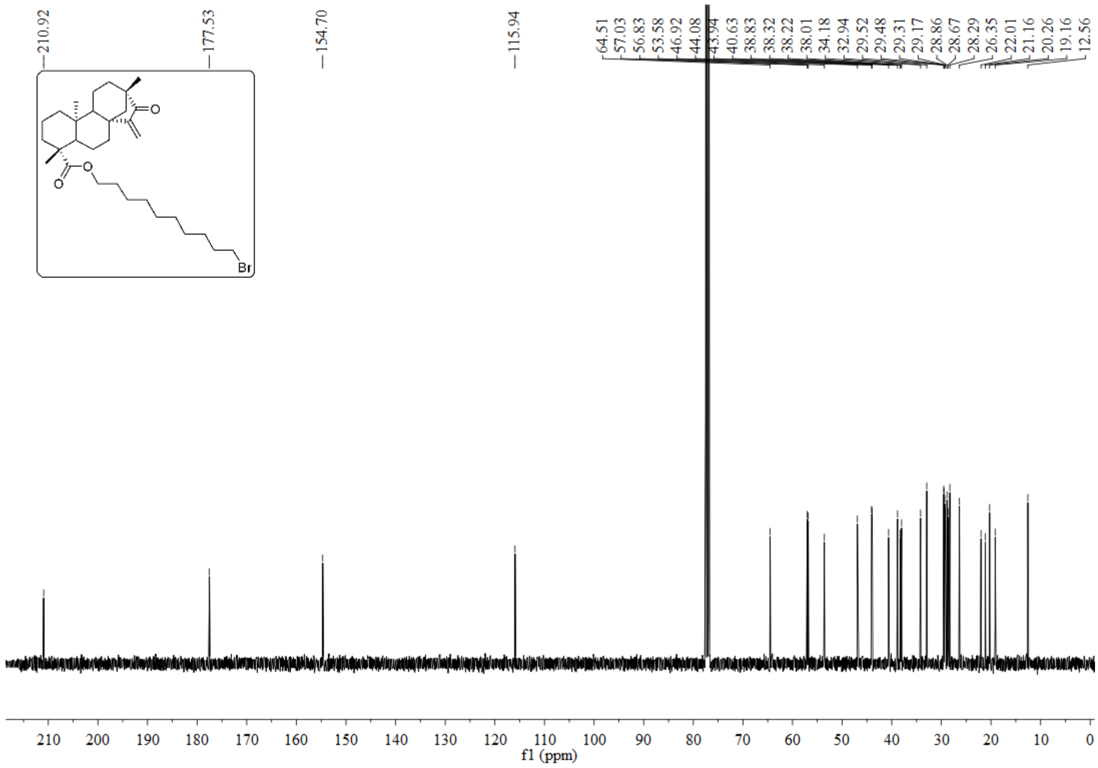

Supplementary Figure 61. ^{13}C NMR spectrum of **27**.

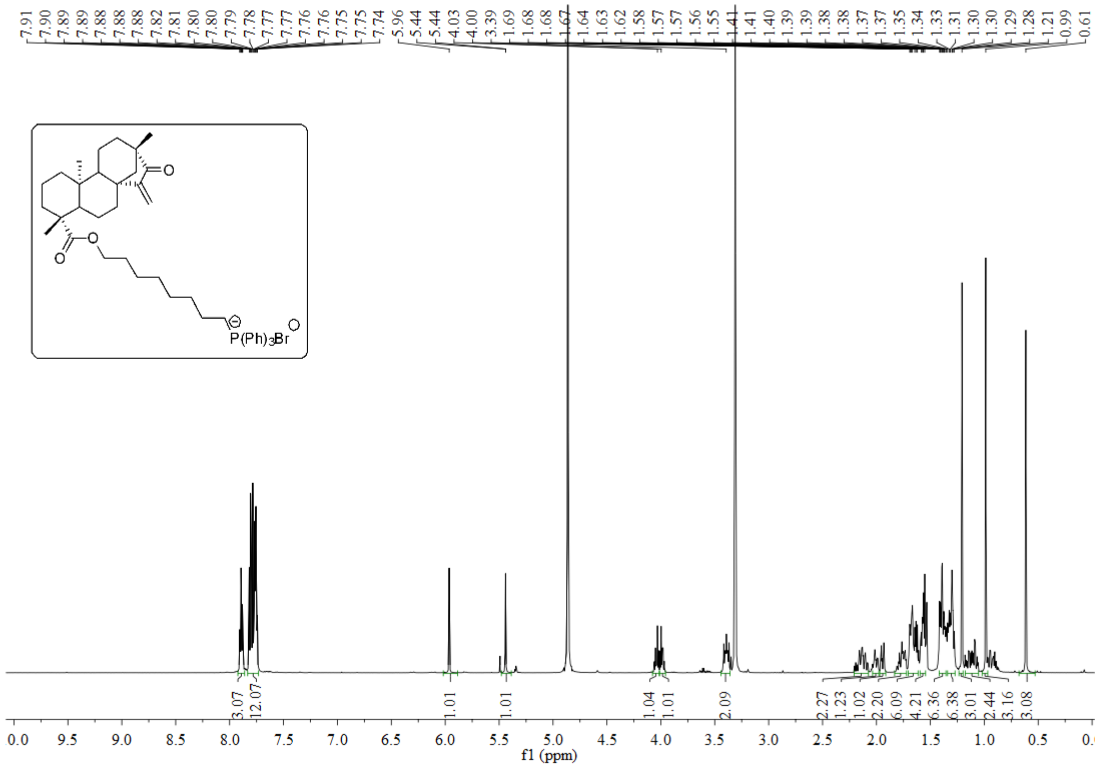

Supplementary Figure 62. ^{1}H NMR spectrum of **28**.

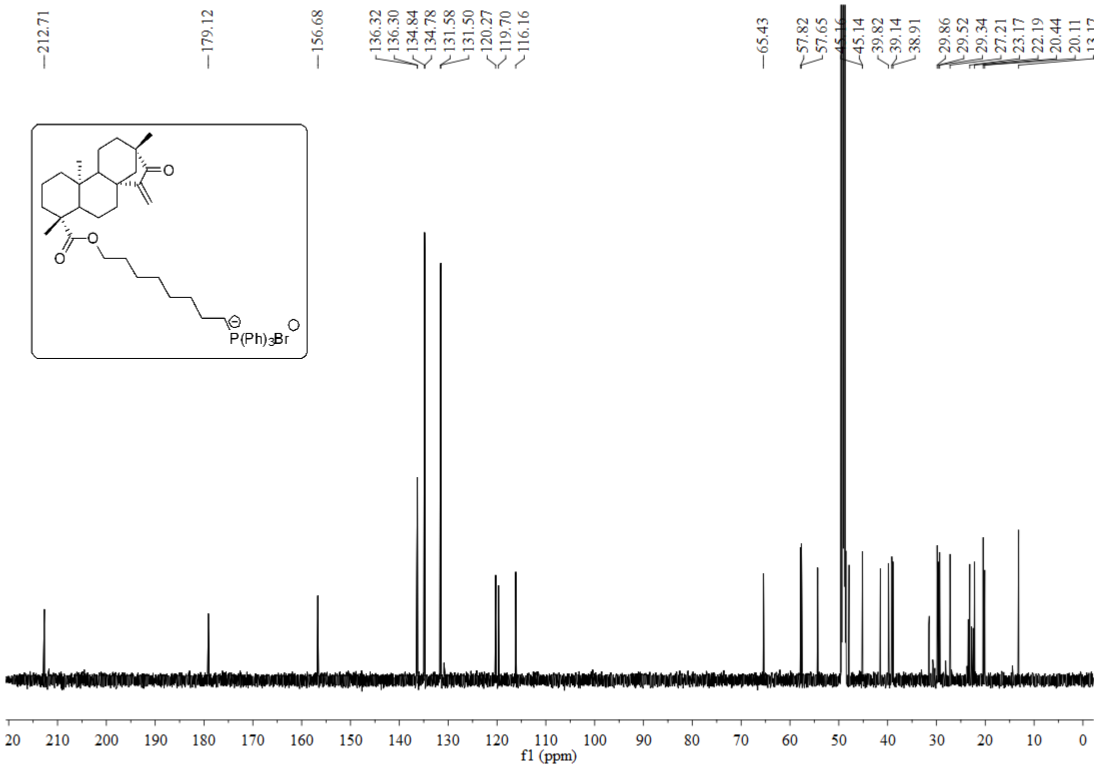

Supplementary Figure 63. ^{13}C NMR spectrum of **28**.

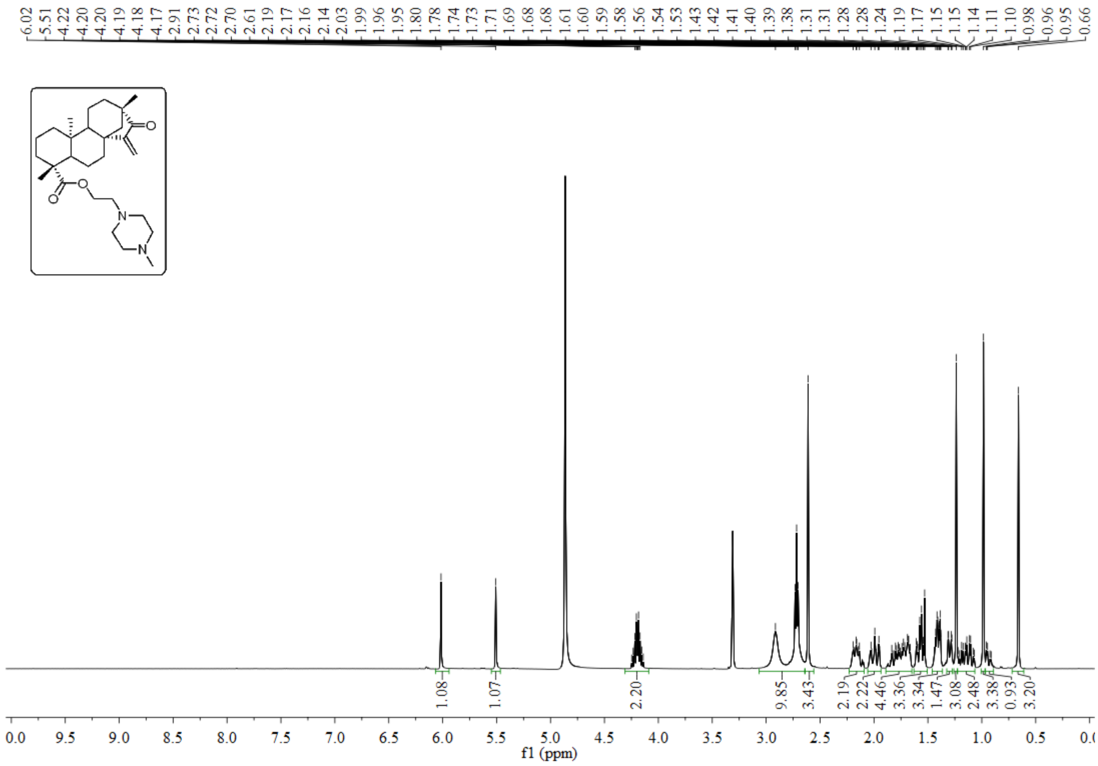

Supplementary Figure 64. ^{1}H NMR spectrum of **29**.

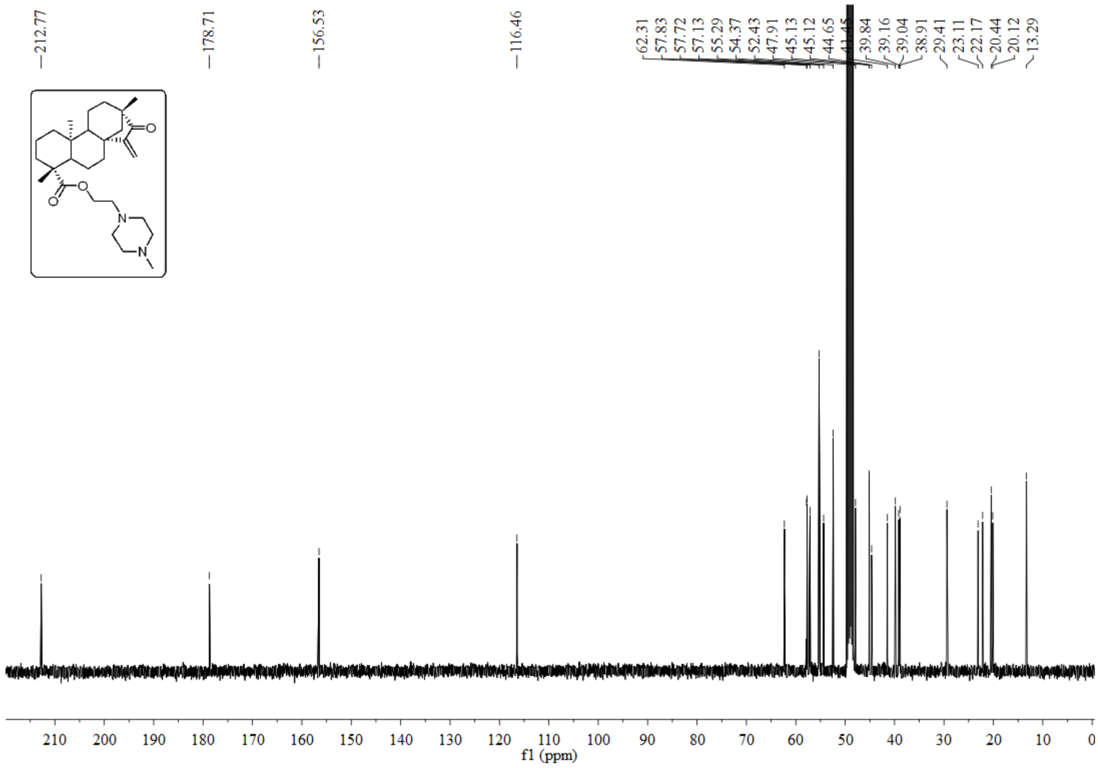

Supplementary Figure 65. ^{13}C NMR spectrum of **29**.

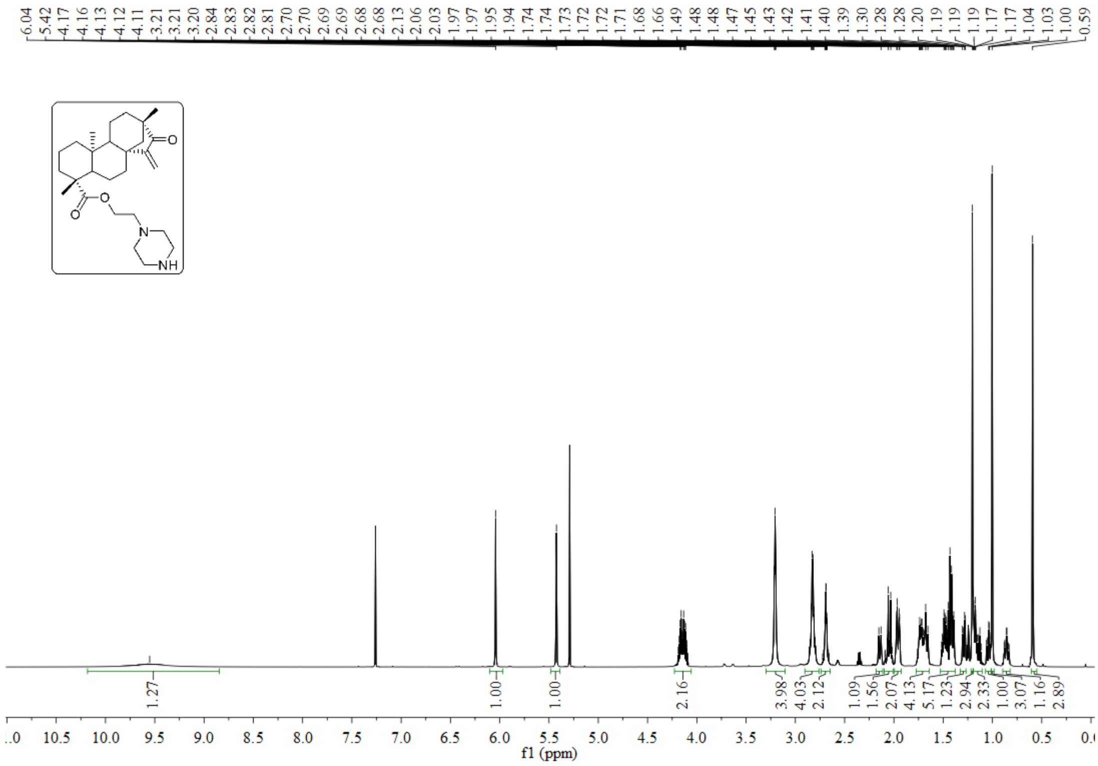

Supplementary Figure 66. ^{1}H NMR spectrum of **30**.

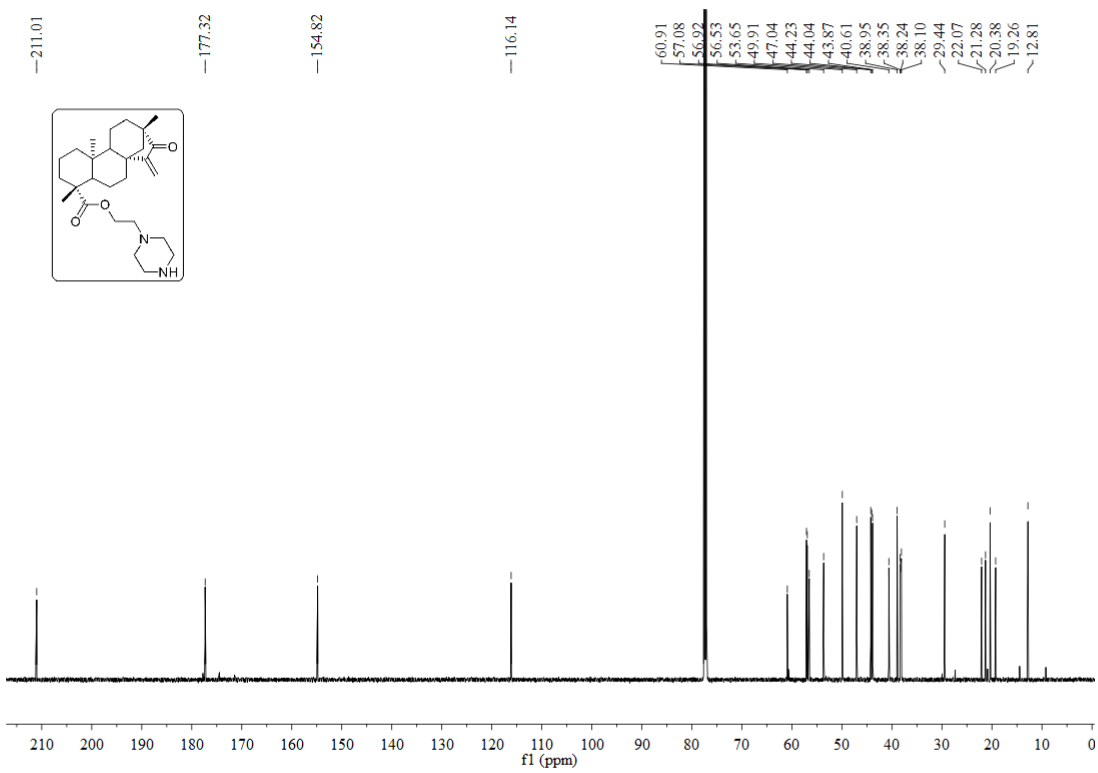

Supplementary Figure 67. ^{13}C NMR spectrum of **30**.

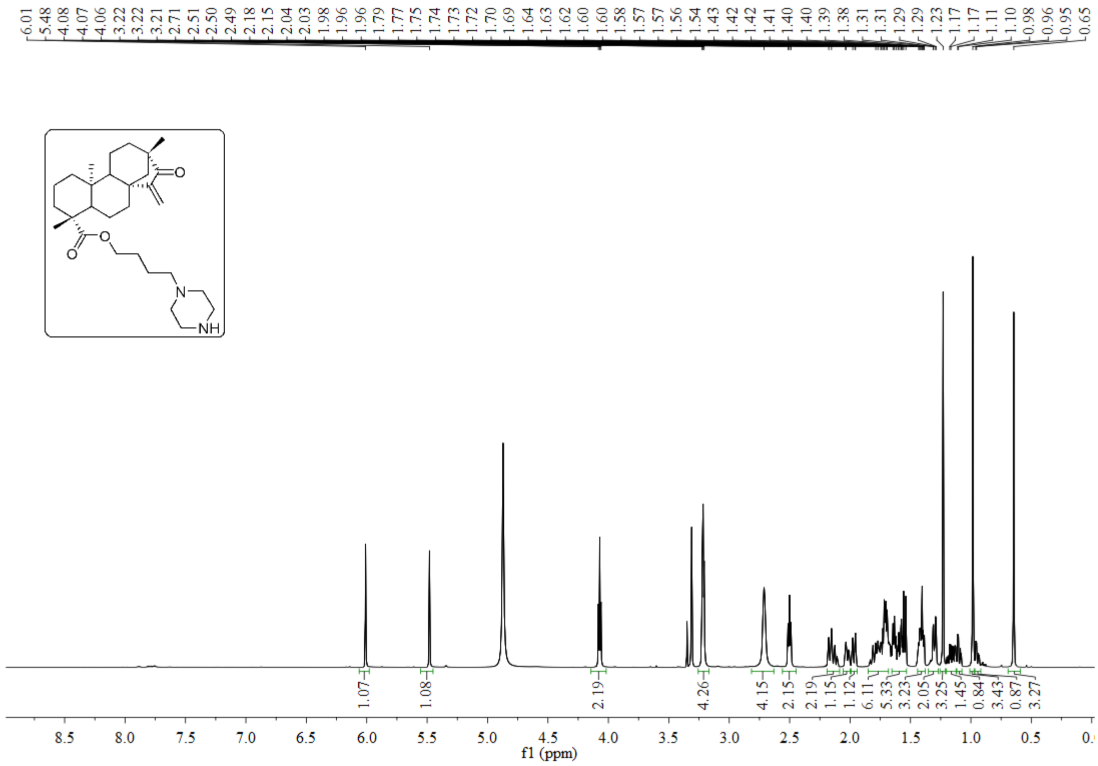

Supplementary Figure 68. ^{1}H NMR spectrum of **31**.

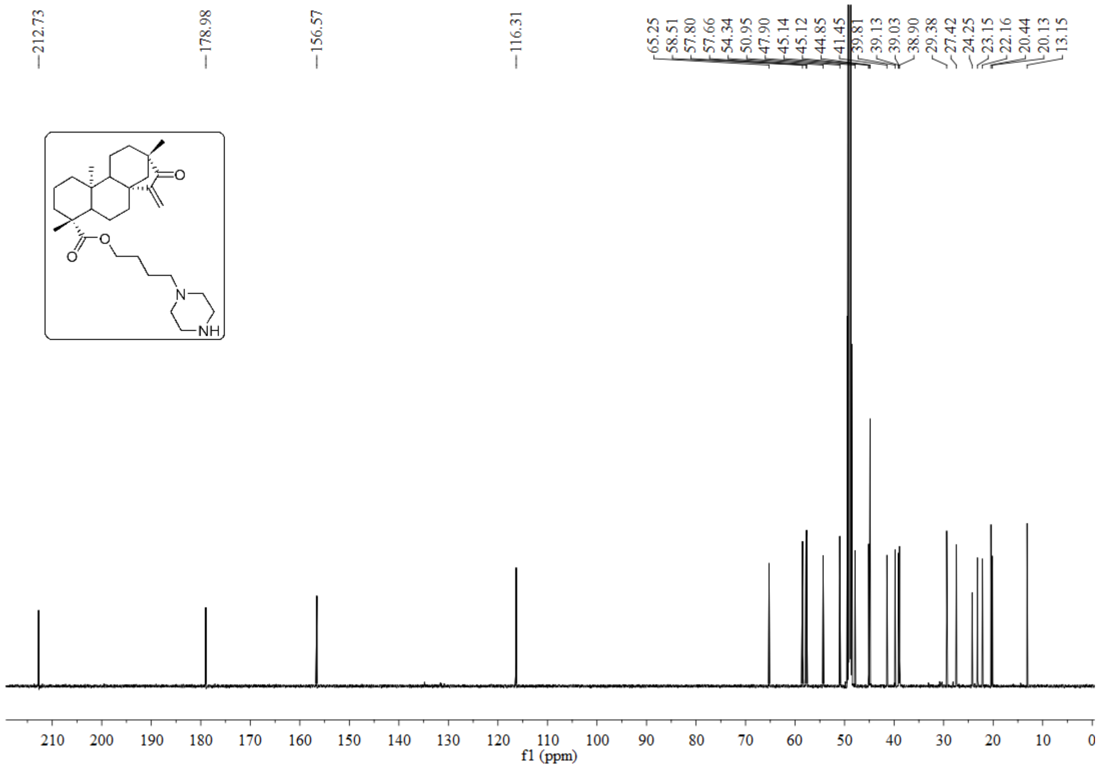

Supplementary Figure 69. ^{13}C NMR spectrum of **31**.

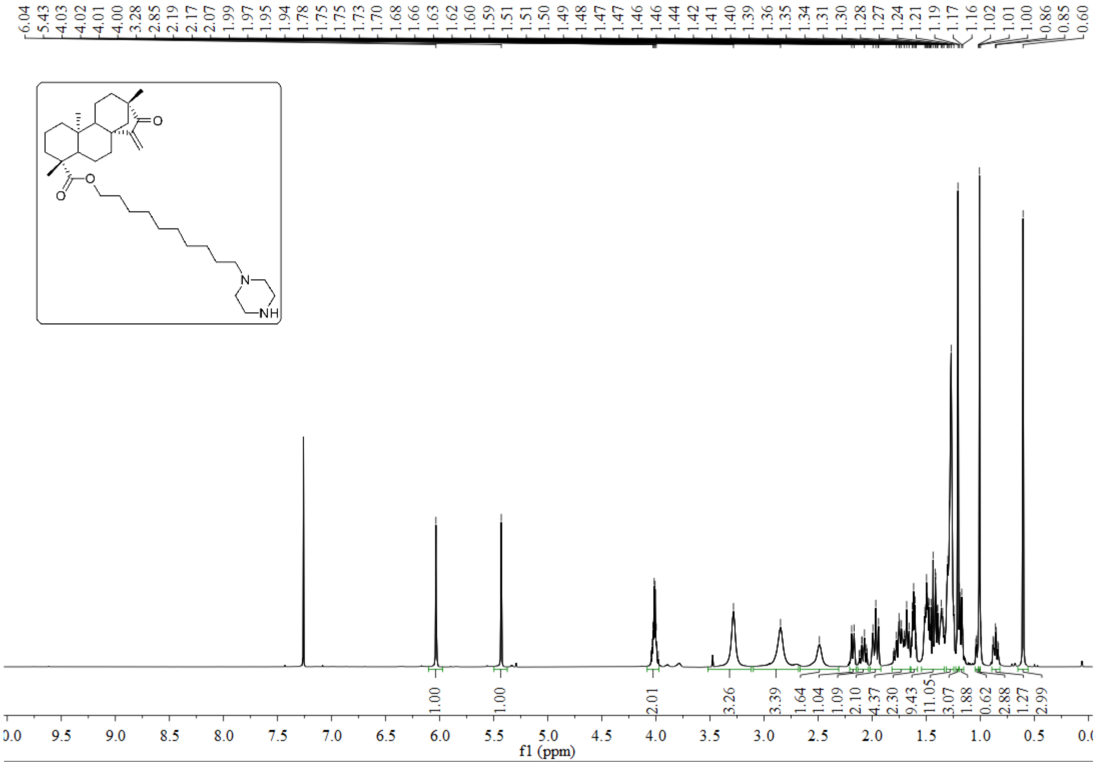

Supplementary Figure 70. ^{1}H NMR spectrum of **32**.

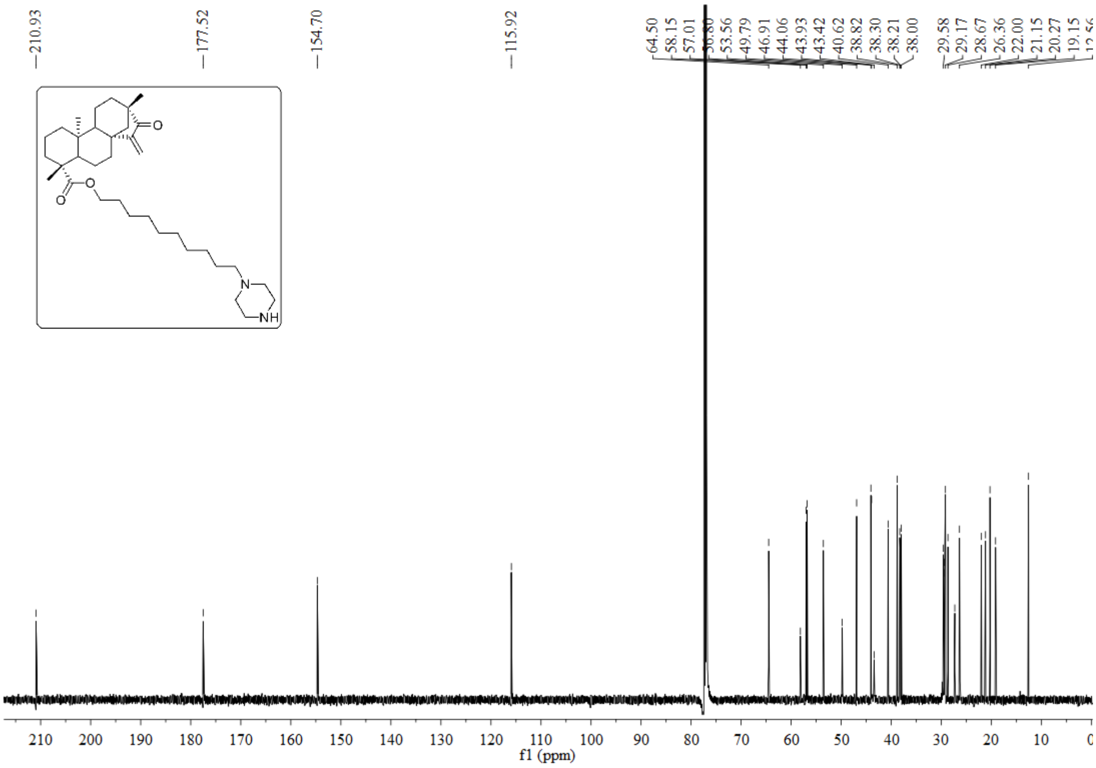

Supplementary Figure 71. ^{13}C NMR spectrum of **32**.

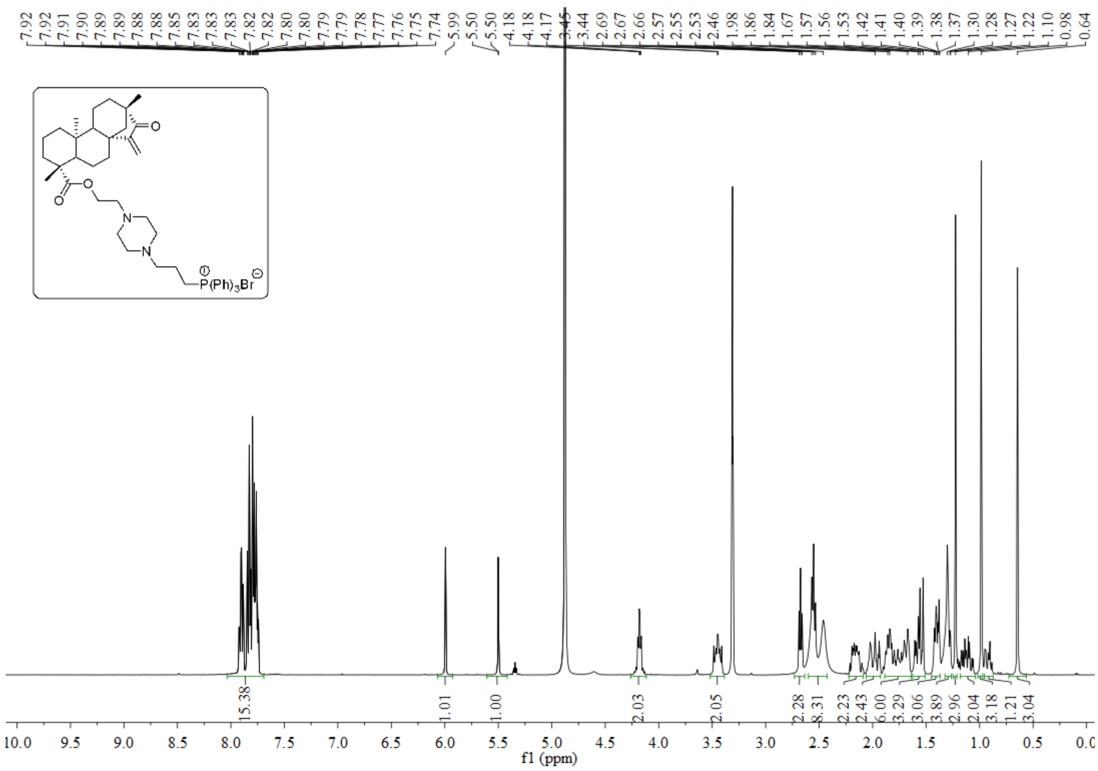

Supplementary Figure 72. ^{1}H NMR spectrum of **33**.

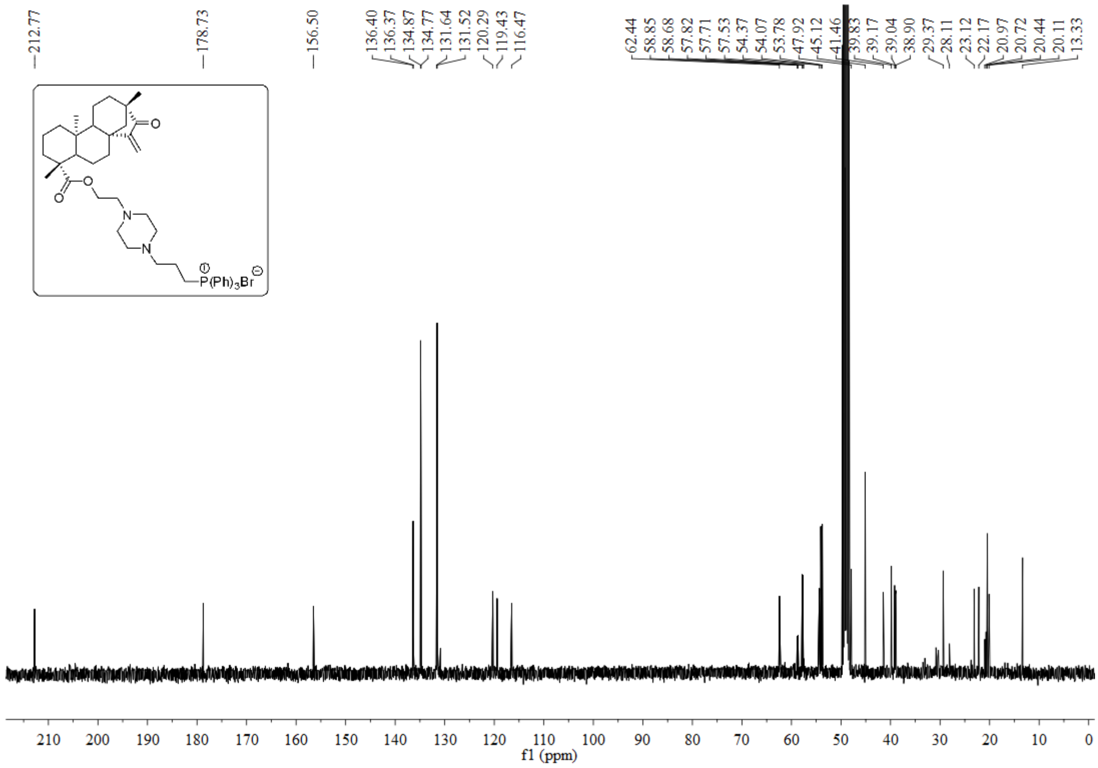

Supplementary Figure 73. ^{13}C NMR spectrum of **33**.

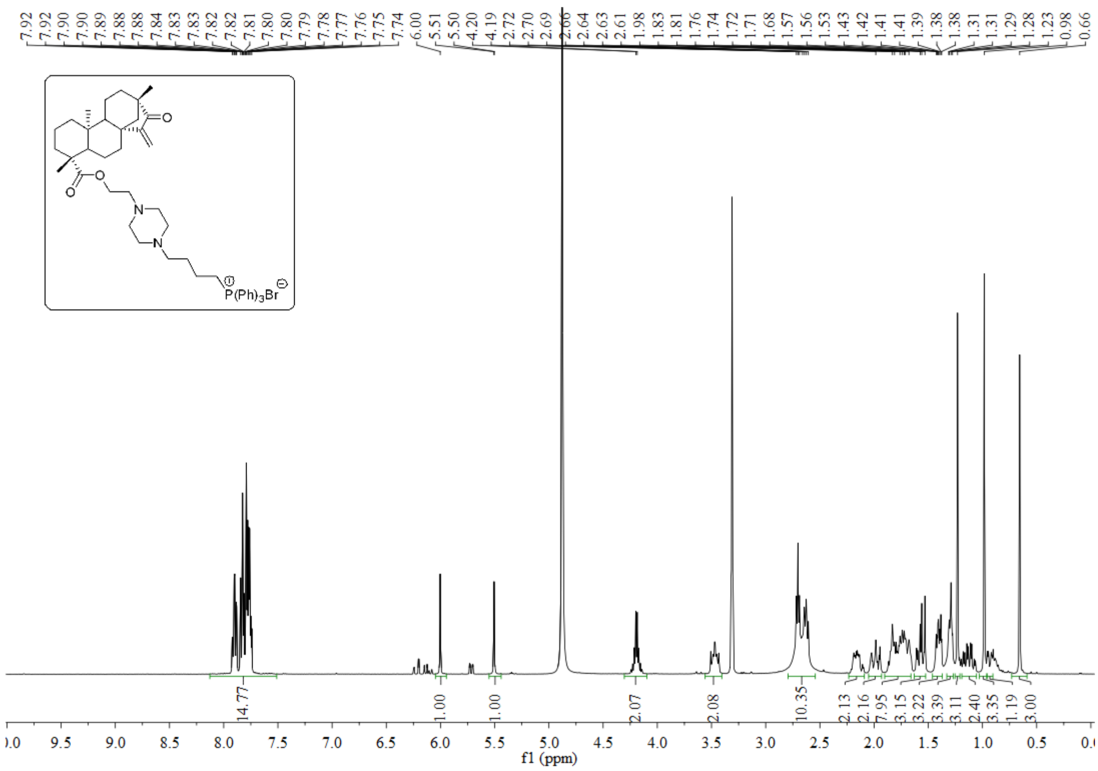

Supplementary Figure 74. ^{1}H NMR spectrum of **34**.

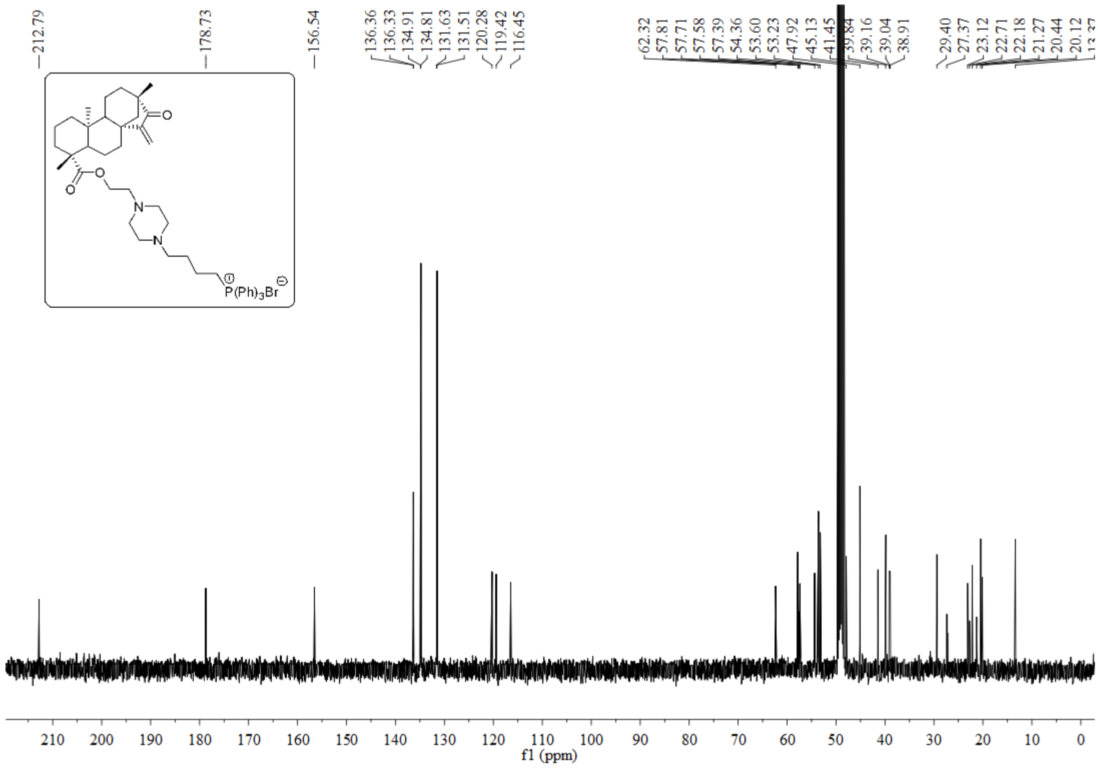

Supplementary Figure 75. ^{13}C NMR spectrum of **34**.

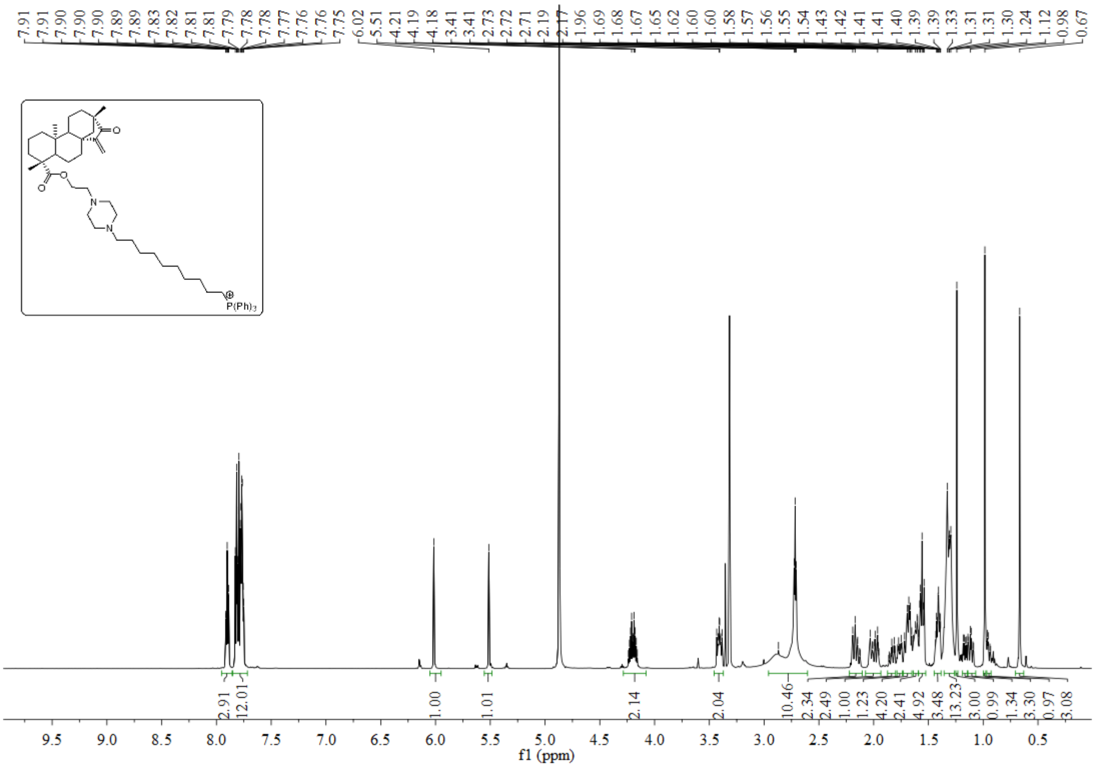

Supplementary Figure 76. ^{1}H NMR spectrum of **35**.

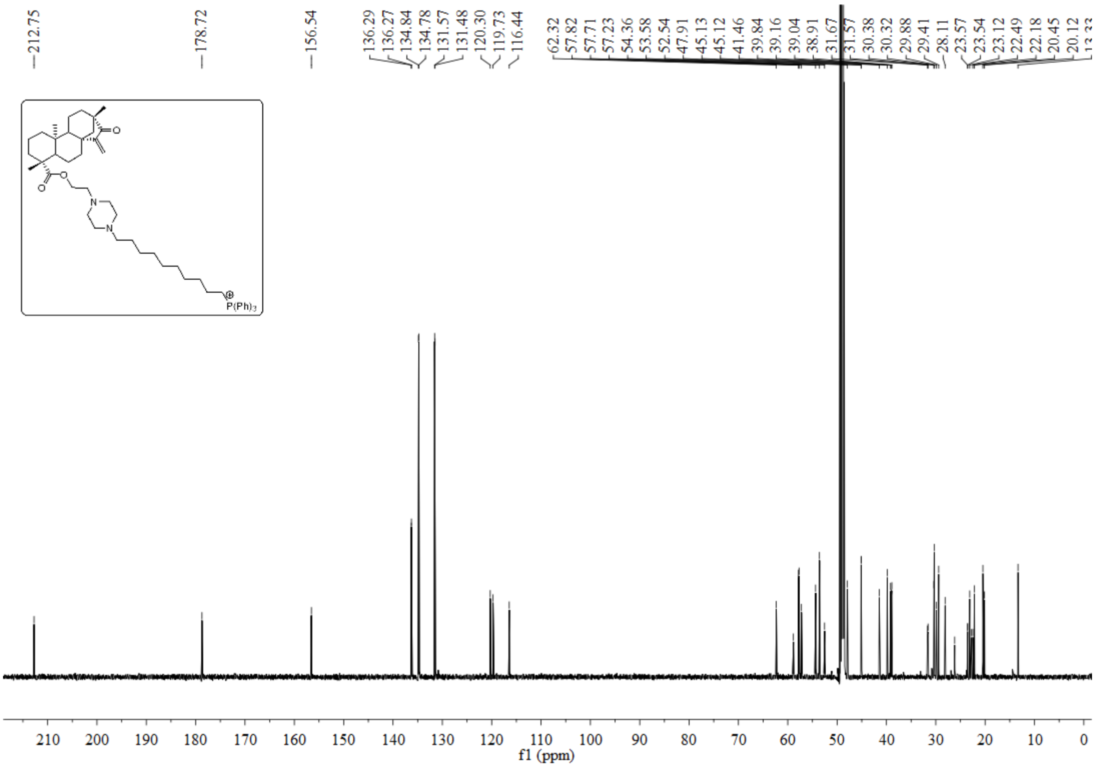

Supplementary Figure 77. ^{13}C NMR spectrum of **35**.

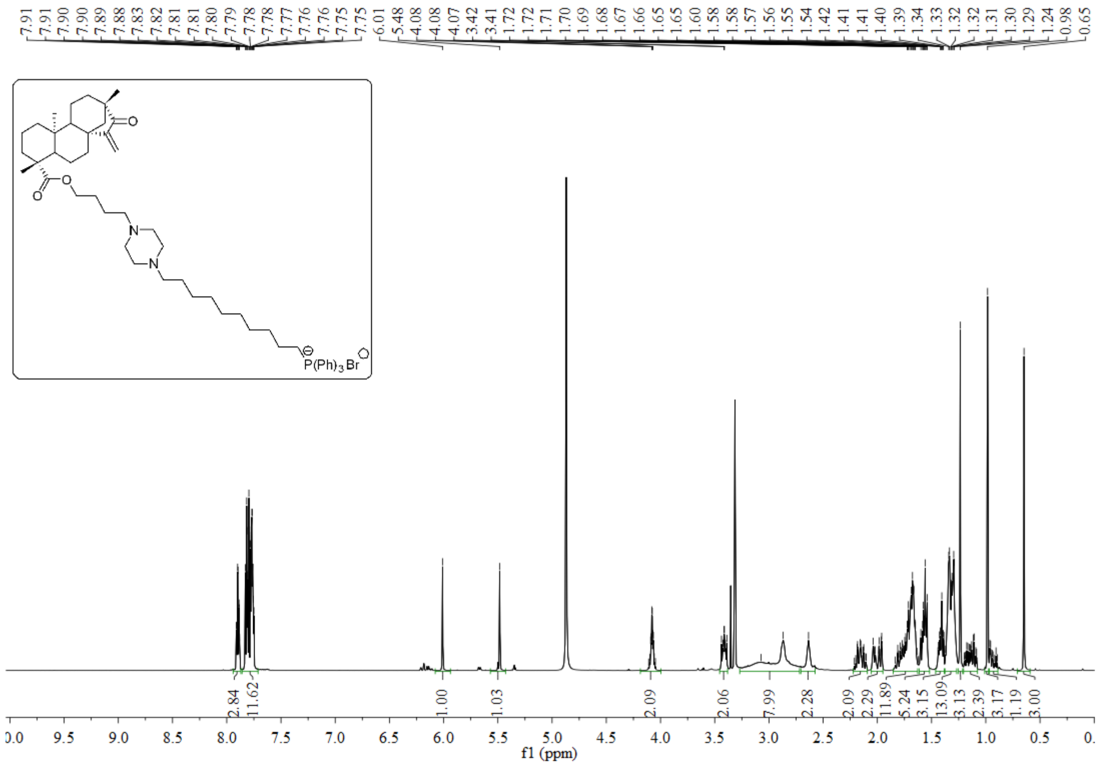

Supplementary Figure 78. ^{1}H NMR spectrum of **36**.

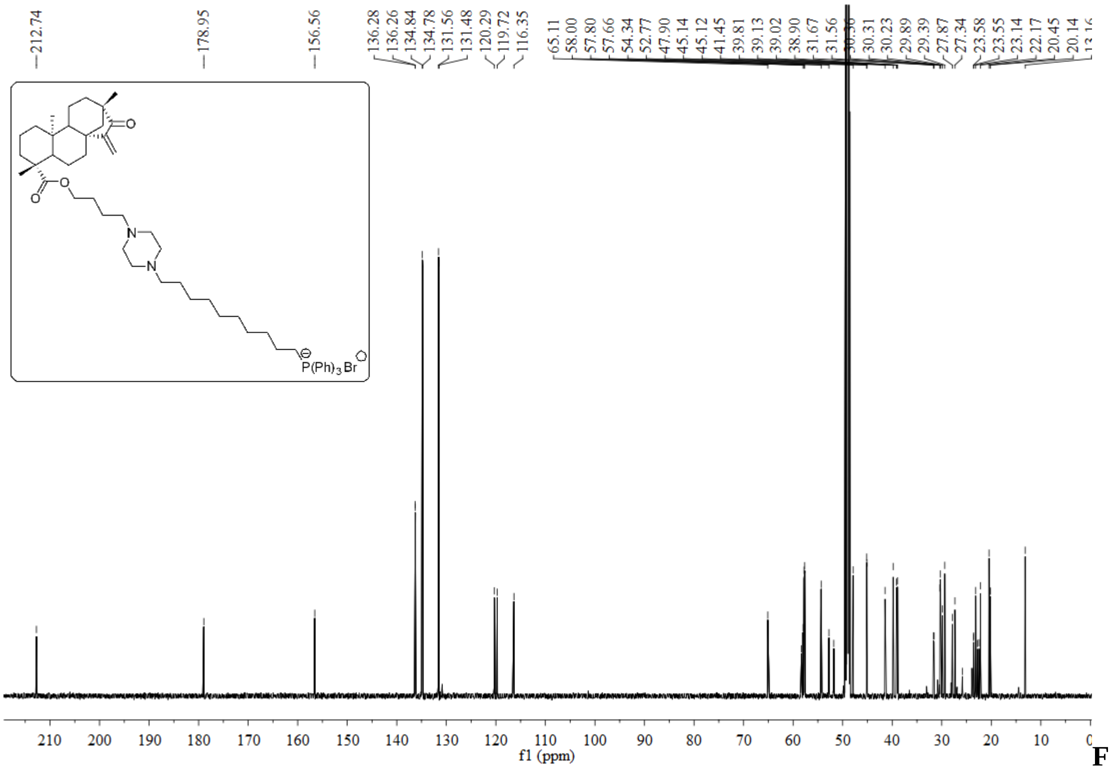

Supplementary Figure 79. ^{13}C NMR spectrum of **36**.

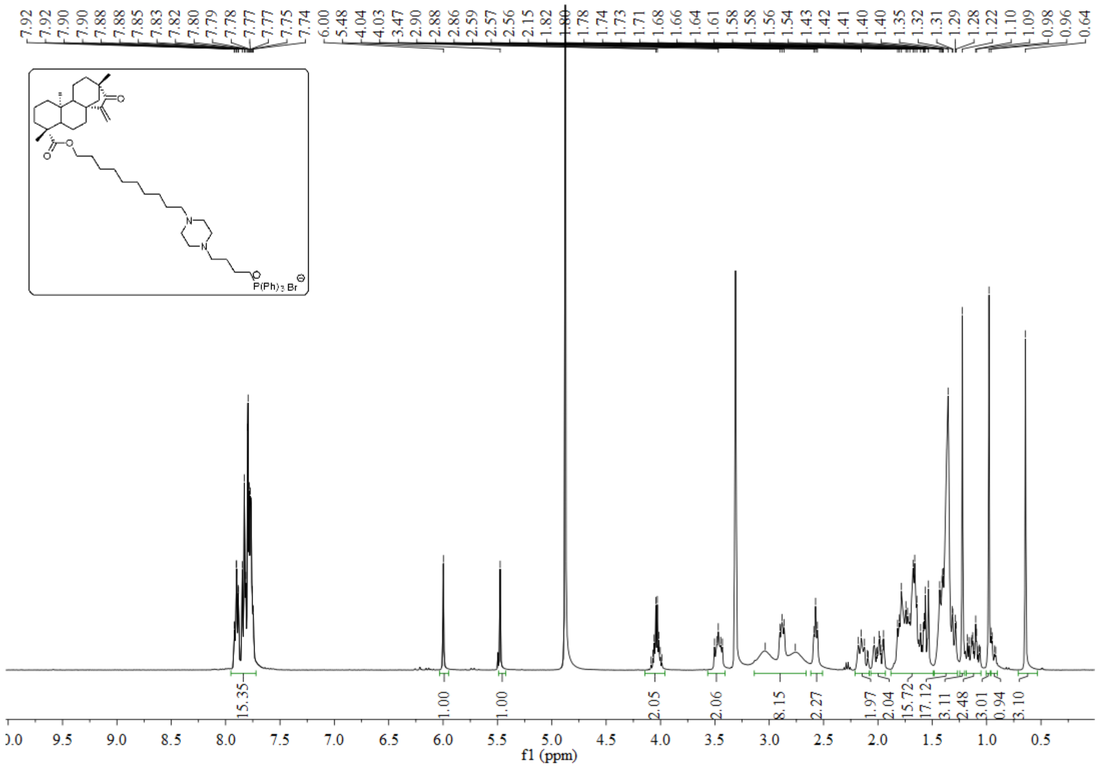

Supplementary Figure 80. ^{1}H NMR spectrum of **37**.

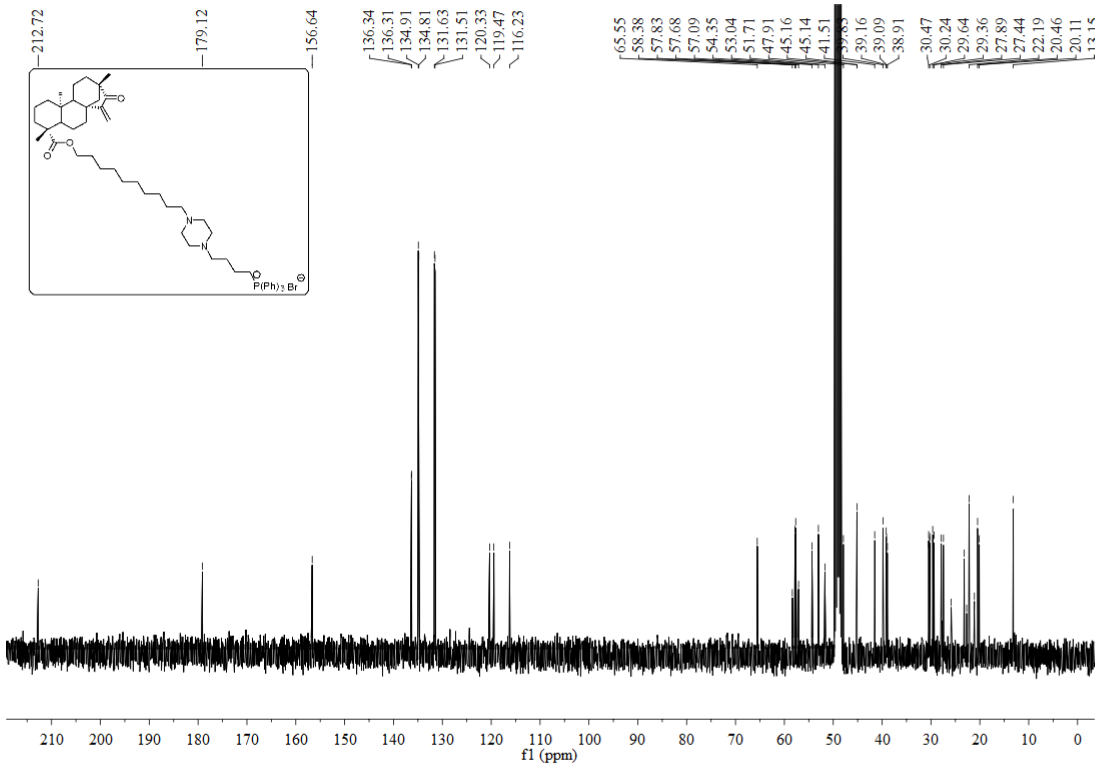

Supplementary Figure 81. ^{13}C NMR spectrum of **37**.

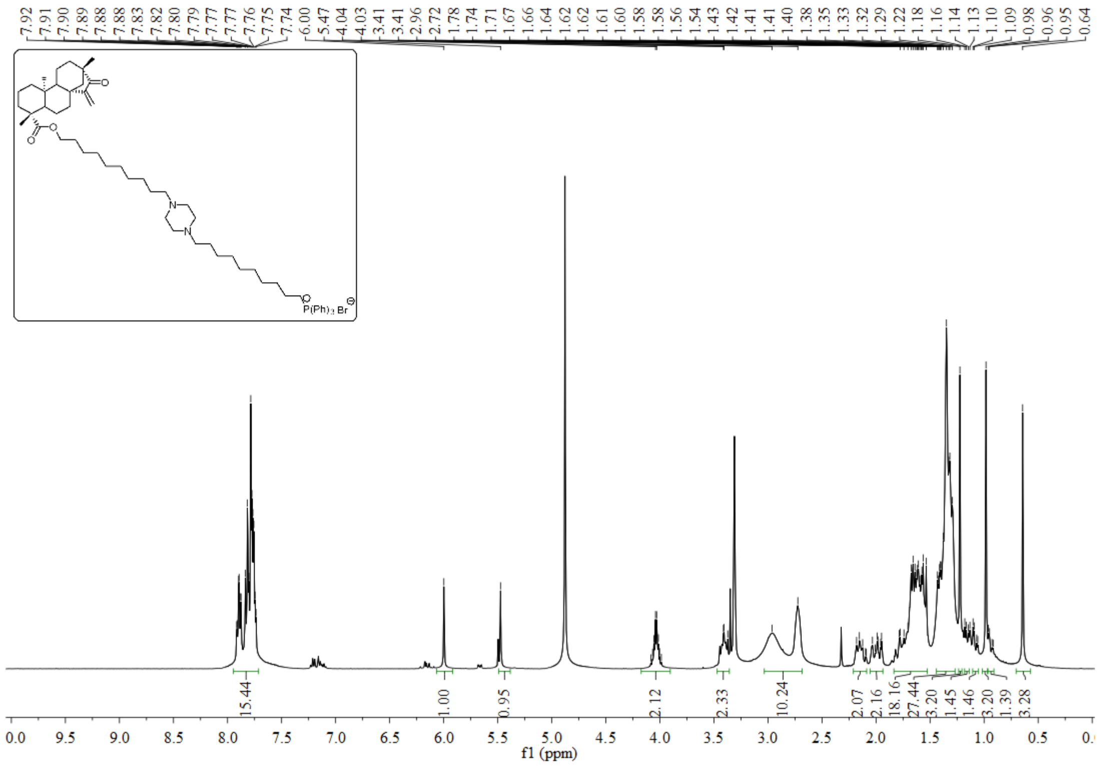

Supplementary Figure 82. ^{1}H NMR spectrum of **38**.

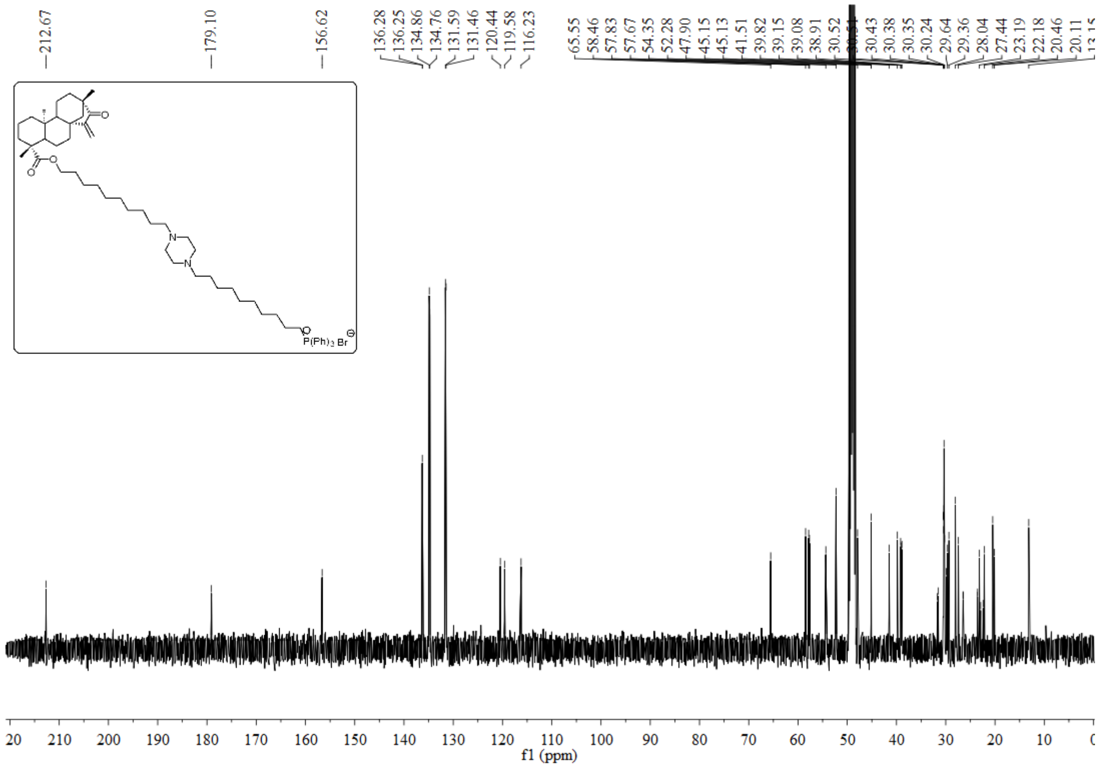

Supplementary Figure 83. ^{13}C NMR spectrum of **38**.

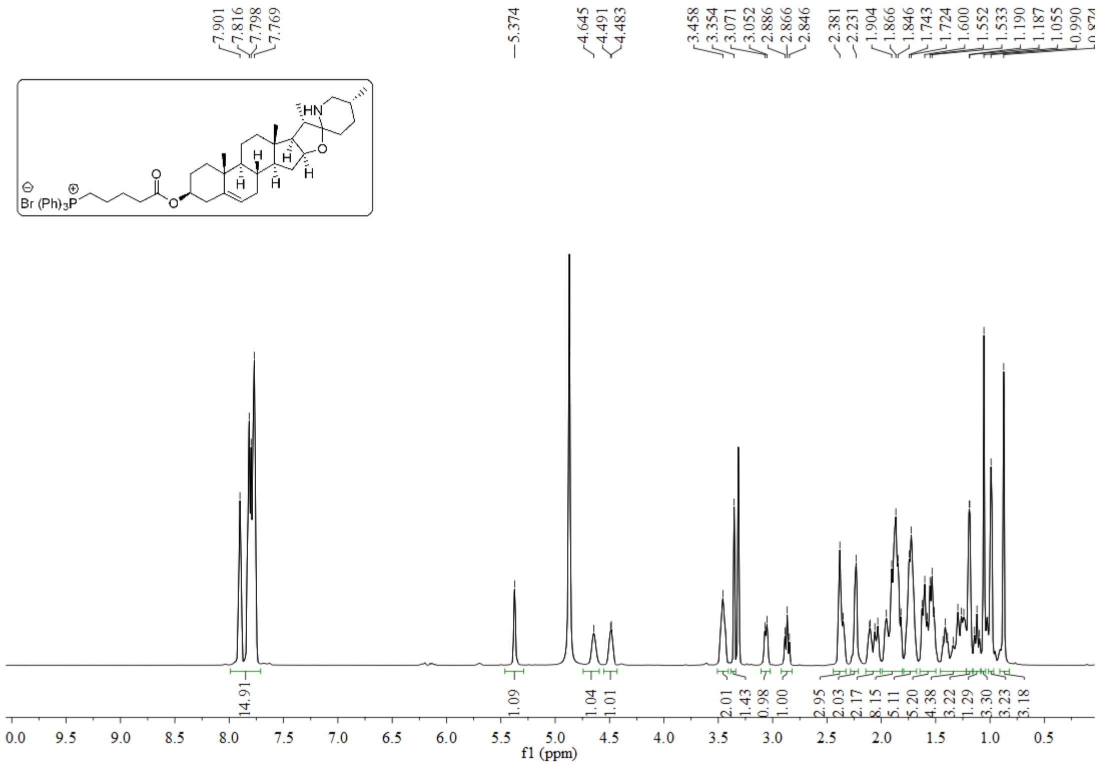

Supplementary Figure 84. ^{1}H NMR spectrum of **39**.

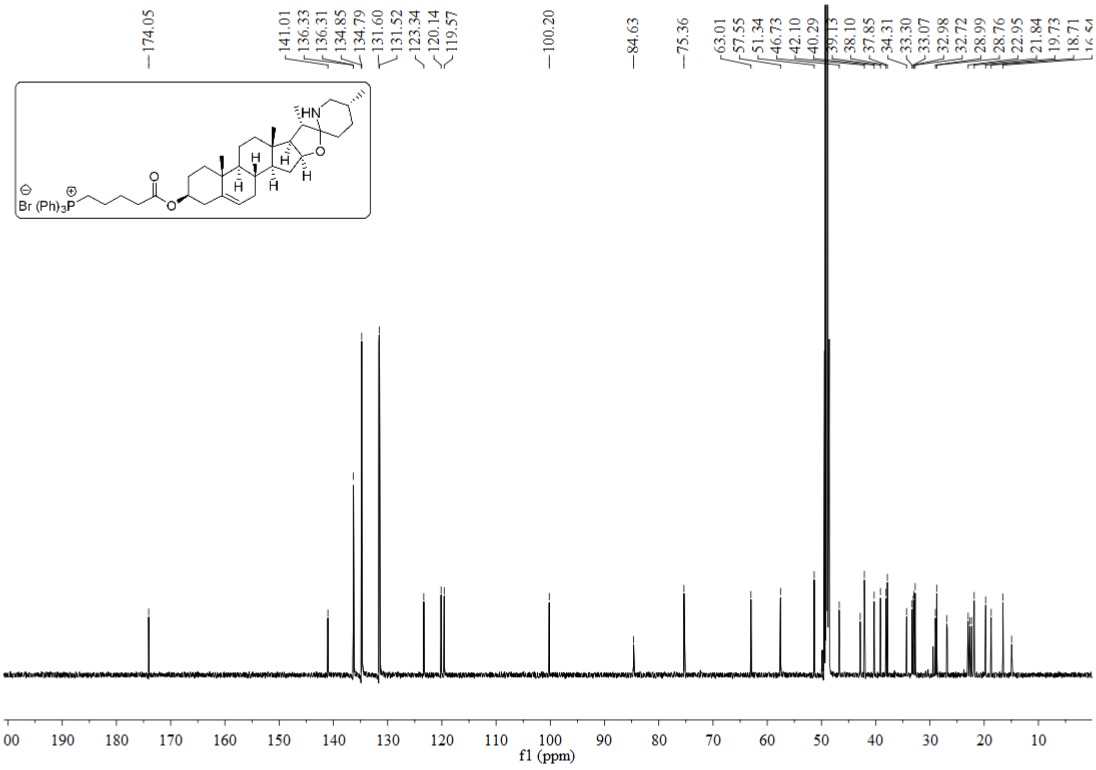

Supplementary Figure 85. ^{13}C NMR spectrum of **39**.

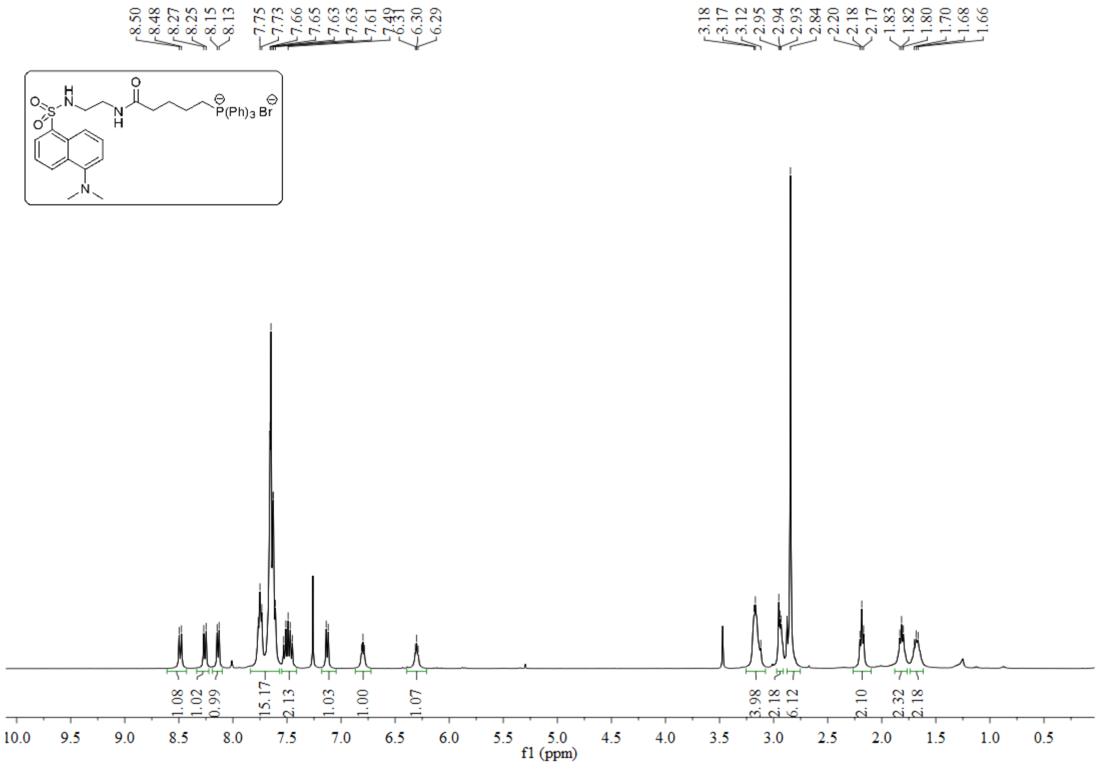

Supplementary Figure 86. ^{1}H NMR spectrum of **41**.

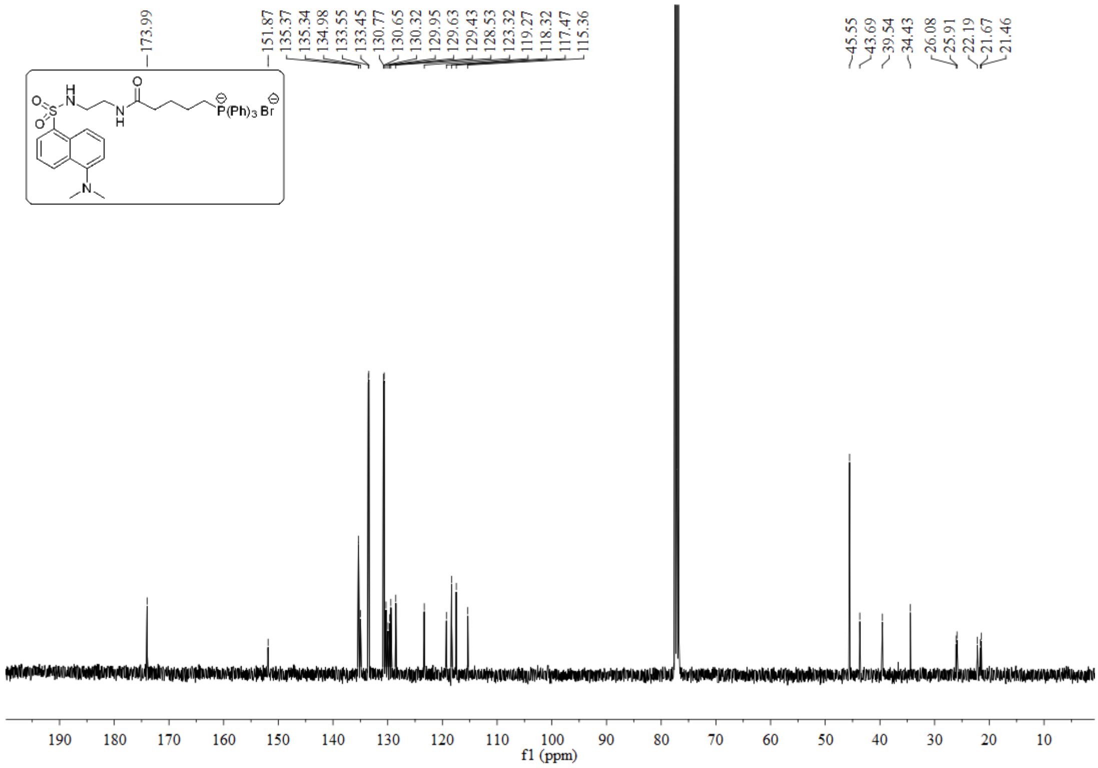

Supplementary Figure 87. ^{13}C NMR spectrum of **41**.

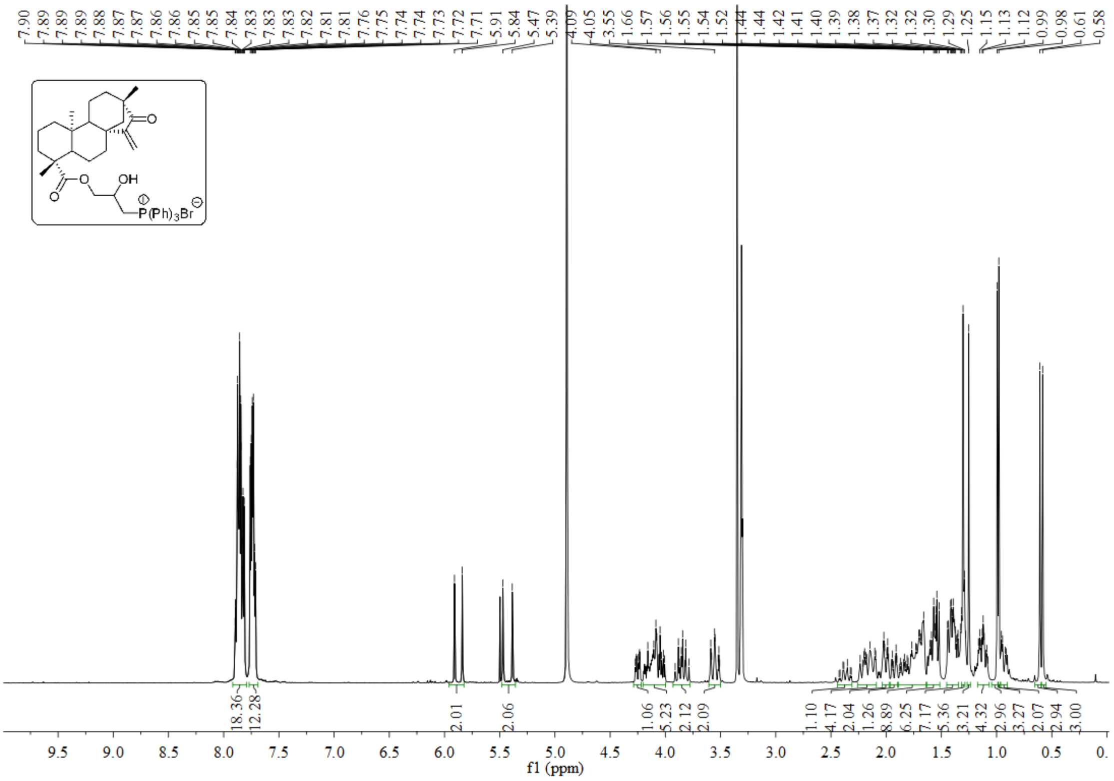

Supplementary Figure 88. ^{1}H NMR spectrum of **44**.

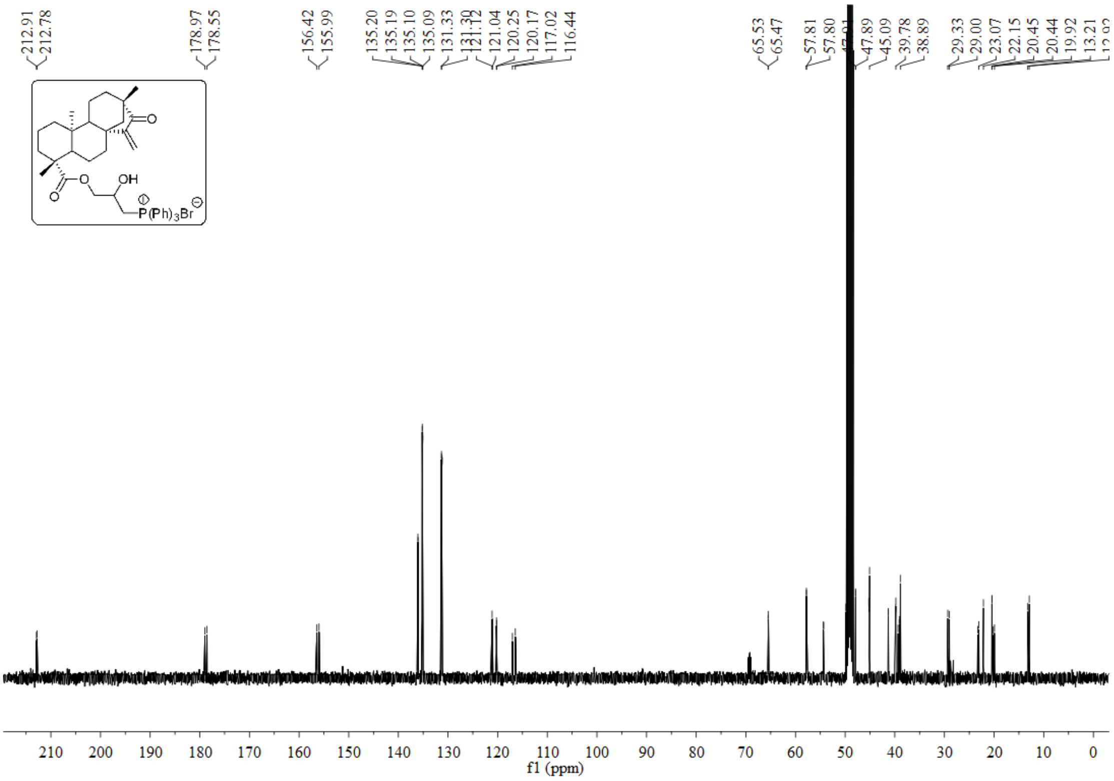

Supplementary Figure 89. ^{13}C NMR spectrum of **44**.

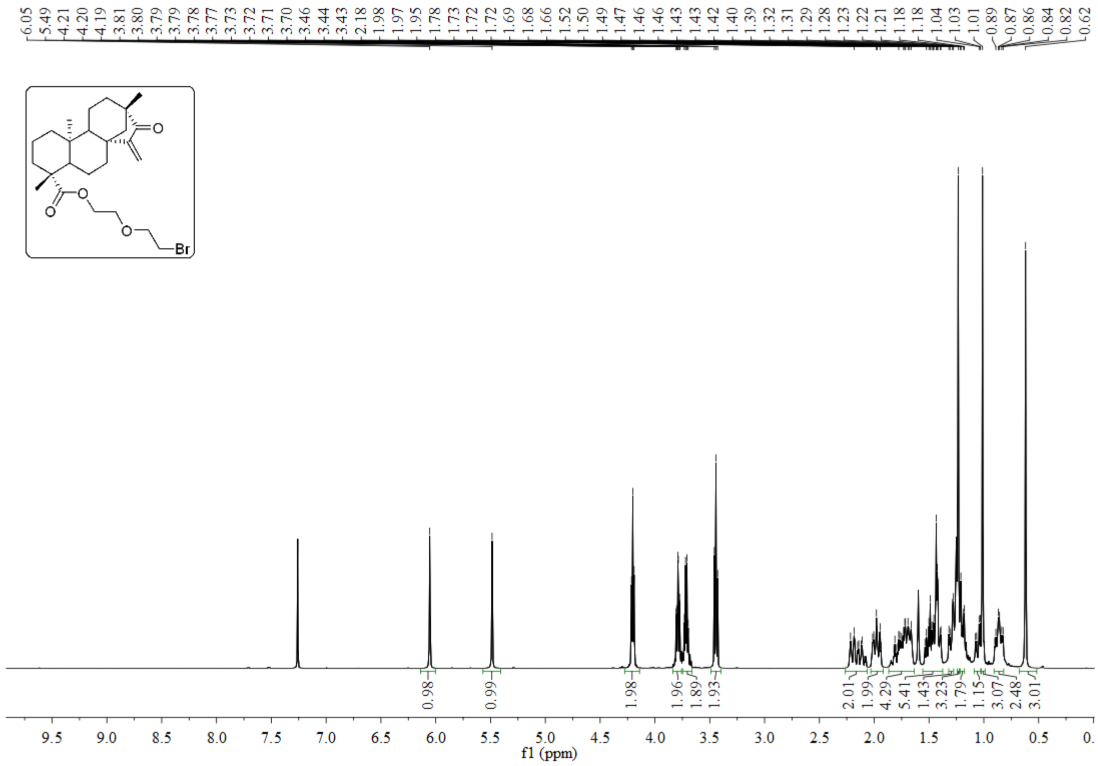

Supplementary Figure 90. ^{1}H NMR spectrum of **45**.

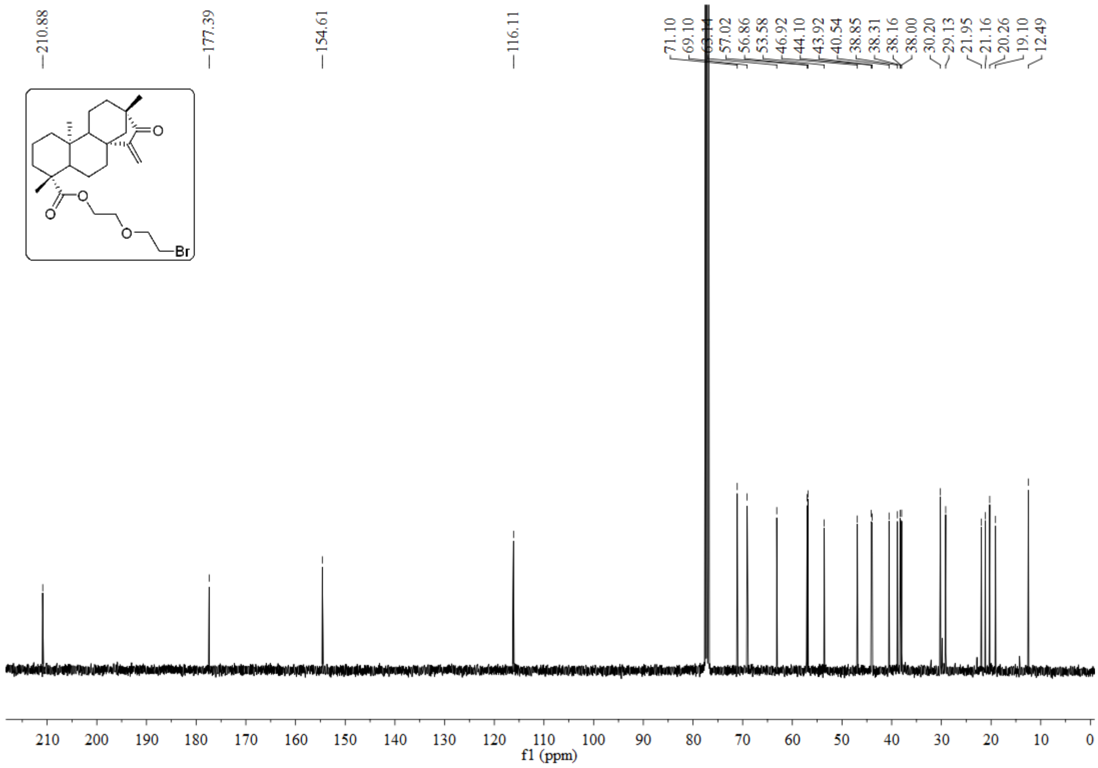

Supplementary Figure 91. ^{13}C NMR spectrum of **45**.

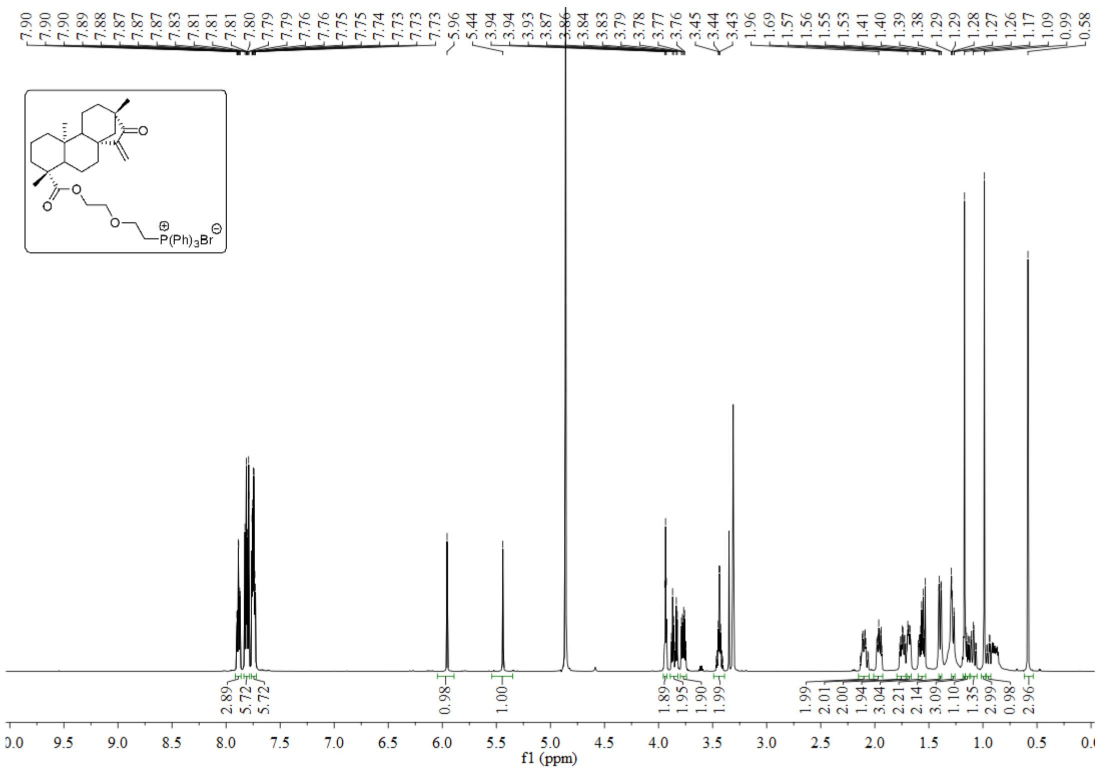

Supplementary Figure 92. ^{1}H NMR spectrum of **46**.

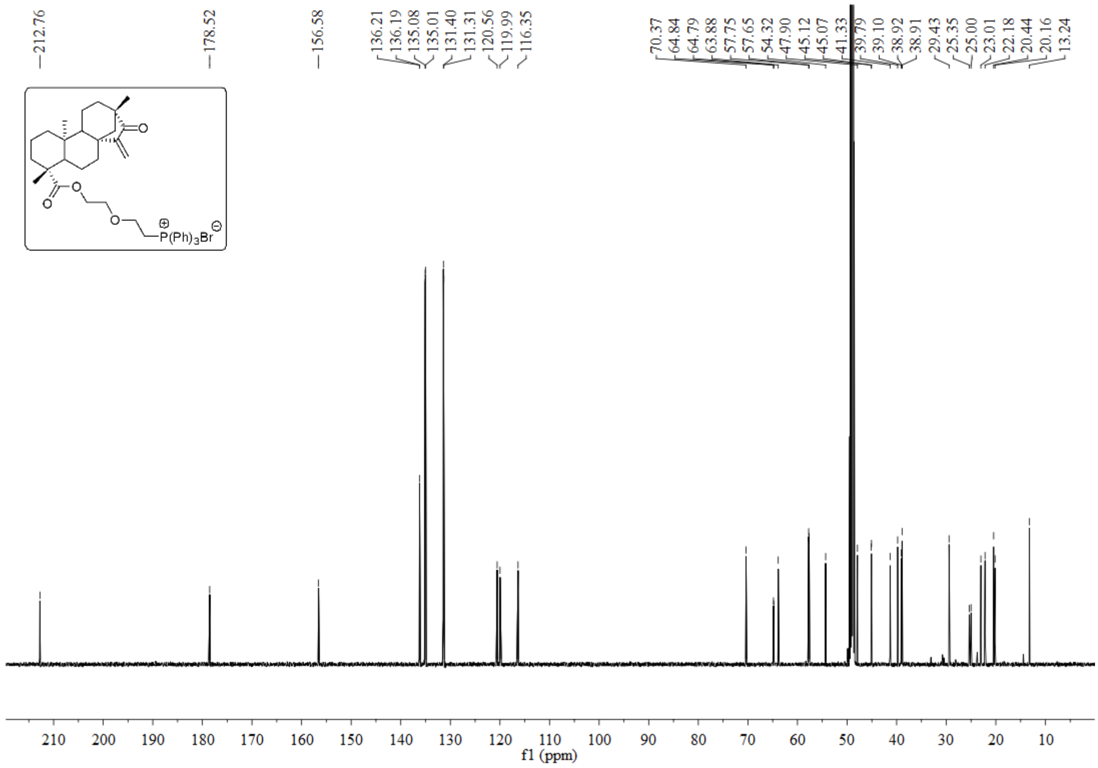

Supplementary Figure 93. ^{13}C NMR spectrum of **46**.

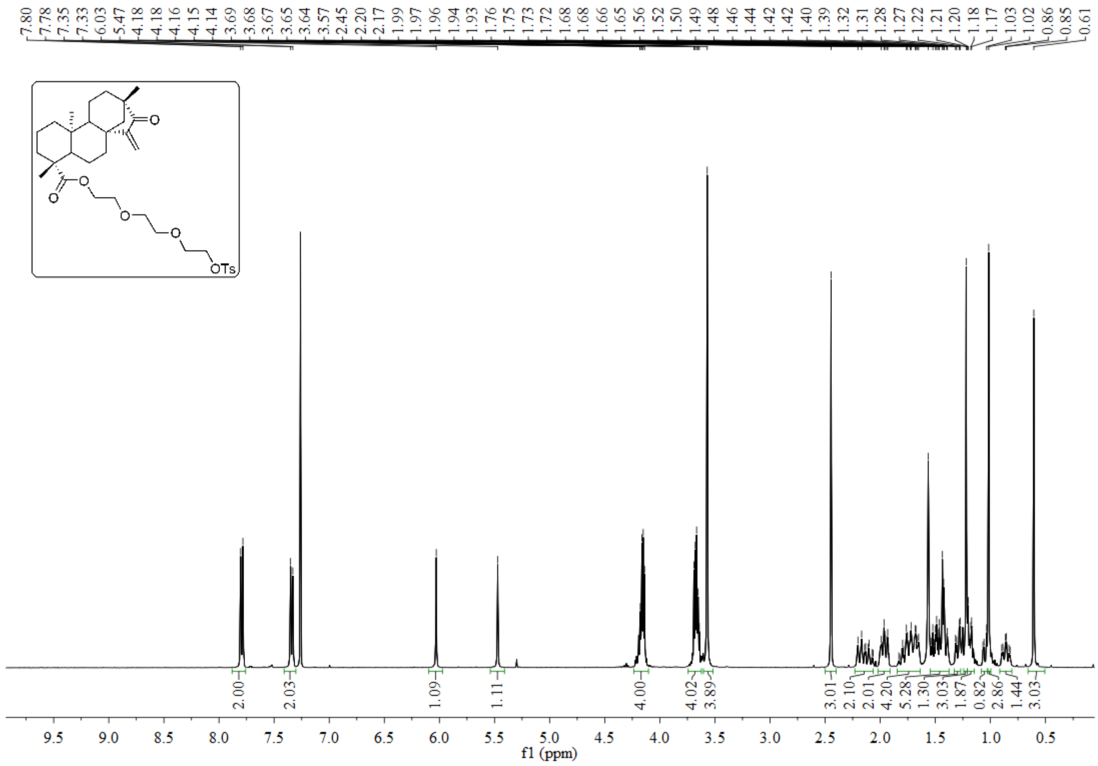

Supplementary Figure 94. ^{1}H NMR spectrum of **47**.

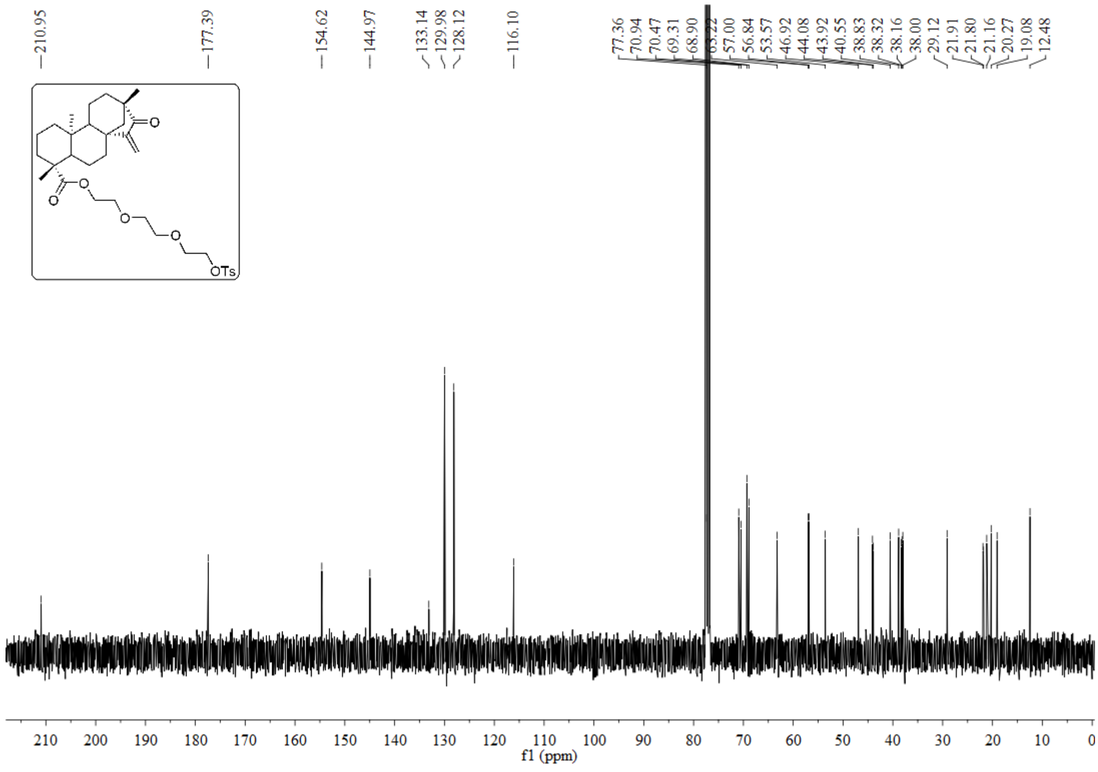

Supplementary Figure 95. ^{13}C NMR spectrum of **47**.

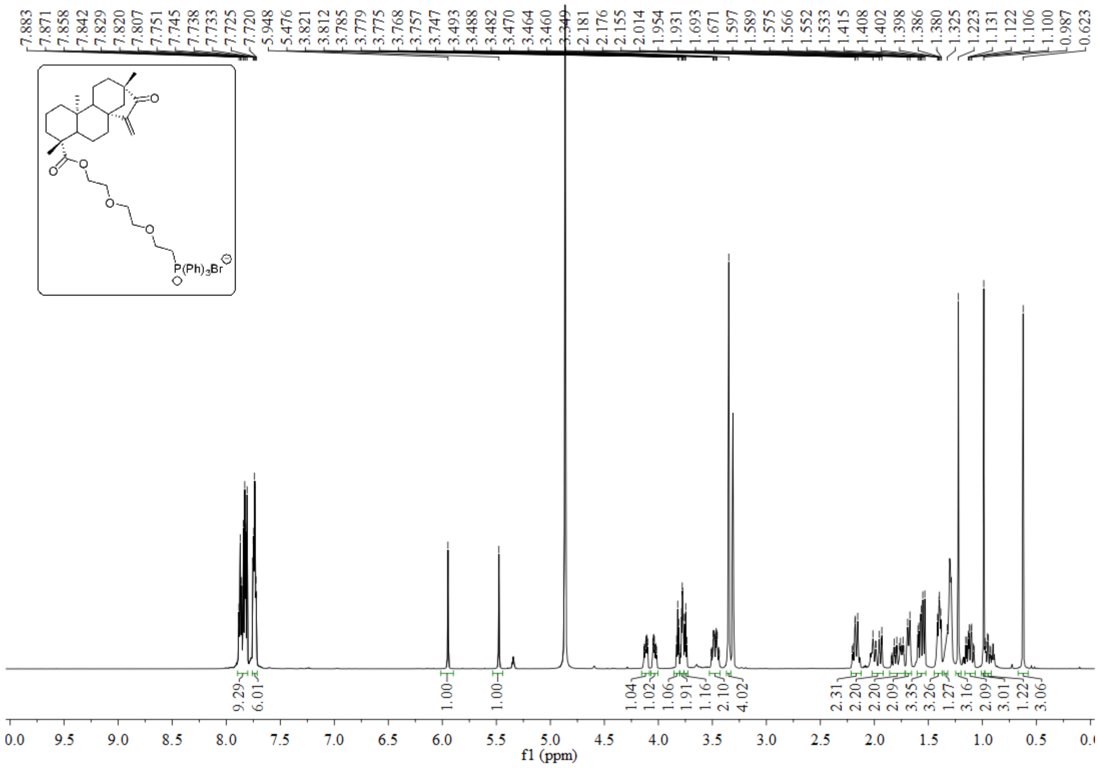

Supplementary Figure 96. ^{1}H NMR spectrum of **49**.

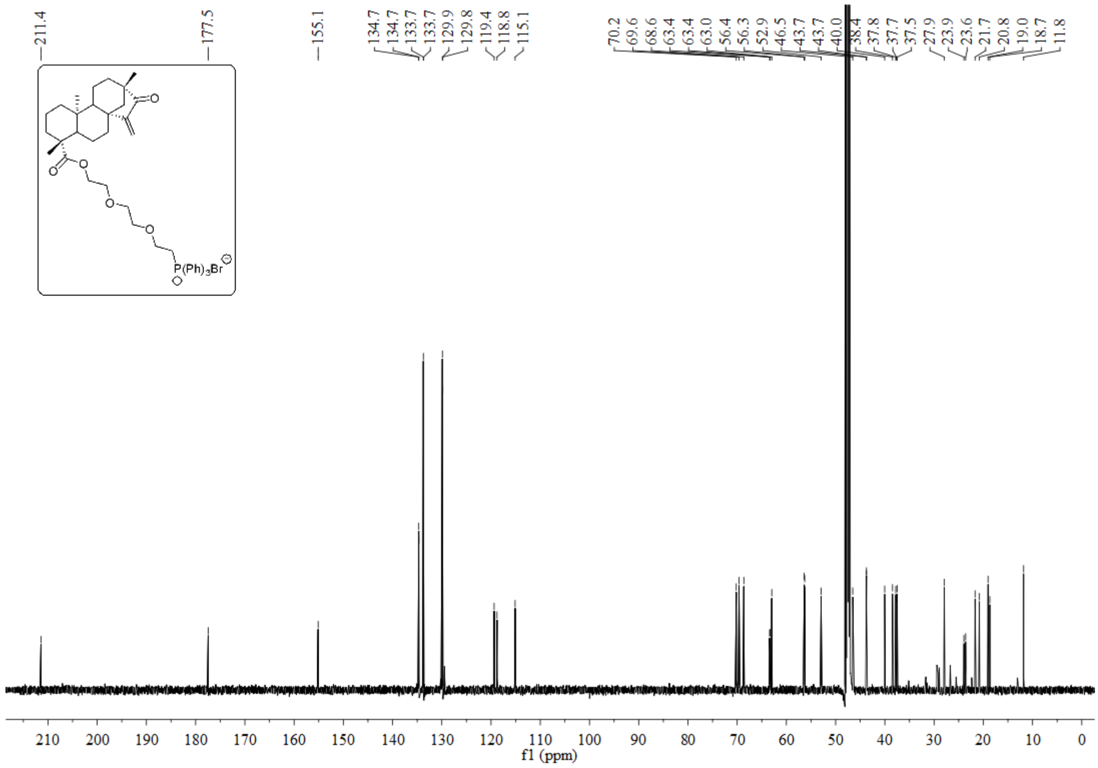

Supplementary Figure 97. ^{13}C NMR spectrum of **49**.

Supplementary Table 1. MFC values of representative compounds. [a]

Compounds	MFC (μg/mL)				
	SC5314	YEM13	YEM15	28A	28I
DBC-TPP	8	8	8	16	8
Borneol-TPP	16	16	16	16	16
IS-1-TPP	8	16	16	16	8
IS-2-TPP	2	4	4	4	4
IS-2-Pi-TPP	1	1	2	2	2
Sola-TPP	8	8	8	8	8
AMB	1	0.5	2	2	2

[a] YEM13 is a constructed *C. albicans* strain with hyperexpression of *MDR1*; YEM15 is a constructed *C. albicans* strain with hyperexpression of both *CDR1* and *CDR2; C. albicans* 28A and 28I are clinical pan-azole-resistant strains.

Supplementary Table 2. MIC values of tested compounds against six azole-resistant *C. albicans* strains. [a]

Tested Compounds	MIC (μg/mL) [a]					
	SCMRR1R34MPG2A	SCUPC2R14MPG2A	Gu5	DSY296	F5	G5
DBC-TPP	4	4	8	4	4	4
Borneol-TPP	8	8	8	8	8	8
IS-1-TPP	4	8	8	4	8	8
IS-2-TPP	2	2	4	2	2	2
IS-2-Pi-TPP	1	1	1	1	1	1
Sola-TPP	2	2	4	4	4	4
FLC	>128	8	>128	>128	32	>128
AMB	0.5	0.5	1	0.5	0.5	0.5

[a] SCMRR1R34MPG2A (*MRR1*^{P683S}/*MRR1*^{P683S}); SCUPC2R14MPG2A (*UPC2*^{G648D}/*UPC2*^{G648D}); Gu5 (an FLC-resistant clinical isolate from patient Gu with G980E/G980E in *TAC1*); DSY296 (an FLC-resistant clinical isolate with N977D/N977D in *TAC1*); F5 (an FLC-resistant clinical isolate from patient F with P683S/P683S in *MRR1*, G648S/G648S in *UPC2*); G5 (an FLC-resistant clinical isolate from patient G with G997V/G997V in *MRR1*)

Supplementary Table 3. MFC values of tested compounds against six azole-resistant *C. albicans* strains.

Tested Compounds	MFC (μg/mL)					
	SCMRR1R 34MPG2A	SCUPC2R1 4MPG2A	Gu5	DSY296	F5	G5
DBC-TPP	8	8	16	8	8	8
Borneol-TPP	16	16	16	16	16	16
IS-1-TPP	8	8	16	8	8	8
IS-2-TPP	4	4	4	4	4	4
IS-2-Pi-TPP	2	2	2	2	2	2
Sola-TPP	4	4	8	8	8	8
AMB	1	1	2	2	1	2

Supplementary Table 4. Determination of intracellular content of tested agents in four *C. albicans* strains.[a]

Compound	Content (μg per 10^8 cells)			
	SC5314	YEM13	YEM15	DSY654[b]
IS-1	B.T.D.	B.T.D.	B.T.D.	B.T.D.
IS-1-TPP	10.35 ± 1.71	—	—	—
IS-2	B.T.D.	B.T.D.	B.T.D.	0.47±0.06
IS-2-TPP	13.50 ± 2.92	3.65 ± 0.25	10.7 ± 1.0	21.21 ± 1.24
IS-2-Pi-TPP	32.32 ± 5.54	20.06 ± 1.48	20.02 ± 2.18	60.22 ± 8.80
**46**	26.46 ± 2.33	—	—	—
DBC	B.T.D.	B.T.D.	B.T.D.	2.64±0.39
DBC-TPP	20.55 ± 4.72	11.42 ± 1.84	26.34 ± 3.46	53.68 ± 8.06
Solasodine	B.T.D.	—	—	—
Sola-TPP	5.80 ± 0.35	—	—	—

[a] The values are expressed as mean ± s.e.m. B.T.D. represent below the threshold of detection. '—' = non-detected.

[b] DSY654, *cdr1* and *cdr2* double mutant.

Supplementary Table 5. Antifungal activities of selected compounds against efflux pump-deficient *C. albicans* strains. [a]

Compounds	MIC (μg/mL)					MFC (μg/mL)				
	DSY 448	DSY653	DSY 654	DSY 465	DSY 1050	DSY 448	DSY 653	DSY 654	DSY465	DSY 1050
IS-1	>128	>128	>128	>128	>128	>128	>128	>128	>128	>128
IS-1-TPP	8	8	8	8	8	8	8	8	8	8
IS-2	>128	>128	64	>128	64	>128	>128	>128	>128	128
IS-2-TPP	4	2	2	4	2	4	4	4	4	4
IS-2-Pi-TPP	2	2	1	2	2	2	2	2	4	2
Borneol-TPP	8	8	4	8	4	8	8	8	8	4
DBC-TPP	4	4	4	4	4	4	4	4	4	4
Sola-TPP	8	4	4	4	4	8	8	4	4	4
**12**	>128	>128	>128	>128	>128	>128	>128	>128	>128	>128
**17**	8	8	8	8	8	8	8	16	8	8
**21**	4	4	4	4	2	4	4	4	4	4
**23**	2	1	1	1	2	2	1	2	1	2
**45**	>128	>128	>128	>128	>128	>128	>128	>128	>128	>128
**47**	>128	>128	>128	>128	>128	>128	>128	>128	>128	>128
**49**	2	4	1	2	2	2	4	1	2	2
FLC[b]	0.5	0.5	0.5	1	0.25					
AMB[b]	2	2	1	2	0.5	2	2	2	2	0.5

[a] DSY448, *cdr1* mutant; DSY653, *cdr2* mutant; DSY654, *cdr1* and *cdr2* double mutant; DSY465, *mdr1* mutant; DSY1050, *cdr1*, *cdr2* and *mdr1* triple mutant.

Supplementary Table 6. MIC values of tested IS-2 derivatives against five *C. albicans* strains.

Compounds	MIC (μg/mL)				
	SC5314	YEM13	YEM15	28A[b]	28I[b]
**12**	>128	>128	>128	>128	>128
**16**	>128	>128	>128	>128	>128
**17**	8	16	8	16	8
**20**	>128	>128	>128	>128	>128
**21**	4	8	8	8	8
**23**	2	1	2	1	1
**25**	>128	>128	>128	>128	>128
**28**	2	2	2	2	2
**29**	>128	>128	>128	>128	>128
**30**	>128	>128	>128	>128	>128
**33**	2	2	1	2	2
**34**	2	2	2	2	2
**35**	0.5	0.5	0.5	1	1
**36**	4	2	2	2	2
**37**	4	2	2	2	2
**38**	8	4	4	4	8
**44**	2	4	4	2	4
**45**	>128	>128	>128	>128	>128
**46**	4	4	4	4	4
**47**	>128	>128	>128	>128	>128
**49**	2	2	2	2	2

Supplementary Table 7. Antifungal activities of selected compounds against other *Candida* species.[a]

Compounds	MIC (μg/mL)				MFC (μg/mL)			
	CK1	CG2	CP1	CT2	CK1	CG2	CP001	CT2
IS-1	>128	>128	>128	>128	>128	>128	>128	>128
IS-1-TPP	4	64	8	8	4	128	32	16
IS-2	>128	>128	>128	>128	>128	>128	>128	>128
IS-2-TPP	2	16	4	4	2	32	8	8
IS-2-Pi-TPP	2	4	4	2	2	4	4	2
Borneol-TPP	8	16	16	8	8	32	16	8
DBC-TPP	4	8	8	8	4	16	16	16
Sola-TPP	4	4	16	4	8	8	16	4
**12**	>128	>128	>128	>128	>128	>128	>128	>128
**17**	4	>64	32	16	4	>64	32	16
**21**	2	>8	8	8	2	>8	8	8
**23**	4	8	8	4	4	8	8	4
**45**	>128	>128	>128	>128	>128	>128	>128	>128
**47**	>128	>128	>128	>128	>128	>128	>128	>128
**49**	2	>8	4	8	2	>8	8	8
FLC	2	8	1	2				
AMB	0.5	0.5	0.5	0.5	1	1	1	1

[a]CK1 represents *Candida krusei* strain 1, CG2 represents *Candida glabrata* strain 2, CP1 represents *Candida parapsilosis* strain 1, and CT2 represents *Candida tropicalis* strain 2.

Supplementary Table 8. The alteration of MIC values for indicated TPP^+-conjugates during 30 passages of drug induction.

Compounds	MIC (μg/mL)								
	passage	0	6	8	13	17	20	28	30
IS-2-Pi-TPP		1	1	1	1	1	1	1	1
Sola-TPP		4	4	4	4	4	4	4	4
Borneol-TPP		8	8	16	16	16	16	16	16
FLC		2	4	4	8	16	32	64	64

Supplementary Methods

Strains and growth conditions. The *Candida* isolates used in this study are shown in Table **1** and the Supplementary tables. SCMRR1R34MPG2A ($MRR1^{P683S}$/$MRR1^{P683S}$) [1], SCUPC2R14MPG2A ($UPC2^{G648D}$/$UPC2^{G648D}$) [2], Gu5 (an FLC-resistant clinical isolate from patient Gu with G980E/G980E in *TAC1*) [3], DSY296 (an FLC-resistant clinical isolate with N977D/N977D in *TAC1*) [4], F5 (an FLC-resistant clinical isolate from patient F with P683S/P683S in *MRR1*, G648S/G648S in *UPC2*) [5], G5 (an FLC-resistant clinical isolate from patient G with G997V/G997V in *MRR1*) [5] were kind gifts from Professor Joachim Morschhäuser in the University of Würzburg, Germany. The efflux pump-related strains (DSY448, *Δcdr1::hisG-URA3-hisG/Δcdr1::hisG*; DSY653, *Δcdr2::hisG-URA3-hisG/Δcdr2::hisG*; DSY465, *Δmdr1::hisG-URA3-hisG/Δmdr1::hisG*; DSY654, *Δcdr1::hisG/Δcdr1::hisG Δcdr2::hisG-URA3-hisG/Δcdr2::hisG*; DSY1050, *Δcdr1::hisG/Δcdr1::hisG Δcdr2::hisG /Δcdr2::hisG Δmdr1::hisG-URA3-hisG/Δmdr1::hisG*; YEM13, hyperexpressing *MDR1*; YEM15, hyperexpressing *CDR1* and *CDR2*) [6-9] were kindly donated by Kim Lewis from Northeastern University, USA. Clinical *C. albicans* strains 28A and 28I were donated by Professor Qingguo Qi from Shandong University, China. Isolates of *C. krusei*, *C. glabrata*, and *C. tropicalis* were kindly provided by the Central Hospital of Jinan City. All the *Candida* strains used in this study were stored in physiological saline supplemented with 20 % glycerol at -80 °C and propagated on yeast-peptone-dextrose (YPD) agar plates (2 % tryptone, 1 % yeast extract, 2 % glucose and 2 % agar) before each experiment. After incubation for 24 h at 30 °C, the cells were inoculated in YPD broth (2 % tryptone, 1 % yeast extract, and 2 % glucose) overnight at 30 °C and 200 rpm.

Construction of fluorescent protein-tagged *C. albicans* strains. *C. albicans* strain *TDH3-RFP*-CAI4 was created by homologous recombination of red fluorescent protein (RFP) sequences into the 3′ end of their open reading frame using a previously described method [10]. The DNA used for the transformation was created by PCR using primers containing an ~70-base pair sequence homologous to the 3′ end of the *TDH3* open reading frame to amplify a cassette containing RFP and a URA3 selectable marker using pRFP-URA3 as the template. The primer sequences used were FP-URA3-F: 5′-GTACGATAACGAATACGGTTACTCCACCAGAGTTGTTGACTTGTTGGAACACGTTG CTAAAGCTTCTGCTGGTGGTGGTTCTAAAGGTGAAGAATTATT-3′ and FP-URA3-R: 5′-

GTAAGATATTTATTTCAAAAACTCATTATATAACTTCAATTAACTAAAAAAATCAAAT CAAAGTCTTCTAGAAGGACCACCTTTGATTG-3′. The PCR product was transformed into auxotrophic mutant strain CAI4. The transformed strain was then spotted on synthetic medium plus dextrose minus uracil (SD−Ura) solid medium and grown at 37 °C for 3 days. The colonies resulting from the transformation were then screened for RFP-positive cells by fluorescence microscopy and further confirmed by PCR amplification.

C. albicans strains *cdr1Δ/Δ*; *cdr2Δ/Δ* (DSY654) [6] were selected on YPD medium plus 5-fluoroorotic acid (5-FOA) to excise *URA3*. The resulting colonies were checked by PCR to obtain DSY655. Then, the green fluorescent protein (GFP) sequences were inserted into the 3′ end of their open reading frame of *TDH3* in DSY655 through homologous recombination [11]. The obtained strain *TDH3-GFP*-DSY654 with corrected GFP insertion was checked by PCR diagnosis.

Broth microdilution assay. Minimum inhibitory concentrations (MICs) of the tested agents against *Candida* species were determined by the broth microdilution procedure according to the CLSI M27-A3 guidelines [12]. In brief, 100 μL of the RPMI1640 medium containing 2-5 × 10^3 cfu/mL overnight-grown *Candida* yeast phase cells were dispensed into the wells of 96-well flat bottom microtiter plates. Twofold dilutions of the test compounds were incorporated into these wells. The plates were incubated at 35 °C for 48 h and observed visually for growth inhibition. MIC was determined as the lowest concentration required for complete growth inhibition (no visible growth).

To determine minimum fungicidal concentrations (MFCs), 10 μL cultures from wells at the MIC and concentrations above the MIC were plated on YPD agar plates and incubated at 30 °C for 48 h, and the number of colonies appearing on the plates was counted and compared with that appearing on the control plates (without any test compound). MFC was considered the lowest concentration killing 99.9 % of the inoculums.

***C. elegans–C. albicans* assay.** The *C. elegans–C. albicans* assay was performed according to previous reported method [13,14]. Specifically, nematodes were grown on nematode growth medium (NGM) with *Escherichia coli* strain OP50 as the food source. L4-adult stage worms were washed with M9 buffer and placed on 48 h-old *C. albicans* lawns (on BHI agar plates) for 2 h. The infected worms were washed off the plates with screen medium that contained 80% M9 buffer,

20% BHI, 10 μg/ml cholesterol and 90 μg/ml kanamycin and re-suspended at a density of 1–2 worm/μL in screen medium. 20 μL of the suspension of pre-infected worms were added to wells of 96-well plates. 80 μL of screen medium containing IS-2-Pi-TPP was dispensed into the indicated well. The plates were incubated at 25 °C. The survival was monitored and recorded each day based on nematode shape. After 5 days, the plates were imaged using an Olympus microscope equipped with a 4 × magnification objective lens.

To determine the toxicity of IS-2-Pi-TPP to *C. elegans*, healthy nematodes were incubated with serial concentrations of IS-2-Pi-TPP for 5 days, and the survival state was monitored by microscopic observation.

Chemistry. All commercially available reagents were used without further purification. Anhydrous solvents were dried through routine protocols. All reactions were carried out under a nitrogen atmosphere in dry glassware with magnetic stirring. Column chromatography was conducted on 200-300 mesh silica gel (Qingdao Haiyang Chemical, China). Analytical TLC was carried out by employing 0.25 mm silica gel plates (GF254) and visualization under UV light. NMR spectra were recorded on a Bruker 400 (1H, 400; 13C, 101 MHz) or Bruker 600 (1H, 600 MHz; 13C, 150 MHz) spectrometer. Chemical shifts were expressed in ppm, and J values were given in Hz. High-resolution mass spectra were measured using a Thermo Fisher Finnigan LTQ Orbitrap Elite mass spectrometer. Ionization was achieved in positive mode. The purity of the final compounds was verified using an HPLC system (Agilent Technologies 1200) equipped with a G1311A isopump, a G1322A degasser, and a G1315D DAD detector using an Eclipse XDB-C18 column (150 mm × 4.6 mm, 5 μm). All compounds evaluated for their biological effects were > 95 % pure.

(4-Carboxybutyl)triphenylphosphonium bromide (1). To a stirred solution of triphenylphosphine (1.0 g, 3.81 mmol) in anhydrous toluene (30 mL) was added 5-bromovaleric acid (690 mg, 3.81 mmol). Then, the mixture was allowed to stir under reflux for 12 h. After cooling to room temperature, the white precipitate was filtered and washed with an additional 20 mL of toluene. Next, the solvent was evaporated under reduced pressure to afford compound **1**, which was used directly without further purification.

(5-(((1*S*,2*S*,4*S*,5*R*)-5-(Cinnamoyloxy)-4,7,7-trimethylbicyclo[2.2.1]heptan-2-yl)oxy)-5-oxopentyl)triphenylphosphonium bromide (2). To a stirred solution of

(1S,2R,4S,5S)-5-hydroxy-1,7,7-trimethylbicyclo[2.2.1]heptan-2-yl cinnamate (12 mg, 0.04 mmol) in DMF (2 mL) were added compound **1** (27 mg, 0.06 mmol), EDCI (12 mg, 0.06 mmol) and DMAP (10 mg, 0.08 mmol). The reaction mixture was then stirred overnight at room temperature, after which TLC analysis indicated the consumption of the starting material. Next, 5 mL of water was added to the resulting solution, which was then extracted with CH_2Cl_2 (10 mL × 2). The combined extracts were washed with brine, dried over $MgSO_4$, and evaporated under vacuum to obtain the crude product, which was purified by flash chromatography to provide compound **2** (17 mg, 59 %) as a colorless oil. ^{1}H NMR (600 MHz, DMSO) δ 7.94 – 7.88 (m, 3H), 7.83 – 7.71 (m, 15H), 7.65 (d, *J* = 16.0 Hz, 1H), 7.43 (dd, *J* = 5.0, 1.7 Hz, 2H), 6.68 (d, *J* = 16.1 Hz, 1H), 4.91 – 4.83 (m, 1H), 4.56 (dd, *J* = 8.0, 3.5 Hz, 1H), 3.67 – 3.59 (m, 2H), 3.52 – 3.48 (m, 2H), 2.46 – 2.38 (m, 2H), 2.35 (t, *J* = 7.5 Hz, 2H), 1.78 (d, *J* = 5.1 Hz, 1H), 1.75 – 1.68 (m, 2H), 1.59 – 1.51 (m, 2H), 1.45 – 1.40 (m, 1H), 1.20 – 1.15 (m, 1H), 0.94 (s, 3H), 0.88 (s, 3H), 0.85 (s, 3H). ^{13}C NMR (151 MHz, DMSO) δ 172.0, 166.4, 144.6, 135.0, 134.0, 133.7, 133.6, 130.4, 130.3, 129.0, 128.5, 118.8, 118.2, 77.2, 76.4, 69.8, 49.3, 47.3, 40.1, 33.5, 32.9, 20.4, 19.3, 12.7. HR-ESI-MS *m/z* calculated for $C_{42}H_{46}O_4P^+$ [M - Br]$^+$ 645.3128, found 645.3130.

(5-Oxo-5-(((1*R*,2*S*,4*R*)-1,7,7-trimethylbicyclo[2.2.1]heptan-2-yl)oxy)pentyl)triphenylphosphonium bromide (3). The general procedure for the synthesis of **3** was similar to that used for **2** and afforded the product as a colorless oil. ^{1}H NMR (400 MHz, MeOD) δ 7.94 – 7.73 (m, 15H), 4.85 – 4.81 (m, 1H), 3.53 – 3.41 (m, 2H), 2.43 (t, *J* = 7.1 Hz, 2H), 2.36 – 2.25 (m, 1H), 1.96 – 1.83 (m, 3H), 1.81 – 1.69 (m, 3H), 1.65 (t, *J* = 4.5 Hz, 1H), 1.36 – 1.25 (m, 1H), 1.21 – 1.10 (m, 1H), 0.91 (s, 3H), 0.89 (s, 3H), 0.85 (d, *J* = 3.5 Hz, 1H), 0.77 (s, 3H). ^{13}C NMR (101 MHz, MeOD) δ 175.0, 136.3 (d, *J* = 3.0 Hz), 134.8 (d, *J* = 10.0 Hz), 131.6 (d, *J* = 12.6 Hz), 119.8 (d, *J* = 86.4 Hz), 81.3, 46.2, 37.8, 34.4, 28.9, 28.1, 27.0, 26.8, 23.1, 22.7, 22.2, 20.0, 19.1, 13.9. HR-ESI-MS *m/z* calculated for $C_{33}H_{40}O_2P^+$ [M - Br]$^+$ 499.2760, found 499.2761.

(4*R*,6a*R*,9*S*,11b*S*)-4,9,11b-Trimethyl-8-oxotetradecahydro-6a,9-methanocyclohepta[a]naphthalene-4-carboxylic acid (5). Isosteviol (**5**) was obtained by the hydrolysis of stevioside (**4**, 4 g) with 30 mL of 10 % sulfuric acid at 95 °C for 5 h. After cooling to room temperature, the reaction mixture was filtered and washed with saturated $NaHCO_3$ aqueous solution and H_2O. The crude product was recrystallized from ethanol as colorless crystals in 90 % yield. ^{1}H NMR (400 MHz, $CDCl_3$) δ 2.63 (dd, *J* = 18.6, 2.4 Hz, 1H), 2.15 (d, *J* = 13.3 Hz, 1H), 1.90 – 1.53 (m, 9H), 1.49 (dd,

J = 13.6, 2.9 Hz, 1H), 1.45 – 1.32 (m, 3H), 1.24 (s, 3H), 1.22 – 1.11 (m, 3H), 1.07 – 1.00 (m, 1H), 0.97 (s, 3H), 0.95 – 0.85 (m, 1H), 0.77 (s, 3H). ^{13}C NMR (101 MHz, $CDCl_3$) δ 183.8, 57.1, 54.8, 54.4, 48.9, 48.6, 43.8, 41.6, 39.9, 39.6, 38.3, 37.8, 37.4, 29.1, 21.7, 20.5, 20.0, 19.0, 13.4. ESI-MS *m/z* 317 $[M - H]^-$.

(4*R*,6a*S*,7*R*,8*R*,9*S*,11b*S*)-8-Hydroxy-7-(hydroxymethyl)-4,9,11b-trimethyltetradecahydro-6a,9-methanocyclohepta[*a*]naphthalene-4-carboxylic acid (6). To a stirred solution of **5** (0.5 g, 1.65 mmol) in ethanol (4 mL) were added sodium hydrate solution (330 mg in 2 mL of water, 8.25 mmol) and 37 % HCHO aqueous solution (1.15 mL, 11.55 mmol) at ambient temperature. Then, a reflux condenser was fitted to the flask, and the temperature was raised to 75 °C for 5 h before pouring the reaction mixture into ice water. The mixture was acidified with 5 % dilute hydrochloric acid afforded a white precipitate, which was then recovered through filtration. The cake was washed repeatedly with water. The crude residue was purified by recrystallization in methanol to afford compound **6** (495 mg, 90 %). ^1H NMR (400 MHz, MeOD) δ 3.81 (dd, J = 10.6, 5.2 Hz, 1H), 3.54 (d, J = 4.7 Hz, 1H), 3.46 (dd, J = 10.5, 8.7 Hz, 1H), 3.32 (s, 2H), 2.09 (d, J = 13.2 Hz, 1H), 2.00 (ddd, J = 8.3, 5.2, 3.3 Hz, 1H), 1.94 – 1.80 (m, 2H), 1.80 – 1.61 (m, 5H), 1.59 – 1.51 (m, 1H), 1.42 – 1.32 (m, 2H), 1.15 (s, 3H), 1.14 – 1.06 (m, 2H), 1.05 – 0.92 (m, 3H), 0.88 (s, 3H), 0.86 (s, 3H). ^{13}C NMR (101 MHz, MeOD) δ 181.7, 86.0, 64.4, 59.3, 58.4, 55.6, 51.2, 44.6, 43.9, 41.9, 41.0, 39.5, 39.2, 36.1, 34.6, 29.6, 25.6, 23.5, 20.6, 20.1, 13.9. ESI-MS *m/z* 349 $[M - H]^-$.

Benzyl(4*R*,6a*S*,7*R*,8*R*,9*S*,11b*S*)-8-hydroxy-7-(hydroxymethyl)-4,9,11b-trimethyltetradecahydro-6a,9-methanocyclohepta[a]naphthalene-4-carboxylate (7). To a stirred solution of **6** (240 mg, 0.69 mmol) in DMF (3 mL) were added potassium carbonate (286 mg, 2.07 mmol), benzyl bromide (164 μL, 1.38 mmol) and a catalytic amount of potassium iodide (23 mg, 0.138 mmol). The reaction mixture was stirred at room temperature overnight. Then, 20 mL of EtOAc was added to the resulting solution, which was washed with water and brine, dried over anhydrous sodium sulfate, filtered, and concentrated under reduced pressure. The residue was purified by column chromatography to provide compound **7** (296 mg, 98 %) as a colorless oil. ^1H NMR (400 MHz, $CDCl_3$) δ 7.42 – 7.29 (m, 5H), 5.12 (d, *J* = 12.3 Hz, 1H), 5.01 (d, *J* = 12.3 Hz, 1H), 3.83 (dd, *J* = 9.8, 4.9 Hz, 1H), 3.61 (d, *J* = 4.7 Hz, 1H), 3.40 (t, *J* = 10.4 Hz, 1H), 2.19 (d, *J* = 13.4 Hz, 1H), 1.96 – 1.89 (m, 1H), 1.84 – 1.48 (m, 10H), 1.45 – 1.38 (m, 1H), 1.35 (dd, *J* = 11.8, 2.7 Hz, 1H),

1.18 (s, 3H), 1.09 – 0.93 (m, 4H), 0.92 (s, 3H), 0.85 (td, J = 13.5, 4.6 Hz, 1H), 0.68 (s, 3H). ^{13}C NMR (101 MHz, $CDCl_3$) δ 177.3, 136.2, 128.6, 128.2, 86.9, 66.3, 65.0, 57.7, 57.3, 54.3, 50.5, 44.0, 42.6, 41.0, 39.7, 38.4, 38.2, 34.9, 33.2, 29.0, 25.1, 22.4, 19.7, 19.0, 13.3. ESI-MS *m/z* 441 $[M + H]^+$.

Benzyl(4*R*,6a*S*,7*R*,8*R*,9*S*,11b*S*)-8-hydroxy-4,9,11b-trimethyl-7-((tosyloxy)methyl)tetradecahydro-6a,9-methanocyclohepta[a]naphthalene-4-carboxylate (8). The benzyl ester of **7** (167 mg, 0.38 mmol) was dissolved in 2 mL of anhydrous pyridine, then P-toluene sulfochloride (87 mg, 0.456 mmol) and 4-dimethylaminopyridine (46 mg, 0.38 mmol) was added. The mixture was stirred at room temperature overnight. Then, 20 mL of EtOAc was added to the resulting solution, which was washed with water and brine, dried over anhydrous sodium sulfate, filtered, and concentrated under reduced pressure. The crude product was purified by flash chromatography to give the compound **8** (162 mg, 72 %) as an oil liquid. ^{1}H NMR (400 MHz, $CDCl_3$) δ 7.80 (d, J = 8.3 Hz, 2H), 7.39 – 7.29 (m, 7H), 5.07 (s, 2H), 4.26 (dd, J = 9.5, 4.9 Hz, 1H), 3.94 (t, J = 9.6 Hz, 1H), 3.41 (t, J = 4.2 Hz, 1H), 2.44 (s, 3H), 2.22 – 2.14 (m, 1H), 2.13 – 2.06 (m, 1H), 1.78 – 1.64 (m, 5H), 1.59 – 1.43 (m, 4H), 1.42 – 1.35 (m, 1H), 1.29 (dd, J = 11.9, 2.5 Hz, 1H), 1.15 (s, 3H), 1.05 – 0.99 (m, 2H), 0.98 – 0.89 (m, 3H), 0.85 (s, 3H), 0.80 (dd, J = 13.6, 4.5 Hz, 1H), 0.57 (s, 3H). ^{13}C NMR (101 MHz, $CDCl_3$) δ 177.1, 145.0, 136.1, 133.3, 130.1, 128.7, 128.6, 128.3, 128.0, 84.9, 72.8, 66.2, 57.6, 57.1, 54.0, 47.8, 43.9, 43.0, 41.0, 39.7, 38.2, 38.1, 34.8, 33.2, 29.0, 25.0, 22.1, 21.8, 19.5, 18.9, 13.1. ESI-MS *m/z* 595 $[M + H]^+$.

Benzyl(4*R*,6a*S*,7*R*,9*S*,11b*S*)-4,9,11b-trimethyl-8-oxo-7-((tosyloxy)methyl)tetradecahydro-6a,9-methanocyclohepta[a]naphthalene-4-carboxylate (9). To a stirred solution of **8** (71 mg, 0.12 mmol) in dry CH_2Cl_2 (3 mL) was added pyridinium chlorochromate (52 mg, 0.24 mmol), and the mixture was stirred for 3 h at room temperature. Then, 20 mL of CH_2Cl_2 was added to the resulting solution, which was washed with H_2O and brine, dried over anhydrous sodium sulfate, filtered, and concentrated under reduced pressure. The crude product was purified by flash chromatography to afford compound **9** (68 mg, 96 %). ^{1}H NMR (400 MHz, $CDCl_3$) δ 7.72 (d, J = 8.2 Hz, 2H), 7.40 – 7.31 (m, 7H), 5.13 (d, J = 12.1 Hz, 1H), 5.03 (d, J = 12.0 Hz, 1H), 4.15 (dd, J = 9.7, 4.7 Hz, 1H), 4.07 (dd, J = 9.7, 2.2 Hz, 1H), 2.45 (s, 3H), 2.38 – 2.33 (m, 1H), 2.19 (d, J = 13.3 Hz, 1H), 1.98 – 1.90 (m, 1H), 1.88 – 1.81 (m, 1H), 1.77 (dd, J = 11.7, 2.5 Hz, 1H), 1.66 – 1.52 (m, 5H), 1.42 – 1.32 (m, 2H), 1.24 (dd, J = 12.0, 2.0 Hz, 2H), 1.20 (s, 3H), 1.16 – 1.12 (m,

2H), 1.10 – 1.02 (m, 1H), 0.99 (dd, *J* = 13.5, 4.1 Hz, 1H), 0.88 (s, 3H), 0.87 – 0.81 (m, 1H), 0.47 (s, 3H). ^{13}C NMR (101 MHz, $CDCl_3$) δ 177.0, 145.1, 136.1, 132.4, 130.0, 128.8, 128.6, 128.4, 128.2, 125.2, 67.6, 66.2, 57.1, 57.0, 52.9, 51.2, 48.1, 43.9, 40.5, 39.7, 38.4, 38.0, 37.2, 35.1, 28.9, 21.8, 21.7, 19.6, 18.9, 13.2. ESI-MS *m/z* 593 $[M + H]^+$.

(4*R*,6a*S*,7*R*,9*S*,11b*S*)-4,9,11b-Trimethyl-8-oxo-7-((tosyloxy)methyl)tetradecahydro-6a,9-methanocyclohepta[a]naphthalene-4-carboxylic acid (10). To a solution of **9** (66 mg, 0.12 mmol) in anhydrous ethanol (10 mL) was added 13 mg of 10 % Pd-C, and the mixture was stirred under a hydrogen atmosphere at room temperature for 18 h. After removing Pd-C by filtration, the solvent was removed under reduced pressure to give the crude product, which was purified by flash chromatography to provide compound **10** (53 mg, 95 %). ^{1}H NMR (400 MHz, $CDCl_3$) δ 7.73 (d, *J* = 8.2 Hz, 2H), 7.35 (d, *J* = 8.2 Hz, 2H), 4.29 (dd, *J* = 9.7, 2.4 Hz, 1H), 4.24 (dd, *J* = 9.7, 4.7 Hz, 1H), 2.52 – 2.45 (m, 1H), 2.44 (s, 3H), 2.16 (d, *J* = 13.3 Hz, 1H), 2.03 (d, *J* = 14.2 Hz, 1H), 1.91 – 1.55 (m, 7H), 1.47 – 1.36 (m, 2H), 1.33 – 1.25 (m, 3H), 1.25 (s, 3H), 1.21 – 1.11 (m, 3H), 1.02 (td, *J* = 13.4, 3.8 Hz, 1H), 0.91 (s, 3H), 0.68 (s, 3H). ^{13}C NMR (101 MHz, $CDCl_3$) δ 145.1, 132.3, 130.0, 128.2, 67.8, 57.1, 57.0, 53.0, 51.3, 48.2, 43.7, 40.6, 39.7, 38.5, 37.8, 37.2, 35.1, 29.0, 21.8, 21.5, 19.7, 19.6, 18.9, 13.3. ESI-MS *m/z* 501 $[M - H]^-$.

(4*R*,6a*S*,9*S*,11b*S*)-4,9,11b-Trimethyl-7-methylene-8-oxotetradecahydro-6a,9-methanocyclohepta[a]naphthalene-4-carboxylic acid (11). Compound **10** (50 mg, 0.10 mmol) and 4-dimethylaminopyridine (18 mg, 0.14 mmol) were dissolved in anhydrous pyridine (3 mL), and then the mixture was allowed to stir under reflux for 4 h. Upon cooling to room temperature, 20 mL of EtOAc was added to the mixture, which was then washed with water and brine, dried over anhydrous sodium sulfate, and filtered, and the solvent was evaporated *in vacuo* to give the crude product. The crude product was purified by flash chromatography to afford compound **11** (28 mg, 74 %) as a white solid. ^{1}H NMR (400 MHz, $CDCl_3$) δ 6.06 (s, 1H), 5.49 (s, 1H), 2.23 - 2.05 (m, 2H), 2.03 - 1.93 (m, 2H), 1.85 - 1.66 (m, 4H), 1.56 - 1.38 (m, 5H), 1.27 (s, 3H), 1.26 - 1.19 (m, 3H), 1.05 (dd, J = 13.5, 4.0 Hz, 1H), 1.01 (s, 3H), 0.86 (td, J = 12.9, 4.1 Hz, 1H), 0.68 (s, 3H). ^{13}C NMR (101 MHz, $CDCl_3$) δ 210.9, 183.4, 154.5, 116.3, 57.1, 56.7, 53.6, 46.9, 43.9, 43.9, 40.5, 38.9, 38.2, 38.0, 29.2, 21.9, 21.2, 20.3, 19.0, 12.5. ESI-MS *m/z* 329 $[M - H]^-$.

(3-Bromopropyl)triphenylphosphonium bromide (12). Triphenylphosphine (1.0 g, 3.81 mmol) and 1,3-dibromopropane (0.39 mL, 3.81 mmol) were dissolved in anhydrous toluene (20 mL) at

room temperature, and then the mixture was allowed to stir under reflux for 12 h. The white precipitate was filtered, washed with an additional 20 mL of toluene and dried overnight in a vacuum oven, and the product was used directly without further purification.

(4-Bromobutyl)triphenylphosphonium bromide (13) and (10-Bromodecyl)triphenylphosphonium bromide (14). The general procedure for the synthesis of **13** and **14** was similar to that used for **12**.

Methyl(4*R*,6a*R*,9*S*,11b*S*)-4,9,11b-trimethyl-8-oxotetradecahydro-6a,9-methanocyclohepta[a] naphthalene-4-carboxylate (15). To a solution of compound **5** (200 mg, 0.66 mmol) in 5 mL of DMF were added anhydrous K_2CO_3 (228 mg, 1.65 mmol) and MeI (204 μL, 3.3 mmol). The mixture was stirred for 4 h at room temperature. Next, 5 mL of water was added to the resulting solution, which was extracted with EtOAc (10 mL × 2). The combined extracts were washed with brine, dried over $MgSO_4$, and evaporated under vacuum to obtain the crude product, which was then purified by flash chromatography to afford compound **15** (204 mg, 98 %) as a white solid, mp 202-204 °C. ^{1}H NMR (400 MHz, $CDCl_3$) δ 3.63 (s, 3H), 2.62 (dd, *J* = 18.6, 3.7 Hz, 1H), 2.17 (d, *J* = 13.4 Hz, 1H), 1.88 (dd, *J* = 13.8, 2.4 Hz, 1H), 1.83 – 1.74 (m, 2H), 1.74 – 1.31 (m, 11H), 1.28 – 1.20 (m, 1H), 1.18 (s, 3H), 1.12 (d, *J* = 11.7 Hz, 1H), 1.03 (dd, *J* = 13.4, 4.1 Hz, 1H), 0.97 (s, 3H), 0.90 (td, *J* = 13.2, 4.1 Hz, 1H), 0.68 (s, 3H). ^{13}C NMR (101 MHz, $CDCl_3$) δ 178.0, 57.2, 54.9, 54.4, 51.4, 48.8, 48.6, 43.9, 41.6, 39.9, 39.6, 38.1, 37.4, 29.0, 21.8, 20.5, 20.0, 19.1, 13.3. ESI-MS *m/z* 333 $[M + H]^+$.

Butyl(4*R*,4a*S*,6a*R*,9*S*,11b*S*)-4,9,11b-trimethyl-8-oxotetradecahydro-6a,9-methanocyclohepta[a]naphthalene-4-carboxylate (16). The general procedure for the synthesis of **16** was similar to that used for **15** and provided the product as a colorless oil. ^{1}H NMR (600 MHz, $CDCl_3$) δ 4.09 – 3.96 (m, 2H), 2.63 (dd, *J* = 18.6, 3.7 Hz, 1H), 2.18 (d, *J* = 13.3 Hz, 1H), 1.89 (dd, *J* = 14.0, 2.0 Hz, 1H), 1.83 – 1.76 (m, 2H), 1.73 – 1.68 (m, 3H), 1.67 – 1.63 (m, 1H), 1.62 – 1.58 (m, 3H), 1.54 (dd, *J* = 11.6, 2.4 Hz, 1H), 1.48 (dd, *J* = 13.5, 3.6 Hz, 1H), 1.45 – 1.37 (m, 5H), 1.26 – 1.20 (m, 1H), 1.18 (s, 3H), 1.12 (d, *J* = 12.0 Hz, 1H), 1.01 (dd, *J* = 13.5, 4.1 Hz, 1H), 0.97 (s, 3H), 0.95 (d, *J* = 5.5 Hz, 1H), 0.93 (t, *J* = 6.5 Hz, 3H), 0.89 (dd, *J* = 13.2, 4.0 Hz, 1H), 0.71 (s, 3H). ^{13}C NMR (151 MHz, $CDCl_3$) δ 177.4, 64.1, 57.1, 54.7, 54.3, 48.7, 48.4, 43.8, 41.6, 39.9, 39.5, 38.0, 38.0, 37.3, 30.6, 29.0, 21.7, 20.3, 19.9, 19.4, 19.0, 13.7, 13.4. ESI-MS *m/z* 375 $[M + H]^+$.

Triphenyl(3-(((4*R*,6a*R*,9*S*,11b*S*)-4,9,11b-trimethyl-8-oxotetradecahydro-6a,9-methanocycloh epta[a]naphthalene-4-carbonyl)oxy)propyl)phosphonium bromide (17). To a solution of compound **5** (50 mg, 0.16 mmol) in 3 mL of anhydrous DMF were added K_2CO_3 (45 mg, 0.32 mmol) and compound **12** (112 mg, 0.24 mmol). The mixture was stirred overnight at room temperature. After completion of the reaction, 5 mL of water was added to the resulting solution, which was then extracted with EtOAc (10 mL × 2). The combined extracts were washed with brine, dried over $MgSO_4$, and evaporated under vacuum to obtain the crude product, which was then purified by flash chromatography to afford compound **17** (102 mg, 93 %) as a colorless oil. ^{1}H NMR (400 MHz, MeOD) δ 7.97 – 7.89 (m, 3H), 7.87 – 7.74 (m, 12H), 4.33 – 4.10 (m, 2H), 3.48 – 3.42 (m, 2H), 2.46 (dd, *J* = 18.4, 3.7 Hz, 1H), 2.14 (d, *J* = 14.0 Hz, 1H), 2.08 – 1.96 (m, 2H), 1.92 – 1.85 (m, 1H), 1.75 – 1.64 (m, 3H), 1.62 – 1.49 (m, 4H), 1.48 – 1.42 (m, 2H), 1.38 – 1.28 (m, 6H), 1.26 – 1.22 (m, 1H), 1.18 (s, 3H), 1.15 – 1.06 (m, 1H), 0.95 (s, 3H), 0.61 (s, 3H). ^{13}C NMR (101 MHz, MeOD) δ 178.6, 136.6, 136.6, 134.9, 134.8, 131.8, 131.6, 119.9, 119.0, 65.3, 58.0, 55.6, 55.0, 45.0, 42.3, 40.7, 40.7, 39.1, 38.9, 38.4, 31.3, 30.3, 29.2, 24.4, 23.2, 23.2, 23.1, 21.4, 20.2, 20.0, 14.1. HR-ESI-MS *m/z* calculated for $C_{41}H_{50}O_3P^+$ [M - Br]$^+$ 621.3492, found 621.3511.

Triphenyl(4-(((4*R*,6a*R*,9*S*,11b*S*)-4,9,11b-trimethyl-8-oxotetradecahydro-6a,9-methanocycloh epta[a]naphthalene-4-carbonyl)oxy)butyl)phosphonium bromide (18). The general procedure for the synthesis of **18** was similar to that used for **17** and provided the product as a colorless oil. ^{1}H NMR (400 MHz, MeOD) δ 7.96 – 7.70 (m, 15H), 4.15 (dt, *J* = 12.0, 6.1 Hz, 1H), 4.02 (dt, *J* = 11.5, 5.9 Hz, 1H), 3.56 – 3.43 (m, 2H), 2.49 (dd, *J* = 18.5, 3.7 Hz, 1H), 2.09 – 2.00 (m, 1H), 1.97 – 1.87 (m, 2H), 1.82 – 1.63 (m, 7H), 1.62 – 1.49 (m, 4H), 1.48 – 1.41 (m, 2H), 1.37 – 1.27 (m, 3H), 1.27 – 1.13 (m, 3H), 1.07 (s, 3H), 1.06 – 1.01 (m, 1H), 0.94 (s, 3H), 0.62 (s, 3H). ^{13}C NMR (101 MHz, MeOD) δ 178.8, 136.4, 136.4, 134.8, 134.7, 131.7, 131.6, 120.2, 119.4, 64.1, 58.0, 55.6, 55.0, 45.0, 42.3, 40.7, 40.6, 39.1, 38.8, 38.4, 30.7, 30.5, 29.4, 22.9, 22.6, 22.1, 21.4, 20.8, 20.8, 20.2, 20.1, 14.0. HR-ESI-MS *m/z* calculated for $C_{42}H_{52}O_3P^+$ [M - Br]$^+$ 635.3649, found 635.3651.

Methyl(4*R*,6a*S*,9*S*,11b*S*)-4,9,11b-trimethyl-7-methylene-8-oxotetradecahydro-6a,9-methanoc yclohepta[a]naphthalene-4-carboxylate (19). The general procedure for the synthesis of **19** was similar to that used for **15**, and the product was obtained as a white solid, mp 190-192 °C. ^{1}H

NMR (400 MHz, $CDCl_3$) δ 6.05 (s, 1H), 5.46 (s, 1H), 3.63 (s, 3H), 2.18 (d, *J* = 13.3 Hz, 1H), 2.12 – 1.93 (m, 3H), 1.84 – 1.64 (m, 4H), 1.56 – 1.37 (m, 5H), 1.29 (dd, *J* = 12.9, 3.5 Hz, 1H), 1.21 (s, 3H), 1.20 – 1.15 (m, 2H), 1.05 (dd, *J* = 13.5, 4.0 Hz, 1H), 1.01 (s, 3H), 0.86 (td, *J* = 13.1, 4.0 Hz, 1H), 0.58 (s, 3H). ^{13}C NMR (101 MHz, $CDCl_3$) δ 210.9, 177.9, 154.6, 116.1, 57.0, 56.8, 53.6, 51.4, 46.9, 44.0, 43.9, 40.5, 38.7, 38.3, 38.1, 38.0, 29.1, 21.9, 21.1, 20.3, 19.1, 12.4. ESI-MS *m/z* 345 $[M + H]^+$.

Butyl(4*R*,4a*S*,6a*S*,9*S*,11b*S*)-4,9,11b-trimethyl-7-methylene-8-oxotetradecahydro-6a,9-methanocyclohepta[a]naphthalene-4-carboxylate (20). The general procedure for the synthesis of **20** was similar to that used for **16** and provided the product as a colorless oil. ^1H NMR (600 MHz, $CDCl_3$) δ 6.05 (s, 1H), 5.44 (s, 1H), 4.10 – 3.93 (m, 2H), 2.19 (d, *J* = 13.3 Hz, 1H), 2.13 – 2.04 (m, 1H), 2.02 – 1.94 (m, 2H), 1.82 – 1.65 (m, 4H), 1.63 – 1.58 (m, 2H), 1.53 – 1.39 (m, 7H), 1.30 (dd, *J* = 12.9, 3.9 Hz, 1H), 1.21 (s, 3H), 1.18 (d, *J* = 13.3 Hz, 1H), 1.06 – 0.99 (m, 4H), 0.94 (t, *J* = 7.4 Hz, 3H), 0.86 (td, *J* = 13.3, 4.0 Hz, 1H), 0.61 (s, 3H). ^{13}C NMR (151 MHz, $CDCl_3$) δ 210.9, 177.5, 154.7, 115.9, 64.2, 57.0, 56.8, 53.6, 46.9, 44.1, 43.9, 40.6, 38.8, 38.3, 38.2, 38.0, 30.7, 29.2, 22.0, 21.2, 20.3, 19.6, 19.2, 13.8, 12.5. ESI-MS *m/z* 387 $[M + H]^+$.

Triphenyl(3-(((4*R*,6a*S*,9*S*,11b*S*)-4,9,11b-trimethyl-7-methylene-8-oxotetradecahydro-6a,9-methanocyclohepta[a]naphthalene-4-carbonyl)oxy)propyl)phosphonium bromide (21). To a solution of compound **11** (30 mg, 0.10 mmol) in 2.0 mL of anhydrous DMF were added K_2CO_3 (28 mg, 0.18 mmol) and compound **9** (70 mg, 0.14 mmol). The mixture was stirred overnight at room temperature. After completion of the reaction, 5 mL of water was added to the resulting solution, which was then extracted with EtOAc (10 mL × 2). The combined extracts were washed with brine, dried over $MgSO_4$, and evaporated under vacuum to obtain the crude product, which was then purified by flash chromatography to afford compound **12** (64 mg, 98 %) as a colorless oil. ^1H NMR (400 MHz, MeOD) δ 7.96 – 7.89 (m, 3H), 7.87 – 7.74 (m, 12H), 5.86 (s, 1H), 5.27 (s, 1H), 4.34 – 4.10 (m, 2H), 3.60 – 3.42 (m, 2H), 2.18 – 2.11 (m, 1H), 2.08 – 1.97 (m, 4H), 1.92 (dt, *J* = 13.5, 3.1 Hz, 1H), 1.79 – 1.63 (m, 4H), 1.62 – 1.50 (m, 3H), 1.45 – 1.36 (m, 3H), 1.32 – 1.26 (m, 2H), 1.22 (s, 3H), 1.16 – 1.05 (m, 2H), 0.98 (s, 3H), 0.52 (s, 3H). ^{13}C NMR (101 MHz, MeOD) δ 212.7, 178.5, 156.5, 136.6, 136.5, 134.9, 134.8, 131.8, 131.6, 119.9, 119.0, 116.2, 64.6, 64.4, 57.7, 57.6, 54.3, 47.9, 45.1, 45.1, 41.3, 39.8, 39.0, 38.9, 29.3, 23.3, 23.2, 23.1, 22.1, 20.4, 20.1, 19.9, 13.2. HR-ESI-MS *m/z* calculated for $C_{42}H_{50}O_3P^+$ $[M - Br]^+$ 633.3492, found 633.3496.

Triphenyl(4-(((4*R*,6a*S*,9*S*,11b*S*)-4,9,11b-trimethyl-7-methylene-8-oxotetradecahydro-6a,9-methanocyclohepta[a]naphthalene-4-carbonyl)oxy)butyl)phosphonium bromide (22). The general procedure for the synthesis of **22** and **23** was similar to that used for **21**. Compound **22** was a white solid, mp 120-122 °C. ^{1}H NMR (400 MHz, MeOD) δ 7.94 – 7.71 (m, 15H), 5.95 (s, 1H), 5.38 (s, 1H), 4.15 (dt, *J* = 12.3, 6.2 Hz, 1H), 4.05 (dt, *J* = 11.4, 5.8 Hz, 1H), 3.55 – 3.42 (m, 2H), 2.09 – 1.99 (m, 2H), 1.98 – 1.85 (m, 4H), 1.82 – 1.48 (m, 9H), 1.41 – 1.28 (m, 4H), 1.24 (dd, *J* = 12.2, 2.2 Hz, 1H), 1.11 (s, 3H), 1.10 – 1.00 (m, 2H), 0.98 (s, 3H), 0.53 (s, 3H). ^{13}C NMR (101 MHz, MeOD) δ 212.7, 178.7, 156.4, 136.4, 136.4, 134.8, 134.7, 131.7, 131.6, 120.2, 119.4, 116.4, 64.1, 57.7, 57.6, 54.3, 47.9, 45.1, 45.1, 41.3, 39.7, 39.1, 38.9, 38.9, 30.8, 30.6, 29.4, 23.0, 22.2, 20.8, 20.7, 20.4, 20.2, 13.1. HR-ESI-MS *m/z* calculated for $C_{43}H_{52}O_3P^+$ $[M - Br]^+$ 647.3649, found 647.3658.

Triphenyl(10-(((4*R*,6a*S*,9*S*,11b*S*)-4,9,11b-trimethyl-7-methylene-8-oxotetradecahydro-6a,9-methanocyclohepta[a]naphthalene-4-carbonyl)oxy)decyl)phosphonium bromide (23). Compound **23** was a colorless oil. ^{1}H NMR (400 MHz, MeOD) δ 7.92 – 7.87 (m, 3H), 7.84 – 7.76 (m, 12H), 5.98 (s, 1H), 5.46 (d, *J* = 0.7 Hz, 1H), 4.15 – 3.95 (m, 2H), 3.48 – 3.37 (m, 2H), 2.20 – 2.10 (m, 2H), 2.04 – 1.92 (m, 2H), 1.82 – 1.52 (m, 16H), 1.43 – 1.30 (m, 12H), 1.21 (s, 3H), 1.18 – 1.08 (m, 2H), 0.97 (s, 3H), 0.63 (s, 3H). ^{13}C NMR (151 MHz, MeOD) δ 212.6, 179.1, 156.6, 136.3, 136.3, 134.8, 134.8, 131.6, 131.5, 120.3, 119.7, 116.2, 65.5, 57.8, 57.6, 54.3, 47.9, 45.1, 45.1, 41.5, 39.8, 39.1, 39.1, 38.9, 30.4, 30.3, 30.2, 29.9, 29.6, 29.4, 27.4, 23.6, 23.5, 23.2, 22.8, 22.5, 22.2, 20.5, 20.1, 13.2. HR-ESI-MS *m/z* calculated for $C_{49}H_{64}O_3P^+$ $[M - Br]^+$ 731.4588, found 731.4595.

2-Bromoethyl(4*R*,6a*S*,9*S*,11b*S*)-4,9,11b-trimethyl-7-methylene-8-oxotetradecahydro-6a,9-methanocyclohepta[a]naphthalene-4-carboxylate (24). To a 25 mL flame-dried round-bottomed flask containing compound **11** (510 mg, 1.6 mmol) in anhydrous DMF (10 mL) under nitrogen protection were added K_2CO_3 (443 mg, 3.2 mmol) and 1,2-dibromoethane (1.4 mL, 16 mmol). Then, the mixture was stirred for 4 h at room temperature. After TLC analysis indicated the consumption of the starting material, the reaction was quenched with water. The resulting mixture was extracted with CH_2Cl_2, and the extracts was washed with brine, dried over $MgSO_4$, filtered and concentrated under reduced pressure. The residue was purified by flash chromatography to obtain compound **24** as a white solid in quantitative yield, mp 87-89 °C.^{1}H NMR (400 MHz,

$CDCl_3$) δ 6.05 (s, 1H), 5.52 (s, 1H), 4.49 – 4.21 (m, 2H), 3.53 (t, J = 5.7 Hz, 2H), 2.26 – 2.07 (m, 2H), 2.05 – 1.93 (m, 2H), 1.88 – 1.65 (m, 4H), 1.56 – 1.37 (m, 5H), 1.30 (dd, J = 12.8, 3.6 Hz, 1H), 1.25 (s, 3H), 1.19 (ddd, J = 18.6, 12.7, 4.0 Hz, 2H), 1.07 (dd, J = 13.5, 4.0 Hz, 1H), 1.01 (s, 3H), 0.87 (td, J = 13.3, 4.1 Hz, 1H), 0.63 (s, 3H). ^{13}C NMR (101 MHz, $CDCl_3$) δ 210.9, 177.2, 154.5, 116.2, 64.2, 57.1, 56.8, 53.6, 46.9, 44.2, 43.9, 40.5, 38.8, 38.3, 38.2, 38.0, 29.2, 29.2, 22.0, 21.2, 20.3, 19.1, 12.6. ESI-MS *m/z* 438 [M + H]$^+$.

4-Bromobutyl(4*R*,6a*S*,9*S*,11b*S*)-4,9,11b-trimethyl-7-methylene-8-oxotetradecahydro-6a,9-methanocyclohepta[a]naphthalene-4-carboxylate (25). The general procedure for the synthesis of **25 - 27** was similar to that used for **24**. Compound **25** was a white solid, mp 72-74 °C. ^{1}H NMR (400 MHz, $CDCl_3$) δ 6.05 (s, 1H), 5.45 (s, 1H), 4.06 (t, J = 6.4 Hz, 2H), 3.44 (t, J = 6.6 Hz, 2H), 2.18 (d, J = 12.4 Hz, 1H), 2.10 – 1.92 (m, 5H), 1.86 – 1.65 (m, 6H), 1.60 (d, J = 3.0 Hz, 1H), 1.55 – 1.38 (m, 5H), 1.30 (dd, J = 12.9, 3.6 Hz, 1H), 1.22 (s, 3H), 1.18 (dd, J = 7.7, 5.3 Hz, 1H), 1.05 (dd, J = 13.5, 3.9 Hz, 1H), 1.01 (s, 3H), 0.86 (td, J = 12.8, 3.6 Hz, 1H), 0.61 (s, 3H). ^{13}C NMR (101 MHz, $CDCl_3$) δ 211.0, 177.5, 154.8, 116.2, 63.6, 57.2, 57.0, 53.7, 47.0, 44.3, 44.1, 40.7, 39.0, 38.4, 38.3, 38.1, 33.3, 29.9, 29.3, 27.6, 22.2, 21.3, 20.4, 19.3, 12.7. ESI-MS *m/z* 466 [M + H]$^+$.

8-Bromooctyl(4*R*,6a*S*,9*S*,11b*S*)-4,9,11b-trimethyl-7-methylene-8-oxotetradecahydro-6a,9-methanocyclohepta[a]naphthalene-4-carboxylate (26). Compound **26** was a colorless oil. ^{1}H NMR (400 MHz, $CDCl_3$) δ 6.05 (s, 1H), 5.44 (s, 1H), 4.02 (t, J = 6.6 Hz, 2H), 3.40 (t, J = 6.8 Hz, 2H), 2.19 (d, J = 13.2 Hz, 1H), 2.14 – 1.92 (m, 3H), 1.90 – 1.81 (m, 2H), 1.80 – 1.59 (m, 6H), 1.55 – 1.48 (m, 1H), 1.47 – 1.27 (m, 13H), 1.21 (s, 3H), 1.20 – 1.16 (m, 1H), 1.07 – 1.03 (m, 1H), 1.01 (s, 3H), 0.90 – 0.82 (m, 2H), 0.61 (s, 3H). ^{13}C NMR (101 MHz, $CDCl_3$) δ 210.9, 177.5, 154.7, 115.9, 64.4, 57.0, 56.8, 53.6, 46.9, 44.1, 43.9, 40.6, 38.8, 38.3, 38.2, 38.0, 34.1, 32.9, 29.2, 28.8, 28.6, 28.2, 26.3, 22.0, 21.2, 20.3, 19.2, 12.6. ESI-MS *m/z* 522 [M + H]$^+$.

10-Bromodecyl(4*R*,6a*S*,9*S*,11b*S*)-4,9,11b-trimethyl-7-methylene-8-oxotetradecahydro-6a,9-methanocyclohepta[a]naphthalene-4-carboxylate (27). Compound **27** was a colorless oil. ^{1}H NMR (400 MHz, $CDCl_3$) δ 6.04 (s, 1H), 5.44 (s, 1H), 4.02 (t, J = 6.5 Hz, 2H), 3.40 (t, J = 6.8 Hz, 2H), 2.18 (d, J = 13.3 Hz, 1H), 2.13 – 2.03 (m, 1H), 2.02 – 1.92 (m, 2H), 1.89 – 1.80 (m, 2H), 1.80 – 1.58 (m, 7H), 1.55 – 1.26 (m, 18H), 1.21 (s, 3H), 1.20 – 1.15 (m, 1H), 1.07 – 1.02 (m, 1H), 1.01 (s, 3H), 0.86 (td, J = 13.1, 4.0 Hz, 1H), 0.61 (s, 3H). ^{13}C NMR (101 MHz, $CDCl_3$) δ 210.9, 177.5, 154.7, 115.9, 64.5, 57.0, 56.8, 53.6, 46.9, 44.1, 43.9, 40.6, 38.8, 38.3, 38.2, 38.0, 34.2, 32.9,

29.5, 29.5, 29.3, 29.2, 28.9, 28.7, 28.3, 26.3, 22.0, 21.2, 20.3, 19.2, 12.6. ESI-MS *m/z* 550 $[M + H]^+$.

Triphenyl(8-(((4*R*,6a*S*,9*S*,11b*S*)-4,9,11b-trimethyl-7-methylene-8-oxotetradecahydro-6a,9-methanocyclohepta[a]naphthalene-4-carbonyl)oxy)octyl)phosphonium bromide (28). To a stirred solution of compound **26** (36 mg, 0.07 mmol) in dry toluene (4 mL) at room temperature, triphenylphosphine (54 mg, 0.21 mmol) was added, and then the mixture was allowed to stir under reflux for 48 h. After TLC analysis indicated the consumption of the starting material, the solvent was subsequently removed under reduced pressure, and the residue was purified by flash chromatography to afford compound **28** (23 mg, 43 %) as a colorless oil. ^{1}H NMR (600 MHz, MeOD) δ 7.92 – 7.86 (m, 3H), 7.83 – 7.73 (m, 12H), 5.96 (s, 1H), 5.44 (d, *J* = 0.6 Hz, 1H), 4.04 (dt, *J* = 11.0, 6.4 Hz, 1H), 3.99 (dt, *J* = 10.9, 6.5 Hz, 1H), 3.44 – 3.35 (m, 2H), 2.21 – 2.07 (m, 2H), 2.04 – 1.98 (m, 1H), 1.94 (dt, *J* = 13.6, 3.2 Hz, 1H), 1.83 – 1.72 (m, 2H), 1.70 – 1.61 (m, 6H), 1.60 – 1.54 (m, 4H), 1.42 – 1.36 (m, 6H), 1.34 – 1.27 (m, 6H), 1.21 (s, 3H), 1.18 – 1.05 (m, 2H), 0.99 (s, 3H), 0.61 (s, 3H). ^{13}C NMR (151 MHz, MeOD) δ 212.7, 179.1, 156.7, 136.3, 136.3, 134.8, 134.8, 131.6, 131.5, 120.3, 119.7, 116.2, 65.4, 57.8, 57.6, 54.3, 47.9, 45.2, 45.1, 41.5, 39.8, 39.1, 39.1, 38.9, 29.9, 29.8, 29.5, 29.3, 27.2, 23.5, 23.5, 23.2, 22.8, 22.5, 22.2, 20.4, 20.1, 13.2. HR-ESI-MS *m/z* calculated for $C_{47}H_{60}O_3P^+$ $[M - Br]^+$ 703.4275, found 703.4281.

2-(4-Methylpiperazin-1-yl)ethyl(4*R*,6a*S*,9*S*,11b*S*)-4,9,11b-trimethyl-7-methylene-8-oxotetradecahydro-6a,9-methanocyclohepta[a]naphthalene-4-carboxylate (29). To a stirred solution of compound **24** (50 mg, 0.12 mmol) in anhydrous DMF (3 mL) were added 1-methylpiperazine (40 μL, 0.36 mmol) and K_2CO_3 (34 mg, 0.24 mmol), and the mixture was stirred overnight at room temperature. After completion of the reaction, the mixture was quenched with water. Then, CH_2Cl_2 was added to the mixture, which was washed with brine, dried over $MgSO_4$, filtered, and concentrated under reduced pressure to provide product **29** as a colorless oil in quantitative yield. ^{1}H NMR (400 MHz, MeOD) δ 6.02 (s, 1H), 5.51 (s, 1H), 4.31 – 4.09 (m, 2H), 3.07 – 2.64 (m, 10H), 2.61 (s, 3H), 2.23 – 2.09 (m, 2H), 2.06 – 1.94 (m, 2H), 1.89 – 1.65 (m, 4H), 1.63 – 1.51 (m, 3H), 1.46 – 1.36 (m, 3H), 1.30 (dd, *J* = 12.1, 2.2 Hz, 1H), 1.24 (s, 3H), 1.22 – 1.06 (m, 2H), 0.98 (s, 3H), 0.94 (dd, *J* = 13.3, 4.1 Hz, 1H), 0.66 (s, 3H). ^{13}C NMR (101 MHz, MeOD) δ 212.8, 178.7, 156.5, 116.5, 62.3, 57.8, 57.7, 57.1, 55.3, 54.4, 52.4, 47.9, 45.1, 45.1, 44.6, 41.4, 39.8, 39.2, 39.0,

38.9, 29.4, 23.1, 22.2, 20.4, 20.1, 13.3. HR-ESI-MS *m/z* calculated for $C_{28}H_{45}N_2O_3$ $[M + H]^+$ 457.3385, found 457.3389.

2-(Piperazin-1-yl)ethyl(4*R*,6a*S*,9*S*,11b*S*)-4,9,11b-trimethyl-7-methylene-8-oxotetradecahydro-6a,9-methanocyclohepta[a]naphthalene-4-carboxylate (30). To a 25 mL flame-dried round-bottomed flask containing bromide **24** (100 mg, 0.23 mmol) in anhydrous DMF (5 mL) under nitrogen protection, K_2CO_3 (64 mg, 0.46 mmol) and piperazine (397 mg, 4.6 mmol) were added. The mixture was stirred at room temperature for 4 h, after which TLC analysis indicated the consumption of the starting material. Then, CH_2Cl_2 (30 mL) was poured into the mixture, which was washed with brine, dried over $MgSO_4$, filtered and concentrated under reduced pressure to give compound **30** as a colorless oil in quantitative yield. ^1H NMR (600 MHz, $CDCl_3$) δ 9.55 (s, 1H), 6.04 (s, 1H), 5.42 (s, 1H), 4.23 – 4.06 (m, 2H), 3.21 (t, *J* = 4.6 Hz, 4H), 2.90 – 2.76 (m, 4H), 2.74 – 2.65 (m, 2H), 2.14 (d, *J* = 13.2 Hz, 1H), 2.10 – 2.01 (m, 2H), 1.99 – 1.93 (m, 2H), 1.77 – 1.64 (m, 4H), 1.53 – 1.38 (m, 5H), 1.29 (dd, *J* = 12.8, 3.9 Hz, 1H), 1.20 (s, 3H), 1.20 – 1.10 (m, 2H), 1.07 – 1.01 (m, 1H), 1.00 (s, 3H), 0.86 (td, *J* = 13.2, 3.9 Hz, 1H), 0.59 (s, 3H). ^{13}C NMR (151 MHz, $CDCl_3$) δ 211.0, 177.3, 154.8, 116.1, 60.9, 57.1, 56.9, 56.5, 53.6, 49.9, 47.0, 44.2, 44.0, 43.9, 40.6, 38.9, 38.3, 38.2, 38.1, 29.4, 22.1, 21.3, 20.4, 19.3, 12.8. ESI-MS *m/z* 443 $[M + H]^+$.

4-(Piperazin-1-yl)butyl(4*R*,6a*S*,9*S*,11b*S*)-4,9,11b-trimethyl-7-methylene-8-oxotetradecahydro-6a,9-methanocyclohepta[a]naphthalene-4-carboxylate (31). The general procedure for the synthesis of **31** and **32** was similar to that used for **30**. Compound **31** was a colorless oil. ^1H NMR (600 MHz, MeOD) δ 6.01 (s, 1H), 5.48 (s, 1H), 4.07 (t, *J* = 6.4 Hz, 2H), 3.22 (t, *J* = 5.1 Hz, 4H), 2.71 (s, 4H), 2.50 (t, *J* = 7.2 Hz, 2H), 2.19 – 2.09 (m, 2H), 2.06 – 2.00 (m, 1H), 1.97 (dt, *J* = 13.5, 3.1 Hz, 1H), 1.85 – 1.68 (m, 6H), 1.65 – 1.53 (m, 5H), 1.44 – 1.38 (m, 3H), 1.30 (dd, *J* = 12.2, 2.2 Hz, 2H), 1.23 (s, 3H), 1.16 (ddd, *J* = 16.5, 12.2, 4.8 Hz, 1H), 1.09 (dd, *J* = 13.5, 4.0 Hz, 1H), 0.98 (s, 3H), 0.95 (dd, *J* = 13.3, 4.1 Hz, 1H), 0.65 (s, 3H). ^{13}C NMR (151 MHz, MeOD) δ 212.7, 179.0, 156.6, 116.3, 65.2, 58.5, 57.8, 57.7, 54.3, 50.9, 47.9, 45.1, 45.1, 44.8, 41.4, 39.8, 39.1, 39.0, 38.9, 29.4, 27.4, 24.2, 23.1, 22.2, 20.4, 20.1, 13.1. ESI-MS *m/z* 471 $[M + H]^+$.

10-(Piperazin-1-yl)decyl(4*R*,6a*S*,9*S*,11b*S*)-4,9,11b-trimethyl-7-methylene-8-oxotetradecahydro-6a,9-methanocyclohepta[a]naphthalene-4-carboxylate (32). Compound **32** was a colorless oil. ^1H NMR (600 MHz, $CDCl_3$) δ 6.04 (s, 1H), 5.43 (s, 1H), 4.08 – 3.97 (m, 2H), 3.52 – 3.12 (m, 4H), 3.10 – 2.68 (m, 4H), 2.66 – 2.30 (m, 2H), 2.18 (d, *J* = 13.2 Hz, 1H), 2.13 – 2.03 (m, 1H),

2.02 – 1.92 (m, 2H), 1.82 – 1.65 (m, 4H), 1.64 – 1.58 (m, 2H), 1.54 – 1.33 (m, 9H), 1.31 – 1.24 (m, 11H), 1.21 (s, 3H), 1.20 – 1.15 (m, 2H), 1.05 – 1.02 (m, 1H), 1.01 (s, 3H), 0.86 (td, *J* = 13.3, 3.9 Hz, 1H), 0.60 (s, 3H). ^{13}C NMR (151 MHz, $CDCl_3$) δ 210.9, 177.5, 154.7, 115.9, 77.4, 77.2, 76.9, 64.5, 58.1, 57.0, 56.8, 53.6, 49.8, 46.9, 44.1, 43.9, 43.4, 40.6, 38.8, 38.3, 38.2, 38.0, 29.6, 29.6, 29.5, 29.3, 29.2, 28.7, 27.3, 26.4, 22.0, 21.1, 20.3, 19.1, 12.6. ESI-MS *m/z* 555 $[M + H]^+$.

Triphenyl(3-(4-(2-(((4*R*,6a*S*,9*S*,11b*S*)-4,9,11b-trimethyl-7-methylene-8-oxotetradecahydro-6a,9-methanocyclohepta[a]naphthalene-4-carbonyl)oxy)ethyl)piperazin-1-yl)propyl)phosphonium bromide (33). To a stirred solution of compound **30** (50 mg, 0.11 mmol) in anhydrous DMF (4 mL) were added compound **12** (77 mg, 0.17 mmol) and K_2CO_3 (31 mg, 0.22 mmol), and the mixture was stirred overnight at room temperature. After TLC analysis indicated the consumption of the starting material, the reaction was carefully quenched with water. Then, CH_2Cl_2 was poured into the mixture, which was washed with brine, dried over $MgSO_4$, filtered, and concentrated under reduced pressure. The residue was then purified by flash chromatography to provide compound **33** (86 mg, 92 %) as a colorless oil. ^{1}H NMR (400 MHz, MeOD) δ 8.04 – 7.69 (m, 15H), 5.99 (s, 1H), 5.50 (d, *J* = 0.7 Hz, 1H), 4.18 (td, *J* = 5.6, 2.1 Hz, 2H), 3.52 – 3.39 (m, 2H), 2.67 (t, *J* = 5.8 Hz, 2H), 2.60 – 2.42 (m, 8H), 2.22 – 2.09 (m, 2H), 2.06 – 1.93 (m, 2H), 1.89 – 1.64 (m, 6H), 1.63 – 1.51 (m, 3H), 1.45 – 1.37 (m, 3H), 1.32 – 1.27 (m, 3H), 1.22 (s, 3H), 1.18 – 1.04 (m, 2H), 0.98 (s, 3H), 0.96 – 0.87 (m, 1H), 0.64 (s, 3H). ^{13}C NMR (101 MHz, MeOD) δ 212.8, 178.7, 156.5, 136.4 (d, *J* = 3.0 Hz), 134.8 (d, *J* = 10.0 Hz), 131.6 (d, *J* = 12.6 Hz), 119.9 (d, *J* = 86.6 Hz), 116.5, 62.4, 58.8, 58.7, 57.8, 57.7, 57.5, 54.4, 54.1, 53.8, 47.9, 45.1, 41.5, 39.8, 39.2, 39.0, 38.9, 29.4, 28.1, 23.1, 22.2, 21.0, 20.7, 20.4, 20.1, 13.3. HR-ESI-MS *m/z* calculated for $C_{48}H_{62}N_2O_3P^+$ $[M - Br]^+$ 745.4493, found 745.4498.

Triphenyl(4-(4-(2-(((4*R*,6a*S*,9*S*,11b*S*)-4,9,11b-trimethyl-7-methylene-8-oxotetradecahydro-6a,9-methanocyclohepta[a]naphthalene-4-carbonyl)oxy)ethyl)piperazin-1-yl)butyl)phosphonium bromide (34). The general procedure for the synthesis of **34 - 38** was similar to that used for **33**. Compound **34** was a colorless oil. ^{1}H NMR (400 MHz, MeOD) δ 7.95 – 7.71 (m, 15H), 6.00 (s, 1H), 5.51 (d, *J* = 0.6 Hz, 1H), 4.30 – 4.10 (m, 2H), 3.56 – 3.41 (m, 2H), 2.79 – 2.54 (m, 10H), 2.23 – 2.09 (m, 2H), 2.05 – 1.93 (m, 2H), 1.90 – 1.66 (m, 8H), 1.63 – 1.52 (m, 3H), 1.46 – 1.37 (m, 3H), 1.33 – 1.27 (m, 3H), 1.23 (s, 3H), 1.12 (ddd, *J* = 17.5, 13.5, 4.8 Hz, 2H), 0.98 (s, 3H), 0.93 (dd, *J* = 13.9, 3.4 Hz, 1H), 0.66 (s, 3H). ^{13}C NMR (101 MHz, MeOD) δ 212.8, 178.7, 156.5, 136.3

(d, *J* = 3.0 Hz), 134.9 (d, *J* = 10.0 Hz), 131.6 (d, *J* = 12.6 Hz), 119.8 (d, *J* = 86.5 Hz), 116.4, 62.3, 57.8, 57.7, 57.6, 57.4, 54.4, 53.6, 53.2, 47.9, 45.1, 41.4, 39.8, 39.2, 39.0, 38.9, 29.4, 27.4, 27.2, 23.1, 22.7, 22.2, 21.3, 20.4, 20.1, 13.4. HR-ESI-MS *m/z* calculated for $C_{49}H_{64}N_2O_3P^+$ [M - Br]$^+$ 759.4649, found 759.4652.

Triphenyl(10-(4-(2-(((4*R*,6a*S*,9*S*,11b*S*)-4,9,11b-trimethyl-7-methylene-8-oxotetradecahydro-6a,9-methanocyclohepta[a]naphthalene-4-carbonyl)oxy)ethyl)piperazin-1-yl)decyl)phosphonium bromide (35). Compound **35** was a colorless oil. ^{1}H NMR (600 MHz, MeOD) δ 7.95 – 7.85 (m, 3H), 7.85 – 7.72 (m, 12H), 6.02 (s, 1H), 5.51 (s, 1H), 4.28 – 4.08 (m, 2H), 3.46 – 3.37 (m, 2H), 2.96 – 2.61 (m, 10H), 2.22 – 2.10 (m, 2H), 2.07 – 1.93 (m, 2H), 1.87 – 1.80 (m, 1H), 1.79 – 1.74 (m, 1H), 1.73 – 1.65 (m, 4H), 1.64 – 1.59 (m, 2H), 1.59 – 1.52 (m, 5H), 1.45 – 1.38 (m, 3H), 1.36 – 1.26 (m, 13H), 1.24 (s, 3H), 1.17 (dd, *J* = 13.4, 5.6 Hz, 1H), 1.11 (td, *J* = 13.4, 4.0 Hz, 1H), 0.98 (s, 3H), 0.95 (dd, *J* = 13.3, 4.0 Hz, 1H), 0.67 (s, 3H). ^{13}C NMR (151 MHz, MeOD) δ 212.7, 178.7, 156.5, 136.3 (d, *J* = 3.0 Hz), 134.8 (d, *J* = 9.9 Hz), 131.5 (d, *J* = 12.6 Hz), 120.0 (d, *J* = 86.3 Hz), 116.4, 62.3, 58.9, 57.8, 57.7, 57.2, 54.4, 53.6, 52.5, 47.9, 45.1, 45.1, 41.5, 39.8, 39.2, 39.0, 38.9, 31.7, 31.6, 30.4, 30.3, 29.9, 29.4, 28.1, 26.2, 23.6, 23.5, 23.1, 22.8, 22.5, 22.2, 20.4, 20.1, 13.3. HR-ESI-MS *m/z* calculated for $C_{55}H_{76}N_2O_3P^+$ [M - Br]$^+$ 843.5588, found 843.5592.

Triphenyl(10-(4-(4-(((4*R*,6a*S*,9*S*,11b*S*)-4,9,11b-trimethyl-7-methylene-8-oxotetradecahydro-6a,9-methanocyclohepta[a]naphthalene-4-carbonyl)oxy)butyl)piperazin-1-yl)decyl)phosphonium (36). Compound **36** was a colorless oil. ^{1}H NMR (600 MHz, MeOD) δ 7.94 – 7.87 (m, 3H), 7.86 – 7.71 (m, 12H), 6.01 (s, 1H), 5.48 (s, 1H), 4.19 – 4.00 (m, 2H), 3.45 – 3.38 (m, 2H), 3.27 – 2.72 (m, 8H), 2.71 – 2.58 (m, 2H), 2.22 – 2.09 (m, 2H), 2.06 – 1.95 (m, 2H), 1.85 – 1.63 (m, 12H), 1.61 – 1.52 (m, 5H), 1.46 – 1.38 (m, 3H), 1.37 – 1.27 (m, 13H), 1.24 (s, 3H), 1.20 – 1.08 (m, 2H), 0.98 (s, 3H), 0.97 – 0.89 (m, 1H), 0.65 (s, 3H). ^{13}C NMR (151 MHz, MeOD) δ 212.7, 178.9, 156.6, 136.3 (d, *J* = 3.0 Hz), 134.8 (d, *J* = 9.9 Hz), 131.5 (d, *J* = 12.6 Hz), 120.0 (d, *J* = 86.3 Hz), 116.3, 65.1, 58.3, 58.0, 57.8, 57.7, 54.3, 52.8, 51.8, 47.9, 45.1, 45.1, 41.4, 39.8, 39.1, 39.0, 38.9, 31.7, 31.6, 30.4, 30.3, 30.2, 29.9, 29.4, 27.9, 27.3, 25.8, 23.6, 23.5, 23.1, 22.8, 22.5, 22.2, 20.4, 20.1, 13.2. HR-ESI-MS *m/z* calculated for $C_{57}H_{80}N_2O_3P^+$ [M - Br]$^+$ 871.5901, found 871.5905.

Triphenyl(4-(4-(10-(((4*R*,6a*S*,9*S*,11b*S*)-4,9,11b-trimethyl-7-methylene-8-oxotetradecahydro-6a,9-methanocyclohepta[a]naphthalene-4-carbonyl)oxy)decyl)piperazin-1-yl)butyl)phosphonium bromide (37). Compound **37** was a white solid, mp 91-93 °C. ^{1}H NMR (400 MHz, MeOD) δ

7.95 – 7.72 (m, 15H), 6.00 (s, 1H), 5.48 (s, 1H), 4.14 – 3.96 (m, 2H), 3.57 – 3.41 (m, 2H), 3.14 – 2.66 (m, 8H), 2.57 (t, *J* = 6.6 Hz, 2H), 2.21 – 2.08 (m, 2H), 1.07 – 1.93 (m, 2H), 1.88 – 1.49 (m, 16H), 1.48 – 1.27 (m, 17H), 1.22 (s, 3H), 1.19 – 1.05 (m, 2H), 0.98 (s, 3H), 0.94 (dd, *J* = 13.5, 4.1 Hz, 1H), 0.64 (s, 3H). ^{13}C NMR (101 MHz, MeOD) δ 212.7, 179.1, 156.6, 136.3 (d, *J* = 2.9 Hz), 134.9 (d, *J* = 10.0 Hz), 131.6 (d, *J* = 12.6 Hz), 119.9 (d, *J* = 86.4 Hz), 116.2, 65.5, 58.4, 57.8, 57.7, 57.1, 54.3, 53.0, 51.7, 47.9, 45.2, 45.1, 41.5, 39.8, 39.2, 39.1, 38.9, 30.5, 30.5, 30.3, 30.2, 29.6, 29.4, 27.9, 27.4, 25.9, 23.2, 22.7, 22.2, 21.2, 21.1, 20.5, 20.1, 13.1. HR-ESI-MS *m/z* calculated for $C_{57}H_{80}N_2O_3P^+$ [M - Br]$^+$ 871.5901, found 871.5917.

Triphenyl(10-(4-(10-(((4*R*,6a*S*,9*S*,11b*S*)-4,9,11b-trimethyl-7-methylene-8-oxotetradecahydro-6a,9-methanocyclohepta[a]naphthalene-4-carbonyl)oxy)decyl)piperazin-1-yl)decyl)phosphonium bromide (38). Compound **38** was a colorless oil. ^{1}H NMR (400 MHz, MeOD) δ 7.94 – 7.71 (m, 15H), 6.00 (s, 1H), 5.47 (s, 1H), 4.17 – 3.90 (m, 2H), 3.47 – 3.36 (m, 2H), 3.05 – 2.68 (m, 10H), 2.21 – 2.09 (m, 2H), 2.06 – 1.94 (m, 2H), 1.83 – 1.53 (m, 18H), 1.44 – 1.27 (m, 27H), 1.22 (s, 3H), 1.18 – 1.14 (m, 1H), 1.08 (dd, *J* = 13.4, 4.0 Hz, 1H), 0.98 (s, 3H), 0.94 (dd, *J* = 13.3, 4.0 Hz, 1H), 0.64 (s, 3H). ^{13}C NMR (101 MHz, MeOD) δ 212.7, 179.1, 156.6, 136.3 (d, *J* = 3.1 Hz), 134.8 (d, *J* = 10.0 Hz), 131.5 (d, *J* = 12.5 Hz), 120.0 (d, *J* = 86.3 Hz), 116.2, 65.5, 58.5, 57.8, 57.7, 54.3, 52.3, 47.9, 45.1, 45.1, 41.5, 39.8, 39.1, 39.1, 38.9, 31.7, 31.5, 30.5, 30.5, 30.4, 30.4, 30.3, 30.2, 29.9, 29.6, 29.4, 28.0, 27.4, 26.5, 26.5, 26.4, 23.6, 23.5, 23.2, 22.9, 22.4, 22.2, 20.5, 20.1, 13.1. HR-ESI-MS *m/z* calculated for $C_{63}H_{92}N_2O_3P^+$ [M - Br]$^+$ 955.6840, found 955.6842.

(5-Oxo-5-(((4*S*,5'*R*,6a*R*,6b*S*,8a*S*,8b*R*,9*S*,11a*S*,12a*S*,12b*S*)-5',6a,8a,9-tetramethyl-1,3,4,5,6,6a,6b,7,8,8a,8b,9,11a,12,12a,12b-hexadecahydrospiro[naphtho[2',1':4,5]indeno[2,1-b]furan-10,2'-piperidin]-4-yl)oxy)pentyl)triphenylphosphonium bromide (39). To a stirred solution of solasodine (30 mg, 0.07 mmol) in dry DMF (2 mL) were added compound **1** (39 mg, 0.08 mmol), HBTU (33 mg, 0.08 mmol) and DIEA (24 μL, 0.14 mmol). The reaction mixture was stirred overnight at room temperature, after which TLC analysis indicated the consumption of the starting material. Next, 10 mL of water was added to the resulting solution, which was then extracted with EtOAc (10 mL × 3). The combined extracts were washed with brine, dried over $MgSO_4$, and evaporated under vacuum to obtain the crude product, which was purified by flash chromatography to afford compound **39** (44 mg, 71 %) as a white solid, mp 181-183 °C. ^{1}H NMR (600 MHz, MeOD) δ 7.99 – 7.71 (m, 15H), 5.37 (s, 1H), 4.65 (s, 1H), 4.55 – 4.43 (m, 1H), 3.51 –

3.41 (m, 2H), 3.35 (s, 1H), 3.06 (d, J = 11.3 Hz, 1H), 2.87 (t, J = 12.1 Hz, 1H), 2.44 – 2.33 (m, 3H), 2.28 – 2.21 (m, 2H), 2.14 – 2.01 (m, 2H), 1.99 – 1.80 (m, 8H), 1.79 – 1.68 (m, 5H), 1.57 (dt, J = 22.8, 13.2 Hz, 5H), 1.46 – 1.22 (m, 4H), 1.19 (d, J = 2.2 Hz, 3H), 1.12 (t, J = 13.6 Hz, 1H), 1.06 (s, 3H), 0.99 (d, J = 3.6 Hz, 3H), 0.87 (s, 3H). ^{13}C NMR (151 MHz, MeOD) δ 174.0, 141.0, 136.3 (d, J = 2.8 Hz), 134.8 (d, J = 10.0 Hz), 131.6 (d, J = 12.6 Hz), 123.3, 119.8 (d, J = 86.5 Hz), 100.2, 84.6, 75.4, 63.0, 57.5, 51.3, 46.7, 42.9, 42.1, 40.3, 39.1, 38.1, 37.8, 34.3, 33.3, 33.1, 33.0, 32.7, 29.0, 28.8, 26.9, 22.9, 22.6, 22.3, 21.8, 19.7, 18.7, 16.5, 14.9. HR-ESI-MS m/z calculated for $C_{50}H_{65}NO_3P^+$ $[M - Br]^+$ 758.4697, found 758.4693.

(5-((2-((5-(Dimethylamino)naphthalene)-1-sulfonamido)ethyl)amino)-5-oxopentyl)triphenylphosphonium bromide (41). To a stirred solution of ethylenediamine (5.6 mL, 82.7 mmol) in dry CH_2Cl_2 (50 mL) in an ice bath, dansyl chloride (500 mg, 1.8 mmol) in dry CH_2Cl_2 (20 mL) was added dropwise, and the reaction mixture was allowed to stir at room temperature for 1 h. After TLC analysis indicated the consumption of the starting material, the reaction was quenched with water. Then, CH_2Cl_2 was poured into the mixture, which was washed with brine, dried over $MgSO_4$, filtered, and concentrated under reduced pressure to give compound **40**. Compound **40** was directly used without further purification. Compound **1** (50 mg, 0.11 mmol) was dissolved in anhydrous DMF (3 mL), HBTU (43 mg, 0.11 mmol) and DIPEA (23 μL, 0.14 mmol) were added to the resulting solution, and the mixture was stirred for 20 min. Then, compound **40** (66 mg, 0.22 mmol) in DMF (1 mL) was added dropwise to the mixture in an ice bath, and the mixture was allowed to stir overnight at room temperature. After TLC analysis indicated the consumption of the starting material, the resulting mixture was quenched with water and extracted three times with CH_2Cl_2. The combined extracts were washed with brine, dried over $MgSO_4$, and filtered, and the solvent was removed under reduced pressure to afford the crude product. The residue was subjected to chromatography on silica gel to yield compound **41** (121 mg, 75 %) as a yellow solid, mp 85-87 °C. ^{1}H NMR (400 MHz, $CDCl_3$) δ 8.49 (d, J = 8.5 Hz, 1H), 8.26 (d, J = 8.6 Hz, 1H), 8.14 (d, J = 7.2 Hz, 1H), 7.84 – 7.57 (m, 15H), 7.49 (dt, J = 15.9, 8.1 Hz, 2H), 7.13 (d, J = 7.5 Hz, 1H), 6.80 (t, J = 5.4 Hz, 1H), 6.30 (t, J = 5.1 Hz, 1H), 3.26 – 3.07 (m, 4H), 2.97 – 2.91 (m, 2H), 2.84 (s, 6H), 2.18 (t, J = 6.8 Hz, 2H), 1.88 – 1.77 (m, 2H), 1.74 – 1.62 (m, 2H). ^{13}C NMR (101 MHz, $CDCl_3$) δ 174.0, 151.9, 135.3 (d, J = 2.8 Hz), 135.0, 133.5 (d, J = 9.9 Hz), 130.7 (d, J = 12.6 Hz), 130.3, 129.9, 129.6, 129.4, 128.5, 123.3, 119.3, 117.9 (d, J = 86.2 Hz), 115.4, 45.5, 43.7,

39.5, 34.4, 26.1, 25.9, 22.2, 21.7, 21.5. HR-ESI-MS m/z calculated for $C_{37}H_{41}N_3O_3PS^+$ [M - Br]$^+$ 638.2601, found 638.2596.

(4-((2-(6-(Diethylamino)-3-(diethyliminio)-3H-xanthen-9-yl)benzoyl)oxy)butyl)triphenylphosphonium bromide chloride (42). The general procedure for the synthesis of **42** was similar to that used for **17**, and the product was purified by HPLC. The experiment was performed using mobile phase A composed of 0.01 % trifluoroacetic acid in water and mobile phase B composed of acetonitrile. The HPLC gradient system started with 50 % B and linearly increased to 100 % in 20 min. ESI-MS *m/z* 380.50 [(M - Cl - Br)/2]$^+$.

(3-Bromo-2-hydroxypropyl)triphenylphosphonium bromide (43). The general procedure for the synthesis of **43** was similar to that used for **12**, and the product was used directly without further purification.

(2-Hydroxy-3-(((4*R*,6a*S*,9*S*,11b*S*)-4,9,11b-trimethyl-7-methylene-8-oxotetradecahydro-6a,9-methanocyclohepta[a]naphthalene-4-carbonyl)oxy)propyl)triphenylphosphonium bromide (44). The general procedure for the synthesis of **44** was similar to that used for **17**. Compound **44** was a colorless oil. ^{1}H NMR (400 MHz, MeOD) δ 7.92 – 7.79 (m, 18H), 7.77 – 7.69 (m, 12H), 5.87 (d, *J* = 28.2 Hz, 2H), 5.43 (d, *J* = 33.8 Hz, 2H), 4.25 (ddd, *J* = 11.3, 3.8, 2.1 Hz, 1H), 4.20 – 4.00 (m, 5H), 3.93 – 3.77 (m, 2H), 3.61 – 3.50 (m, 2H), 2.44 – 2.31 (m, 1H), 2.26 – 2.09 (m, 4H), 2.03 – 1.97 (m, 2H), 1.93 (dt, *J* = 13.3, 3.0 Hz, 1H), 1.89 – 1.64 (m, 9H), 1.63 – 1.51 (m, 6H), 1.45 – 1.35 (m, 7H), 1.32 – 1.29 (m, 5H), 1.25 (s, 3H), 1.17 – 1.07 (m, 4H), 0.99 (s, 3H), 0.98 (s, 3H), 0.93 (dd, *J* = 13.6, 3.6 Hz, 2H), 0.61 (s, 3H), 0.58 (s, 3H). ^{13}C NMR (101 MHz, MeOD) δ 212.9, 212.8, 179.0, 178.5, 156.4, 156.0, 136.1, 136.0, 135.2, 135.2, 135.1, 135.1, 131.3, 131.3, 131.2, 131.2, 121.1, 121.0, 120.3, 120.2, 117.0, 116.4, 65.5, 65.5, 57.8, 57.8, 57.8, 57.7, 54.4, 54.3, 49.8, 47.9, 47.9, 45.3, 45.2, 45.1, 41.3, 41.3, 39.8, 39.8, 39.3, 39.1, 39.1, 38.9, 29.3, 29.0, 23.3, 23.1, 22.2, 20.5, 20.4, 20.2, 19.9, 13.2, 12.9. HR-ESI-MS *m/z* calculated for $C_{42}H_{50}O_4P^+$ [M - Br]$^+$ 649.3441, found 649.3443.

2-(2-Bromoethoxy)ethyl(4*R*,6a*S*,9*S*,11b*S*)-4,9,11b-trimethyl-7-methylene-8-oxotetradecahydro-6a,9-methanocyclohepta[a]naphthalene-4-carboxylate (45). To a stirred solution of **11** (30 mg, 0.09 mmol) in anhydrous DMF (3 mL) were added 2,2'-dibromodiethyl ether (114 μL, 0.9 mmol) and K_2CO_3 (25 mg, 0.18 mmol), and the mixture was stirred overnight at room temperature. After TLC analysis indicated the consumption of the starting material, the reaction was quenched

with water. Then, CH_2Cl_2 was added to the mixture, which was washed with brine, dried over $MgSO_4$, filtered, and concentrated under reduced pressure to afford compound **45** as a colorless oil in quantitative yield. ^{1}H NMR (400 MHz, $CDCl_3$) δ 6.05 (s, 1H), 5.49 (s, 1H), 4.20 (t, *J* = 4.7 Hz, 2H), 3.79 (td, *J* = 6.2, 1.7 Hz, 2H), 3.71 (dd, *J* = 9.5, 4.5 Hz, 2H), 3.44 (t, *J* = 6.2 Hz, 2H), 2.26 – 2.06 (m, 2H), 2.03 – 1.92 (m, 2H), 1.87 – 1.64 (m, 4H), 1.56 – 1.38 (m, 5H), 1.30 (dd, *J* = 12.9, 3.6 Hz, 1H), 1.23 (s, 3H), 1.20 (dd, *J* = 12.3, 2.6 Hz, 1H), 1.05 (dd, *J* = 13.5, 4.1 Hz, 1H), 1.01 (s, 3H), 0.86 (td, *J* = 13.2, 4.5 Hz, 2H), 0.62 (s, 3H). ^{13}C NMR (101 MHz, $CDCl_3$) δ 210.9, 177.4, 154.6, 116.1, 71.1, 69.1, 63.1, 57.0, 56.9, 53.6, 46.9, 44.1, 43.9, 40.5, 38.8, 38.3, 38.2, 38.0, 30.2, 29.1, 21.9, 21.2, 20.3, 19.1, 12.5. ESI-MS *m/z* 481 $[M + H]^+$.

Triphenyl(2-(2-(((4*R*,6a*S*,9*S*,11b*S*)-4,9,11b-trimethyl-7-methylene-8-oxotetradecahydro-6a,9-methanocyclohepta[a]naphthalene-4-carbonyl)oxy)ethoxy)ethyl)phosphonium bromide (46). The general procedure for the synthesis of **46** was similar to that used for **28** as a colorless oil. ^{1}H NMR (600 MHz, MeOD) δ 7.91 – 7.86 (m, 3H), 7.84 – 7.78 (m, 6H), 7.74 (ddd, *J* = 8.2, 3.6, 2.0 Hz, 6H), 5.96 (s, 1H), 5.44 (s, 1H), 3.94 (t, *J* = 4.6 Hz, 2H), 3.85 (dt, *J* = 20.4, 5.7 Hz, 2H), 3.80 – 3.74 (m, 2H), 3.49 – 3.39 (m, 2H), 2.15 – 2.05 (m, 2H), 2.01 – 1.93 (m, 2H), 1.74 (ddd, *J* = 13.4, 8.7, 3.8 Hz, 2H), 1.70 – 1.66 (m, 2H), 1.60 – 1.53 (m, 3H), 1.40 (dd, *J* = 11.5, 2.6 Hz, 2H), 1.28 (dd, *J* = 12.6, 2.5 Hz, 2H), 1.17 (s, 3H), 1.14 (dd, *J* = 13.4, 5.6 Hz, 1H), 1.09 (td, *J* = 13.4, 4.0 Hz, 1H), 0.99 (s, 3H), 0.95 (dd, *J* = 13.1, 4.0 Hz, 1H), 0.58 (s, 3H). ^{13}C NMR (151 MHz, MeOD) δ 212.8, 178.5, 156.6, 136.2 (d, *J* = 3.0 Hz), 135.0 (d, *J* = 10.3 Hz), 131.4 (d, *J* = 12.8 Hz), 120.3 (d, *J* = 87.3 Hz), 116.3, 70.4, 64.9, 64.8,63.9, 57.7, 57.6, 54.3, 47.9, 45.1, 45.1, 41.3, 39.8, 39.1, 38.9, 38.9, 29.4, 25.3, 25.0, 23.0, 22.2, 20.4, 20.2, 13.2. HR-ESI-MS *m/z* calculated for $C_{43}H_{52}O_4P^+$ $[M - Br]^+$ 663.3598, found 663.3601.

2-(2-(2-(Tosyloxy)ethoxy)ethoxy)ethyl(4*R*,6a*S*,9*S*,11b*S*)-4,9,11b-trimethyl-7-methylene-8-oxo tetradecahydro-6a,9-methanocyclohepta[a]naphthalene-4-carboxylate (47). Compound **11** (33 mg, 0.1 mmol) was dissolved in dry DMF (3 mL). Triethylene glycol bis(p-toluenesulfonate) (230 mg, 0.5 mmol) and K_2CO_3 (27.6 mg, 0.2 mmol) were added, and the mixture was stirred for 4 h at room temperature. After TLC analysis indicated the consumption of the starting material, the reaction was quenched with water. Next, the mixture was extracted with ethyl acetate and washed twice with a saturated aqueous sodium chloride solution. The organic layer was dried over $MgSO_4$, filtered, and concentrated under reduced pressure. The residue was purified by flash

chromatography to give compound **47** (52 mg, 85 %) as a colorless oil. ^{1}H NMR (400 MHz, $CDCl_3$) δ 7.79 (d, *J* = 8.3 Hz, 2H), 7.34 (d, *J* = 8.0 Hz, 2H), 6.03 (s, 1H), 5.47 (s, 1H), 4.24 – 4.10 (m, 4H), 3.74 – 3.62 (m, 4H), 3.57 (s, 4H), 2.45 (s, 3H), 2.23 – 2.06 (m, 2H), 2.02 – 1.91 (m, 2H), 1.85 – 1.64 (m, 4H), 1.55 – 1.38 (m, 5H), 1.30 (dd, *J* = 12.9, 3.7 Hz, 1H), 1.22 (s, 3H), 1.19 (dd, *J* = 12.3, 2.7 Hz, 2H), 1.08 – 1.03 (m, 1H), 1.02 (s, 3H), 0.86 (td, *J* = 13.2, 3.9 Hz, 1H), 0.61 (s, 3H). ^{13}C NMR (101 MHz, $CDCl_3$) δ 210.9, 177.4, 154.6, 145.0, 133.1, 130.0, 128.1, 116.1, 77.4, 70.9, 70.5, 69.3, 68.9, 63.2, 57.0, 56.8, 53.6, 46.9, 44.1, 43.9, 40.5, 38.8, 38.3, 38.2, 38.0, 29.1, 21.9, 21.8, 21.2, 20.3, 19.1, 12.5. ESI-MS *m/z* 617 $[M + H]^+$.

Triphenyl(2-(2-(2-(((4*R*,6a*S*,9*S*,11b*S*)-4,9,11b-trimethyl-7-methylene-8-oxotetradecahydro-6a,9-methanocyclohepta[a]naphthalene-4-carbonyl)oxy)ethoxy)ethoxy)ethyl)phosphonium bromide (49). Compound **47** (41 mg, 0.07 mmol) was dissolved in acetone (4 mL). Lithium bromide hydrate (8.5 mg, 0.08 mmol) was added, and the mixture was stirred under reflux for 48 h. After TLC analysis indicated the consumption of the starting material, the reaction was quenched with water. Next, the mixture was extracted with ethyl acetate and washed twice with a saturated aqueous sodium chloride solution. The organic layer was dried over $MgSO_4$, filtered, and concentrated under reduced pressure to give crude product **48**, which was used directly without further purification. Compound **48** was dissolved in anhydrous toluene (4 mL), and triphenylphosphine (53 mg, 0.20 mmol) was added at room temperature. Then, the mixture was allowed to stir under reflux for 24 h. After TLC analysis indicated the consumption of the starting material, the solvent was removed under reduced pressure, and the residue was purified by flash chromatography to provide compound **49** (17 mg, 33 %) as a colorless oil. ^{1}H NMR (600 MHz, MeOD) δ 7.89 – 7.80 (m, 9H), 7.74 (td, *J* = 7.9, 3.4 Hz, 6H), 5.95 (s, 1H), 5.48 (s, 1H), 4.12 (ddd, *J* = 11.4, 6.3, 3.2 Hz, 1H), 4.03 (ddd, *J* = 12.0, 5.8, 3.2 Hz, 1H), 3.82 (t, *J* = 5.8 Hz, 1H), 3.80 – 3.76 (m, 2H), 3.75 (t, *J* = 5.8 Hz, 1H), 3.53 – 3.43 (m, 2H), 3.35 (s, 4H), 2.21 – 2.12 (m, 2H), 2.02 – 1.92 (m, 2H), 1.86 – 1.72 (m, 2H), 1.68 (d, *J* = 12.7 Hz, 2H), 1.61 – 1.52 (m, 3H), 1.45 – 1.37 (m, 3H), 1.36 – 1.32 (m, 1H), 1.22 (s, 3H), 1.11 (ddd, *J* = 17.5, 13.7, 4.8 Hz, 2H), 0.99 (s, 3H), 0.95 (td, *J* = 13.2, 4.2 Hz, 1H), 0.62 (s, 3H). ^{13}C NMR (151 MHz, MeOD) δ 211.4, 177.5, 155.1, 134.7 (d, *J* = 3.2 Hz), 133.7 (d, *J* = 10.3 Hz), 129.9 (d, *J* = 12.8 Hz), 119.1 (d, *J* = 87.4 Hz), 115.1, 70.2, 69.6, 68.6, 63.4, 63.0, 56.4, 56.3, 52.9, 46.5, 43.7, 40.0, 38.4, 37.8, 37.7, 37.5, 27.9, 23.9,

23.6, 21.7, 20.8, 19.0, 18.7, 11.8. HR-ESI-MS *m/z* calculated for $C_{45}H_{56}O_5P^+$ [M - Br]$^+$ 707.3860, found 707.3865.

X-ray Crystallographic Analysis of Compounds 6.

A single crystal of compound **6** was studied. A suitable crystal was selected in each case and examined using a D8 Venture diffractometer. The crystal was maintained at 296 K during data collection. Using Shelxl-2014/7, the structure was solved with the ShelXS structure solution program using direct methods and refined with the ShelXL refinement package using least-squares minimization [15].

The parameters for the crystal structure of compound **6** ($C_{21}H_{34}O_4$, M = 350.25 g/mol) were as follows: monoclinic, space group $P2_1$, a = 12.1395(9) Å, b = 7.3559(6) Å, c = 12.7507(8) Å, α = 90°, β = 114.334°, V = 1037.44(14) Å^3, Z = 2, T = 296(2) K, μ (Cu Kα) = 1.54178 mm^{-1}, $Dcalc$ = 1.218 g/cm^3, 3983 reflections measured (3.805° ≤ 2θ ≤ 72.446°), and 2845 unique, which were used in all calculations. The final R_1 was 0.1042 (>2sigma(I)), and wR_2 was 0.2737 (all data). The Flack parameter was 0.0(3). Details of the crystallographic data (excluding structure factors) for **6** have been deposited with the Cambridge Crystallographic Data Centre as supplementary publication number CCDC 1813899. The data can be obtained free of charge via www.ccdc.cam.ac.uk/products/csd/request.

Supplementary References

1. Schubert, S. et al. Regulation of efflux pump expression and drug resistance by the transcription factors Mrr1, Upc2, and Cap1 in *Candida albicans*. *Antimicrob. Agents Chemother.* **55,** 2212-2223 (2011).

2. Dunkel, N. et al. A gain-of-function mutation in the transcription factor Upc2p causes upregulation of ergosterol biosynthesis genes and increased fluconazole resistance in a clinical *Candida albicans* isolate. *Eukaryot. Cell* **7,** 1180-1190 (2008).

3. Franz, R., Ruhnke, M. & Morschhäuser, J. Molecular aspects of fluconazole resistance development in *Candida albicans*. *Mycoses* **42,** 453-458 (1999).

4. Sanglard, D. et al. Mechanisms of resistance to azole antifungal agents in *Candida albicans* isolates from AIDS patients involve specific multidrug transporters. *Antimicrob. Agents Chemother.* **39,** 2378-2386 (1995).

5. Franz, R. et al. Multiple molecular mechanisms contribute to a stepwise development of fluconazole resistance in clinical *Candida albicans* strains. *Antimicrob. Agents Chemother.* **42,** 3065-3072 (1998).

6. Sanglard, D., Ischer, F., Monod, M. & Bille, J. Cloning of *Candida albicans* genes conferring resistance to azole antifungal agents: characterization of *CDR2*, a new multidrug ABC transporter gene. *Microbiology* **143,** 405-416 (1997).

7. Sanglard, D., Ischer, F., Monod, M. & Bille, J. Susceptibilities of *Candida albicans* multidrug transporter mutants to various antifungal agents and other metabolic inhibitors. *Antimicrob. Agents Chemother.* **40,** 2300-2305 (1996).

8. Mukherjee, P. K., Chandra, J., Kuhn, D. M. & Ghannoum, M. A. Mechanism of fluconazole resistance in *Candida albicans* biofilms: phase-specific role of efflux pumps and membrane sterols. *Infect. Immun.* **71,** 4333-4340 (2003).

9. Otzen, T. et al. Folate-synthesizing enzyme system as target for development of inhibitors and inhibitor combinations against *Candida albicans* Synthesis and Biological Activity of New 2,4-Diaminopyrimidines and 4'-Substituted 4-Aminodiphenyl Sulfones. *J. Med. Chem.* **47,** 240-253 (2004).

10. Geraminejad, M., Dulmage, K. & Berman, J. Additional cassettes for epitope and fluorescent fusion proteins in *Candida albicans*. *Yeast* **26,** 399-406 (2009).

11. Gerami-Nejad, M., Berman, J. & Gale, C. A. Cassettes for PCR-mediated construction of green, yellow, and cyan fluorescent protein fusions in *Candida albicans*. *Yeast* **18,** 859-864 (2001).

12. Wayne, P. et al. Clinical and Laboratory Standards Institute: Reference method for broth dilution antifungal susceptibility testing of yeasts; approved standard, CLSI document M27-A3 and Supplement S, 3 (2008).

13. Breger, J. et al. Antifungal chemical compounds identified using a *C. elegans* pathogenicity assay. *PLoS Pathog.* **3,** e18 (2007).

14. Chang, W., Li, Y., Zhang, L., Cheng, A. & Lou, H. Retigeric acid B attenuates the virulence of *Candida albicans* via inhibiting adenylyl cyclase activity targeted by enhanced farnesol production. *PLoS One* **7,** e41624 (2012).

15. Sheldrick, G. M. *Acta. Cryst.* **64,** 112−122 (2008).
